# Supplementary material for: Age and Infectious Dose Significantly Affect Disease Progression after RHDV2 Infection in Naïve Domestic Rabbits
Source: Viruses. 2021 Jun 21;13(6):1184. doi: 10.3390/v13061184 (PMC8234499; doi:10.3390/v13061184)
Supplement: Supplementary file 1 [file viruses-13-01184-s001.zip › viruses-1243626-supplementary.pdf]

Age and infectious dose significantly affect disease progression after RHDV2 infection in naïve domestic rabbits

Supplementary data

Robyn N. Hall[1,2*], Tegan King[1], Tiffany O'Connor[3], Andrew J. Read[3], Jane Arrow[4], Katherine Trought[4], Janine Duckworth[4], Melissa Piper[5], Tanja Strive[1,2]

1. Health & Biosecurity, Commonwealth Scientific and Industrial Research Organisation, Acton, ACT, Australia
2. Centre for Invasive Species Solutions, Bruce, ACT, Australia
3. Elizabeth Macarthur Agricultural Institute, NSW Department of Primary Industries, Menangle, NSW, Australia
4. Wildlife Ecology and Management, Manaaki Whenua – Landcare Research, Lincoln, New Zealand
5. Agriculture & Food, Commonwealth Scientific and Industrial Research Organisation, Acton, ACT, Australia

* Corresponding author Robyn.Hall@csiro.au

Supplementary tables

Supplementary Table 1. Experimental design for the assessment of RHDV2 virulence.

Trial	Number of animals†	Age (weeks)	Sex (F:M)	Target inoculum dose (RID_{50})‡	Actual inoculum dose (capsid gene copies)
1	4 + 1 control	11	2:3	1000	1×10^7
2	6 + 1 control	11	2:5	1000	7×10^7
3	2 + 1 control	11	1:2	1000	6×10^6
	4		2:2	50	3×10^5
4	8 + 1 control	11	4:5	50	4×10^5
5	6 + 1 control	5	1:6	1000	1×10^7
6	6 + 1 control	5	4:3	1000	1×10^7
	1		0:1	50	7×10^5
7	6 + 1 control	5	4:4	50	3×10^5
8	5 + 1 control	5	1:5	50	7×10^5

† Each trial included one uninfected control animal from the respective litter group. ‡ RID_{50} 50% rabbit infectious dose.

Supplementary Table 2. Statistical analyses of disease progression after RHDV2 infection. Significance testing was conducted using the two-sided Wilcoxon signed rank test. All p values are given to two significant figures. The effect size refers to the difference between the means of the comparison groups.

Comparison groups	p	Effect size (hrs)	Standard deviation (hrs)	
			Group 1	Group 2
Survival time				
High dose, adult vs kitten	0.000079	10.0	5.2	2.4
Low dose, adult vs kitten	0.00013	16.4	8.1	5.8
Adults, high dose vs low dose	0.0015	11.4	5.2	8.1
Kittens, high dose vs low dose	0.062	5.0	2.4	5.8
Time to onset of pyrexia				
High dose, adult vs kitten	0.0015	7.6	5.6	2.2
Low dose, adult vs kitten	0.00015	11.2	4.2	4.3
Adults, high dose vs low dose	0.0050	8.0	5.6	4.2
Kittens, high dose vs low dose	0.0089	4.4	2.2	4.3
Duration of clinical signs of disease				
High dose, adult vs kitten	0.059	3.3	4.7	1.6
Low dose, adult vs kitten	0.030	5.2	5.7	2.4
Adults, high dose vs low dose	0.35	2.4	4.7	5.7
Kittens, high dose vs low dose	0.60	0.5	1.6	2.4

Supplementary figures

Disease course in individual animals. Bodyweight (green points and green dashed line) was recorded at least twice daily. Each animal was fitted with a monitoring collar. A continuous temperature logger was attached to the collar and held tightly against the skin to record external body temperature (thin red line) every 12 minutes. To smooth noise in the temperature data, a smoothed conditional mean (thick red line) was plotted using local polynomial regression fitting as implemented in ggplot2 (geom_smooth(method = "loess",se = T) with span = 0.05). An activity tracker was attached to the same collar as the temperature monitor. In trials 1 to 3, we used Tuokiy activity trackers that generated a reading from the 3D accelerometer every 20 minutes. We encountered repeated technical issues with these trackers, particularly after an app update following trial 3. Consequently, no activity data were able to be recovered from trial 4. We subsequently switched to FitBark2 activity monitors for trials 5 to 8, which generated a 3D accelerometer reading every minute. A moving average of the activity data (orange line) was calculated to smooth noise, with a backwards window length of 255 (FitBark) or 16 (Tuokiy), using the pracma package. The accelerometer readings are in arbitrary units for both devices. Blood samples were collected at several timepoints to monitor viraemia levels (blue points and blue dashed line) over time. Total RNA was extracted from whole blood and virus capsid gene copies per mg blood were quantified by RT-qPCR. Anomalies and dropouts in the collar data (external body temperature and activity) occurred for some animals over some time periods as collars became loose or were scratched off. We have removed these dropouts to improve visualisation and have provided additional information in the caveats below each image.

Adult, control

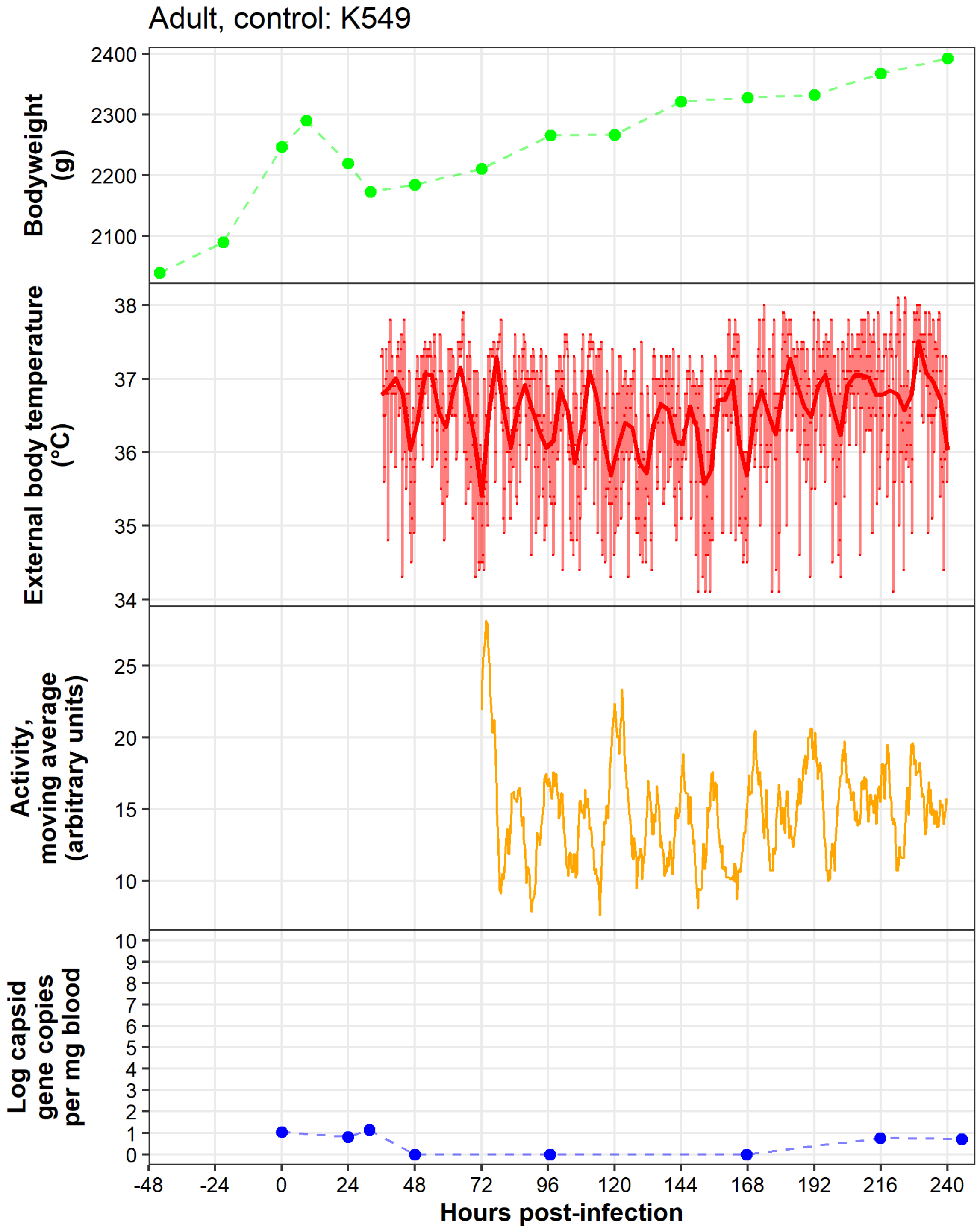

Caveats: Temperature monitor fell out of pouch between -48–32 hpi. Experienced technical issues with activity monitor prior to 72 hpi.

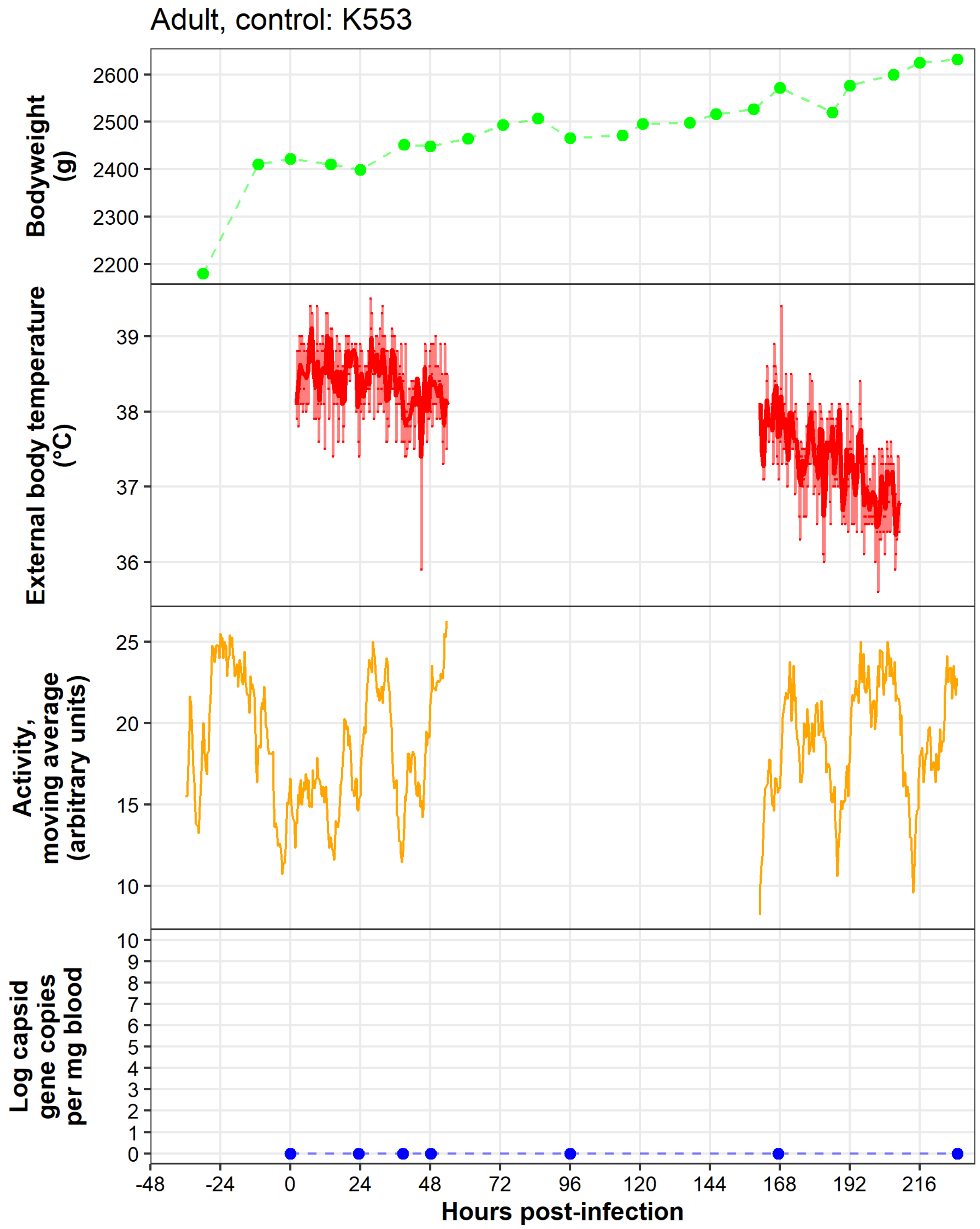

Caveats: Temperature monitor fell out of pouch, replaced at 0 hpi. Collar removed between 54–161 hpi. Temperature monitor missing after 209 hpi. Euthanised 229 hpi rather than 240 hpi as per other controls (still 10 dpi).

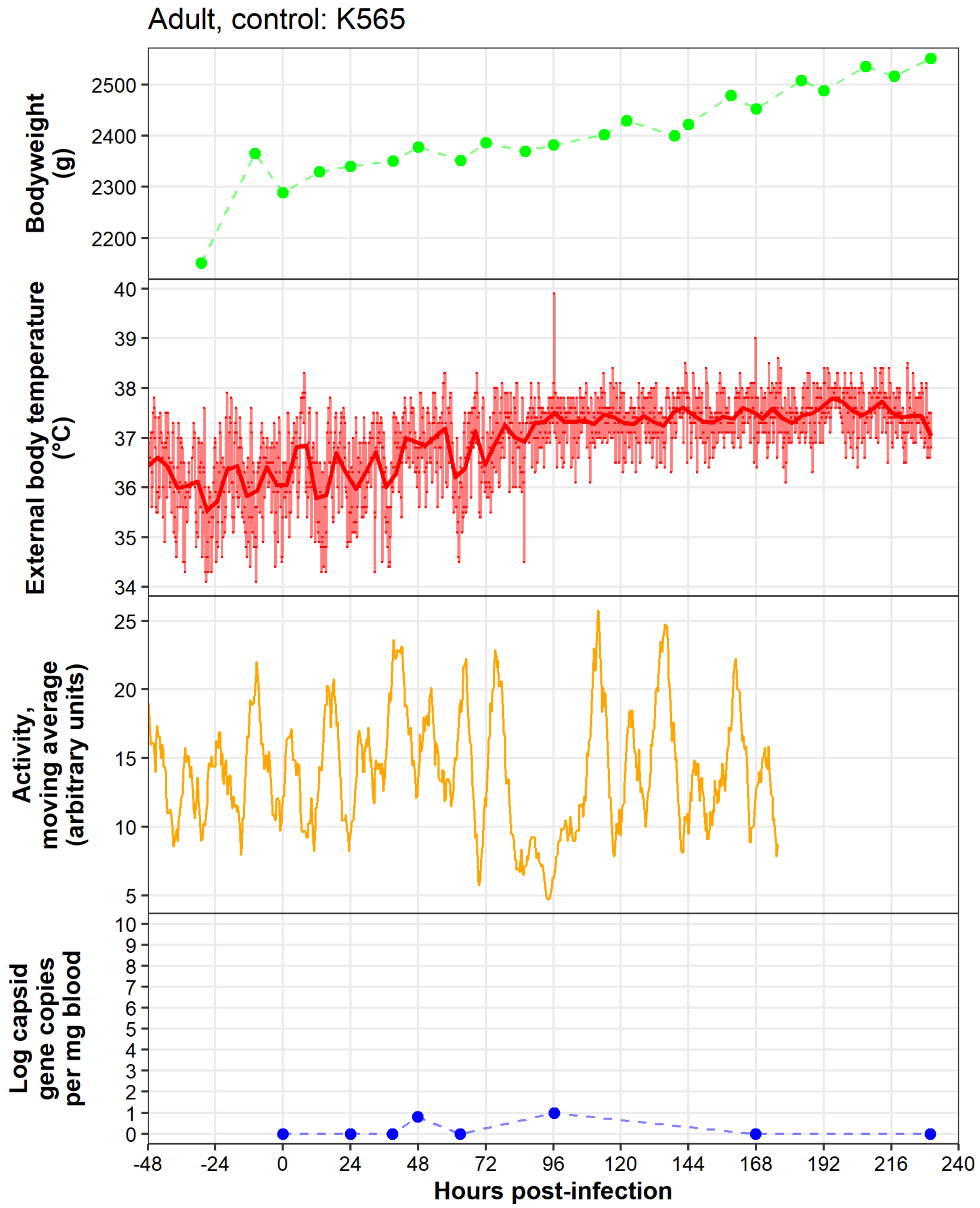

Caveats: Technical difficulties with activity monitor after 176 hpi.

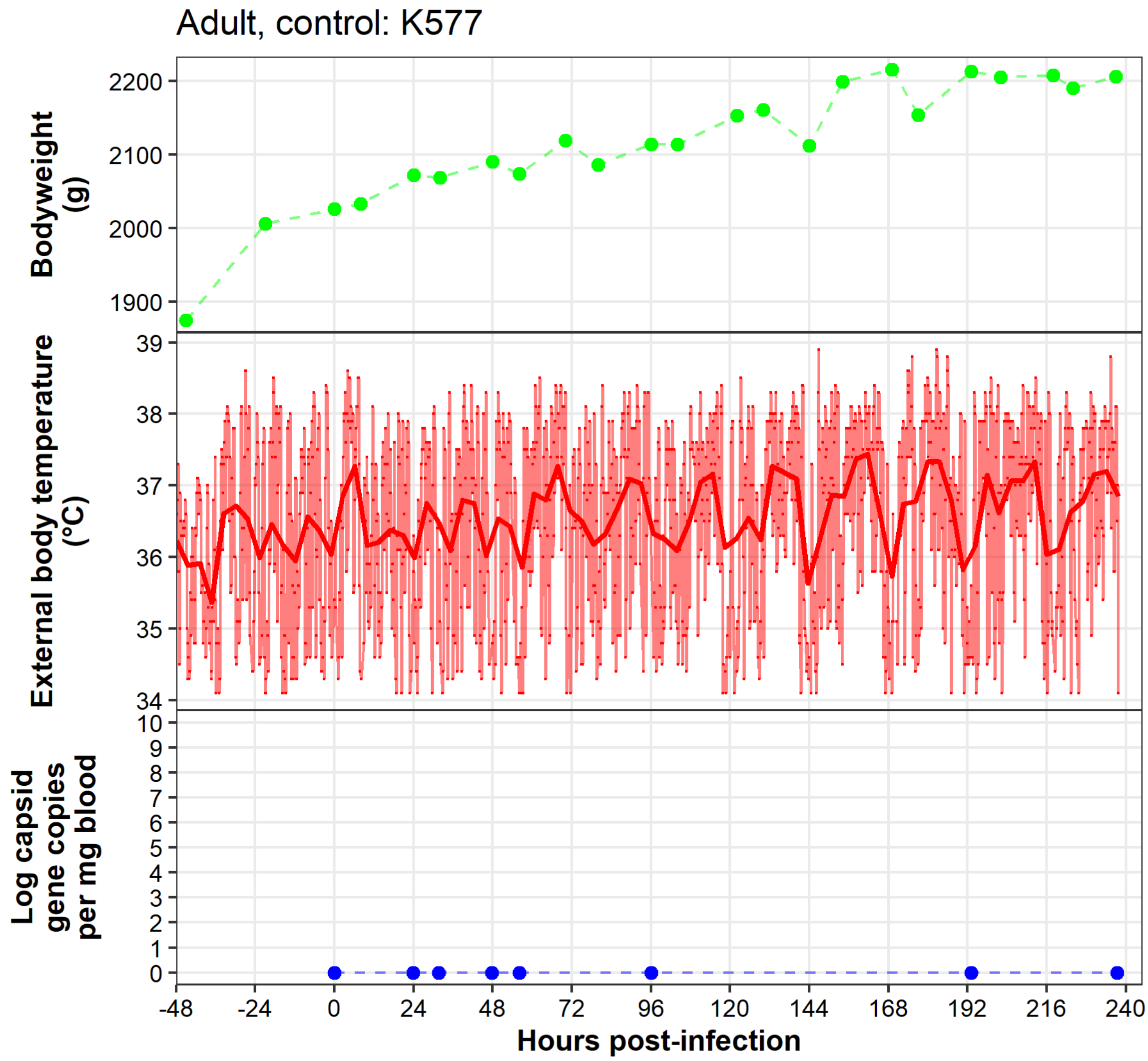

Caveats: Activity monitors were not used in trial 4 due to technical issues with the app update.

Adult, high dose

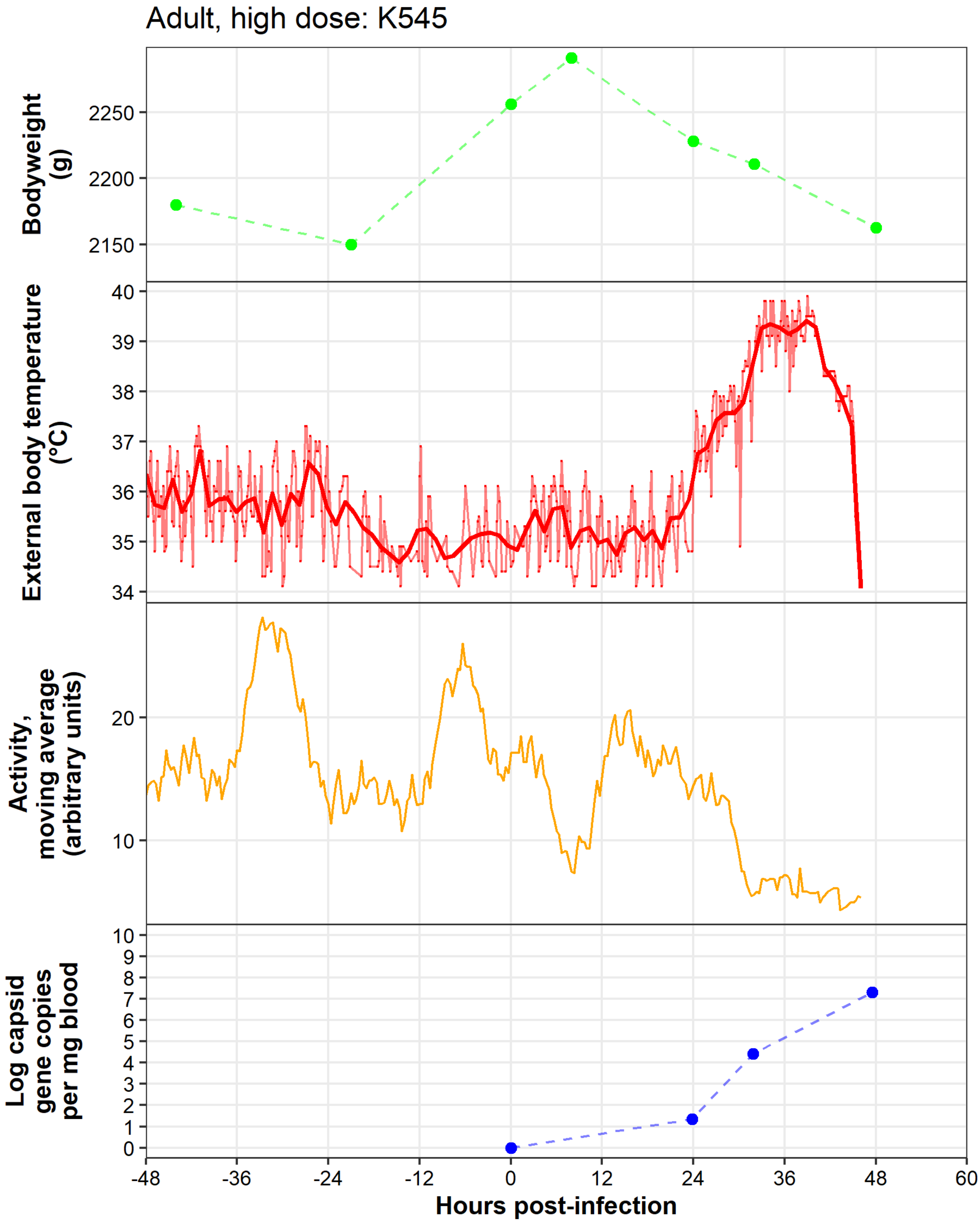

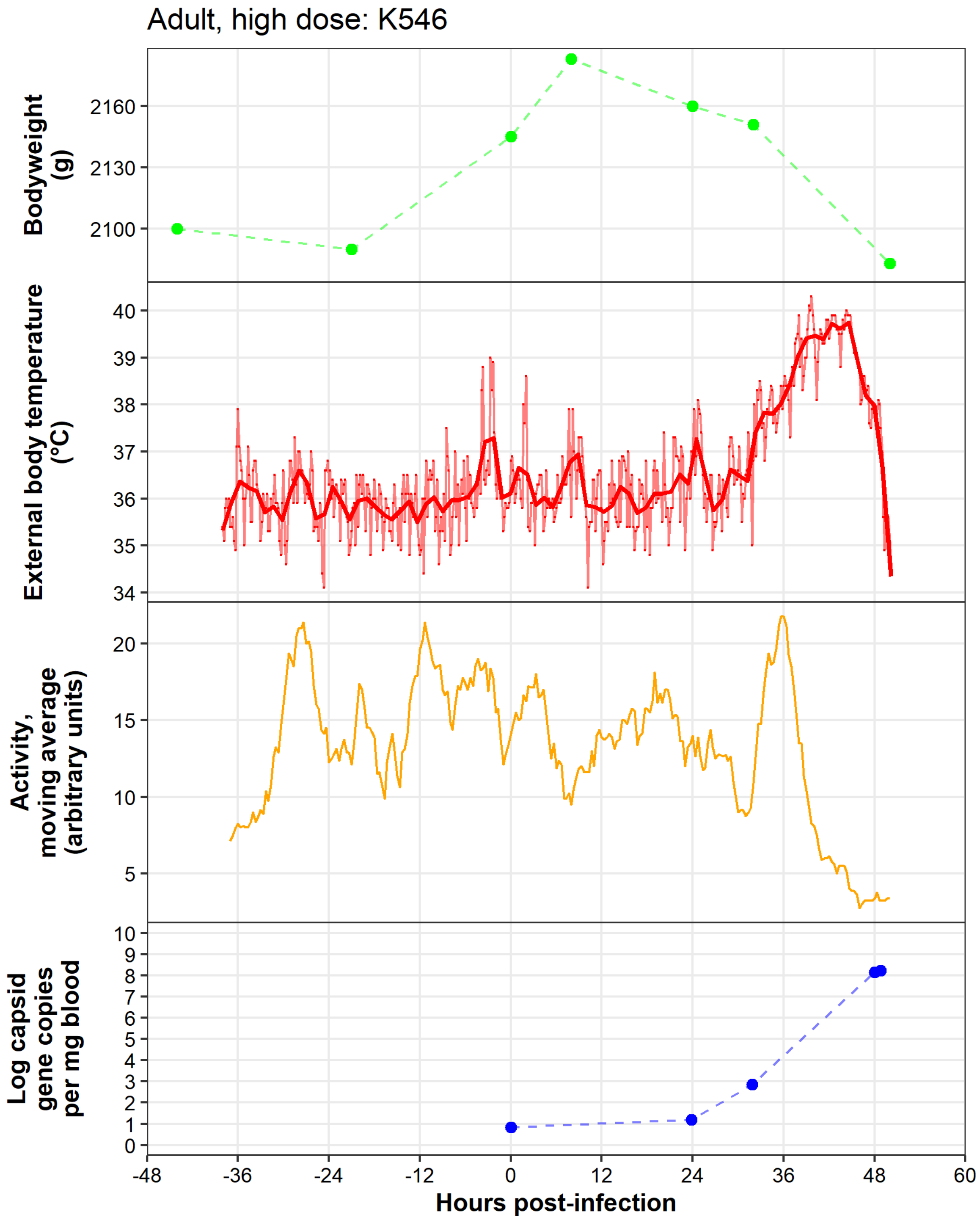

Caveats: Technical difficulties with activity monitor before -36 hpi.

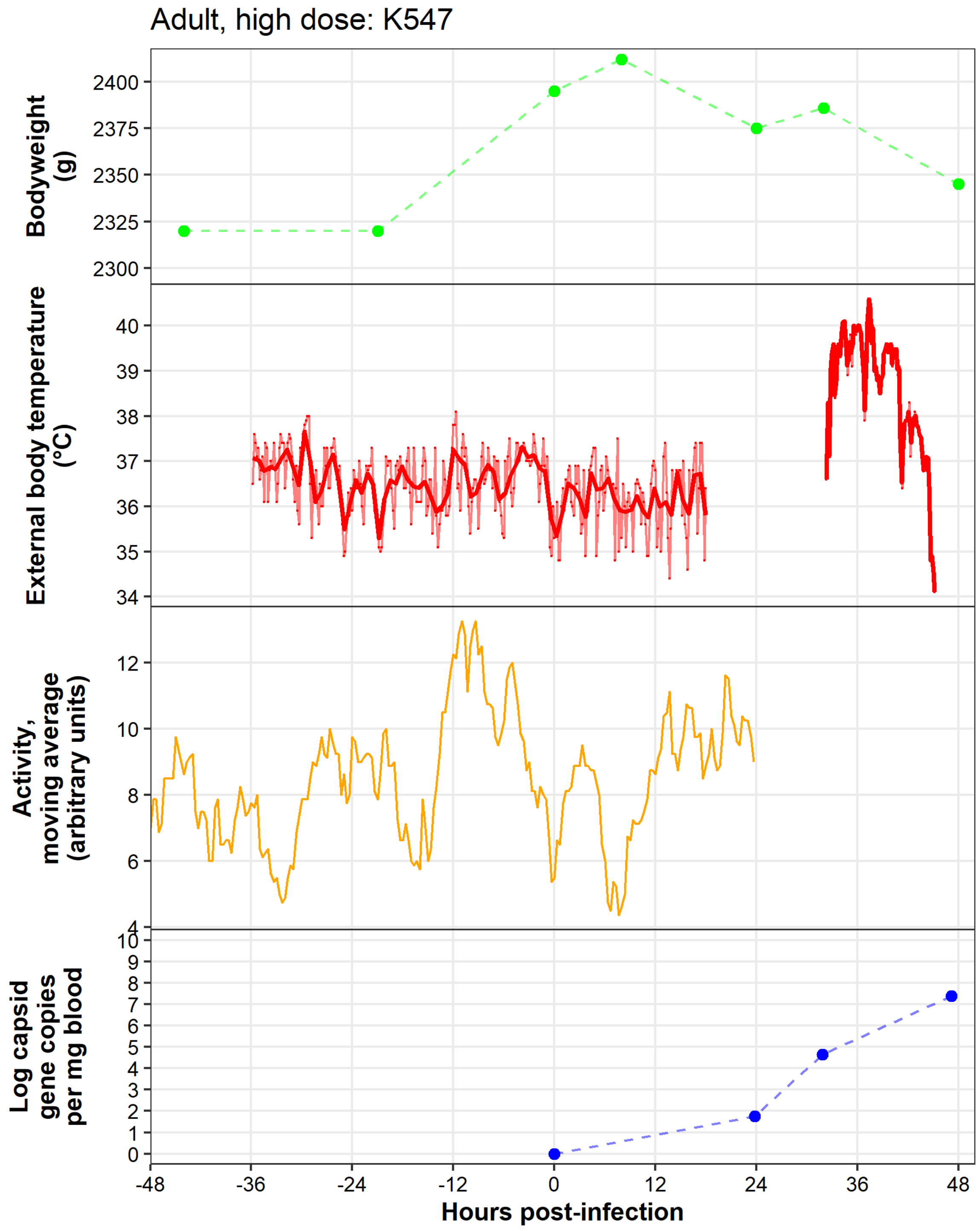

Caveats: Technical difficulties with temperature monitor prior to -36 hpi. Collar was twisted so that the temperature logger was not against skin from 18–32 hpi. Technical difficulties with activity monitor after 24 hpi.

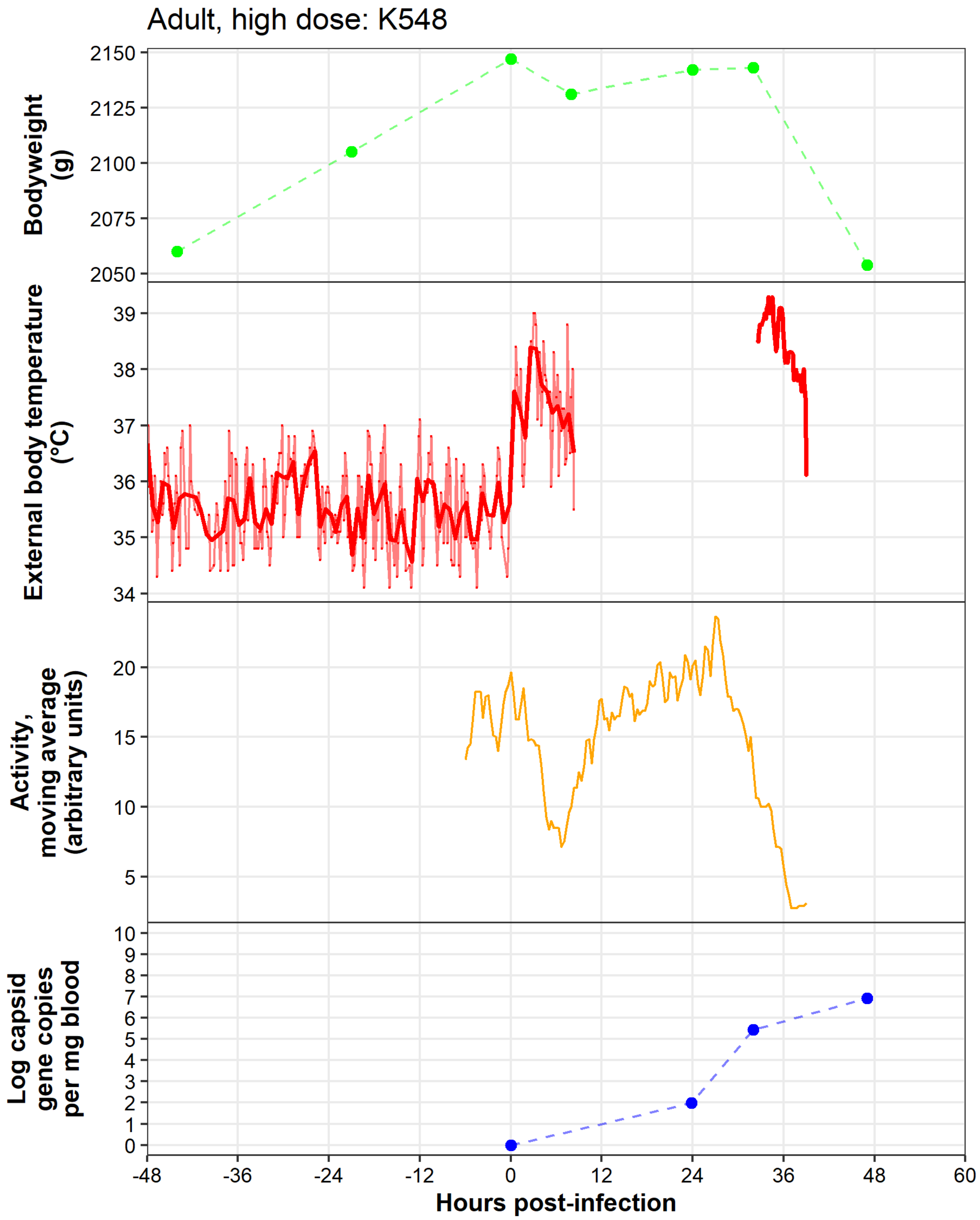

Caveats: Temperature monitor fell out of pouch around 10 hpi, replaced at 32 hpi.

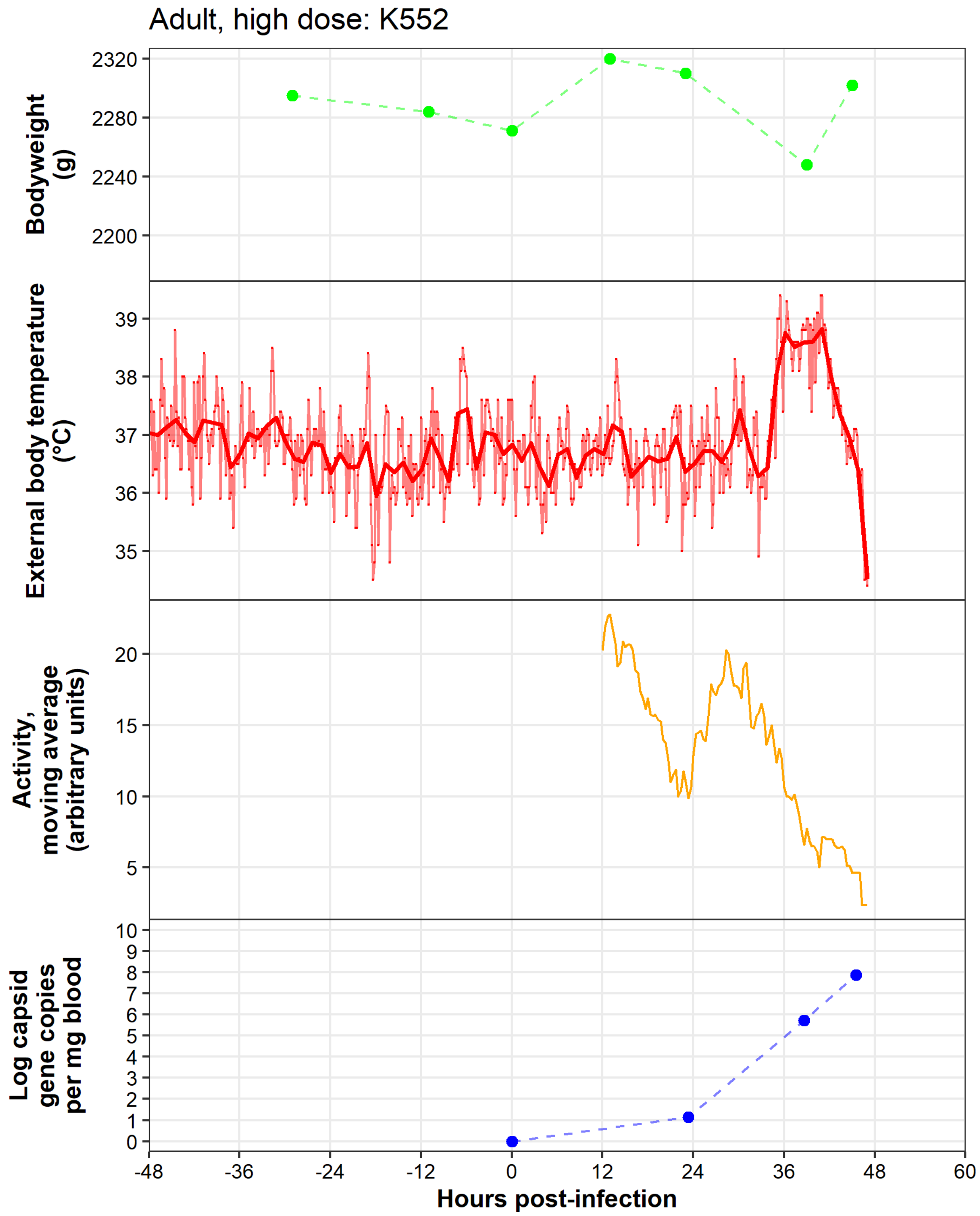

Caveats: Technical difficulties with activity monitor prior to 12 hpi.

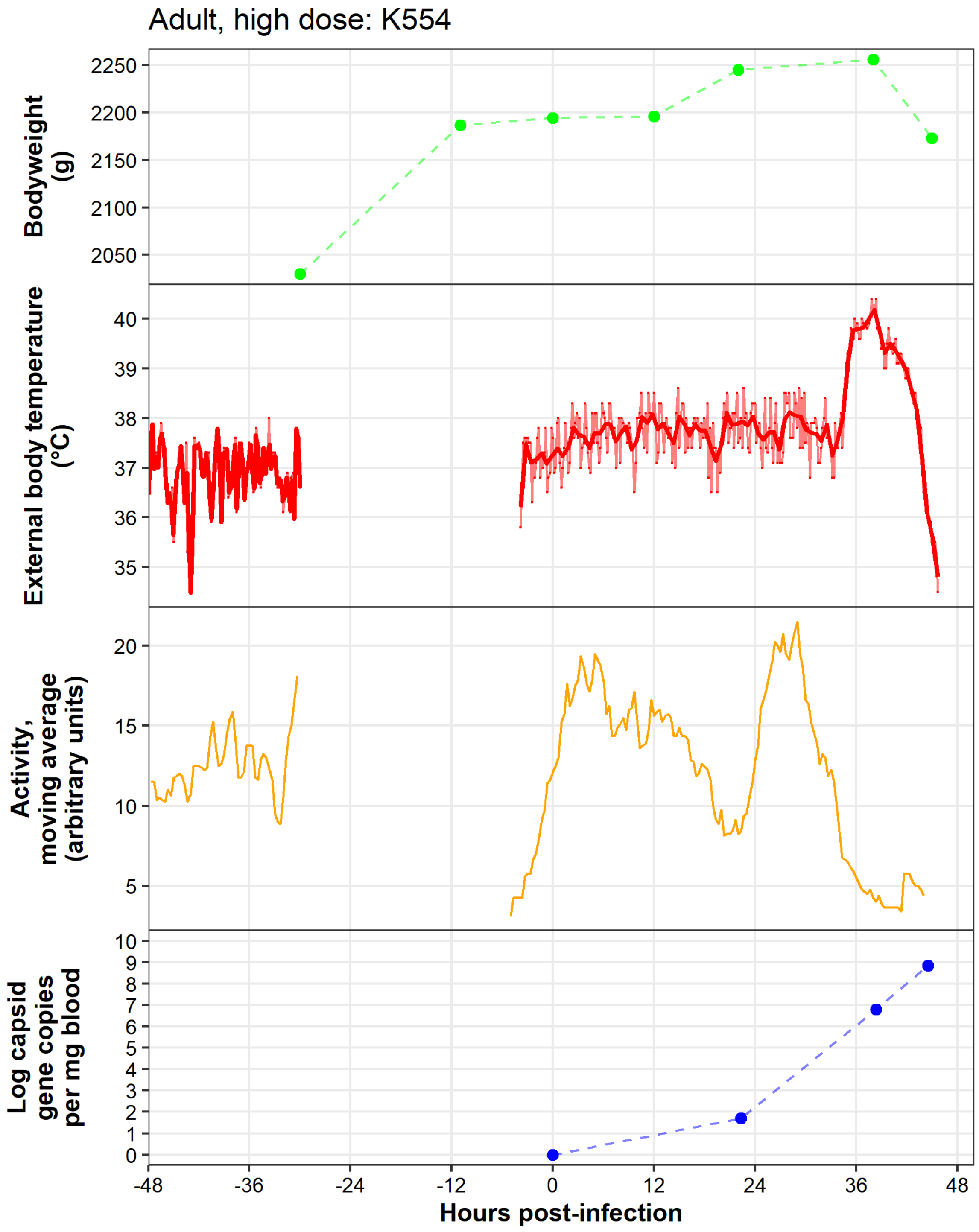

Caveats: Collar came off between -30 – -5 hpi.

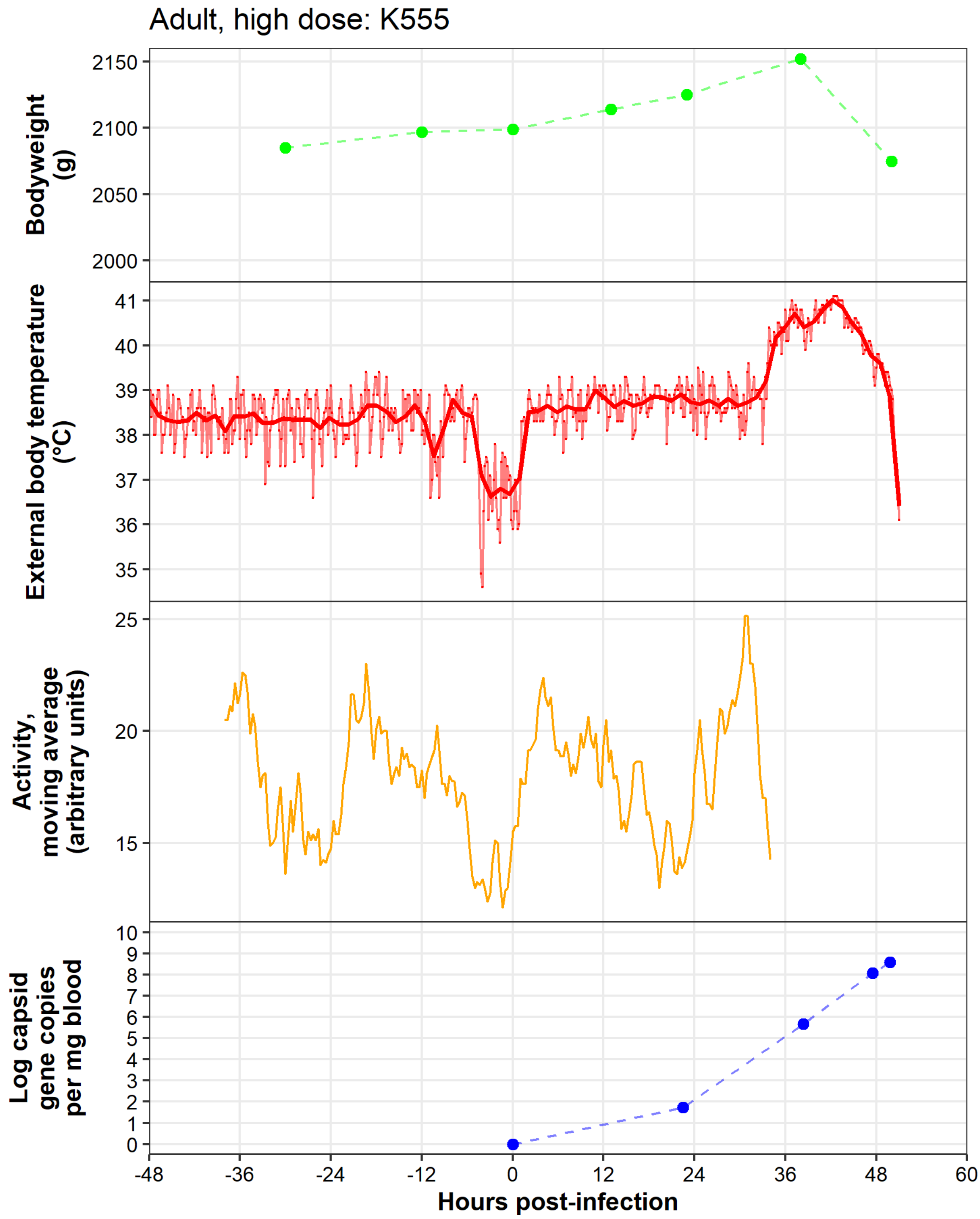

Caveats: Technical difficulties with activity monitor after 34 hpi. Temperature monitor replaced at -6 hpi and tightened at 0 hpi.

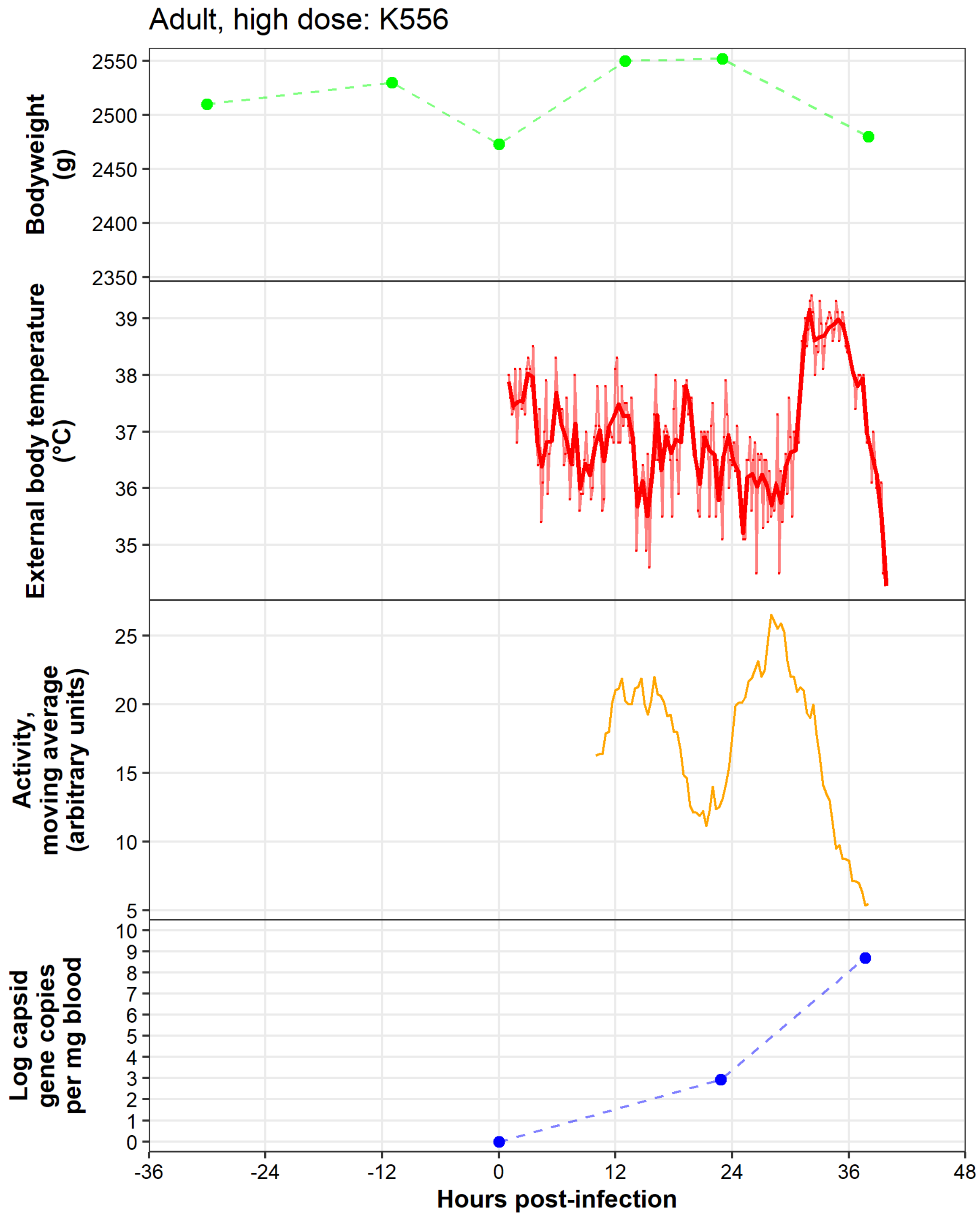

Caveats: Technical difficulties with activity monitor prior to 10 hpi and with temperature monitor prior to infection.

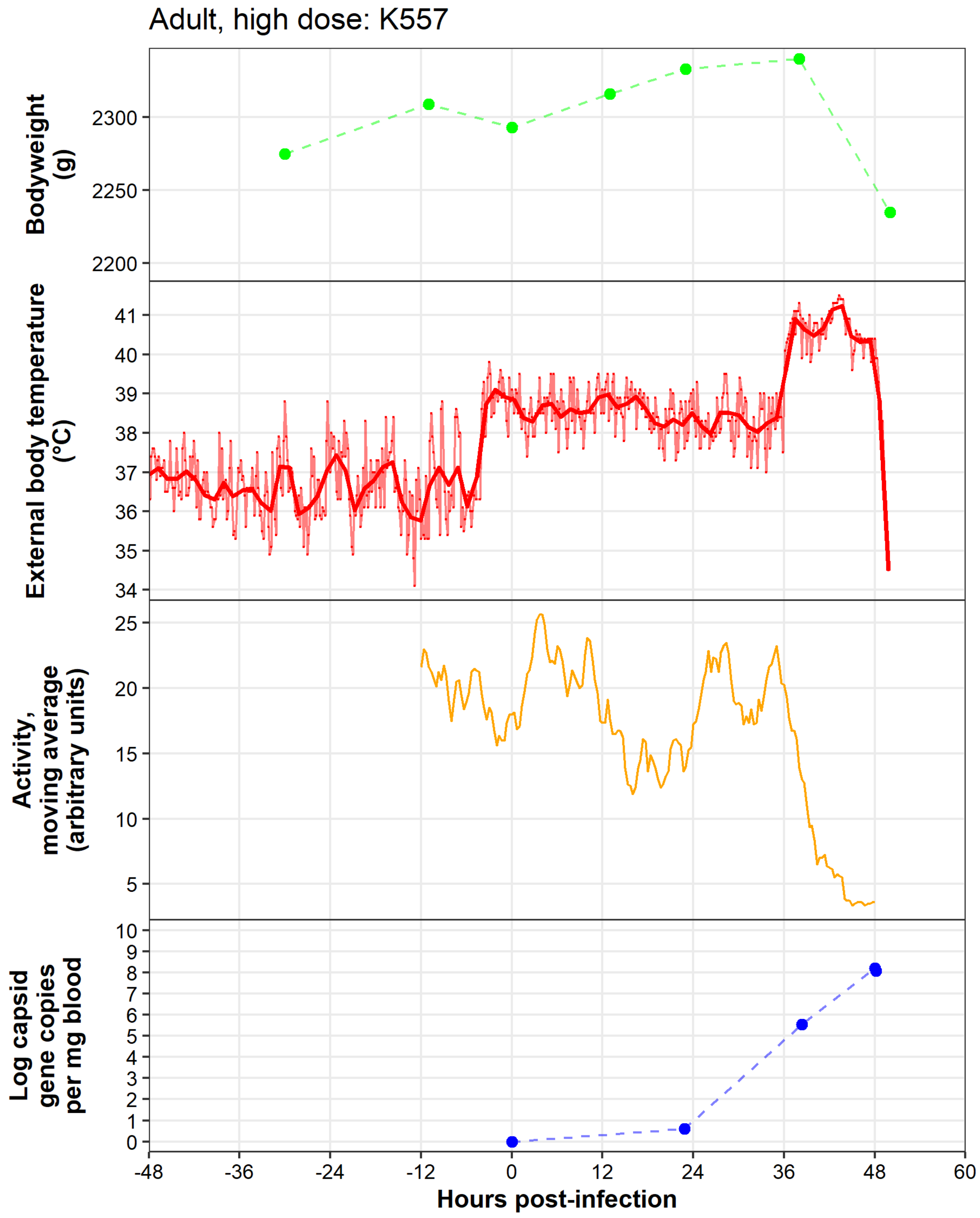

Caveats: Technical difficulties with activity monitor prior to -12 hpi. Collar loose prior to -6 hpi.

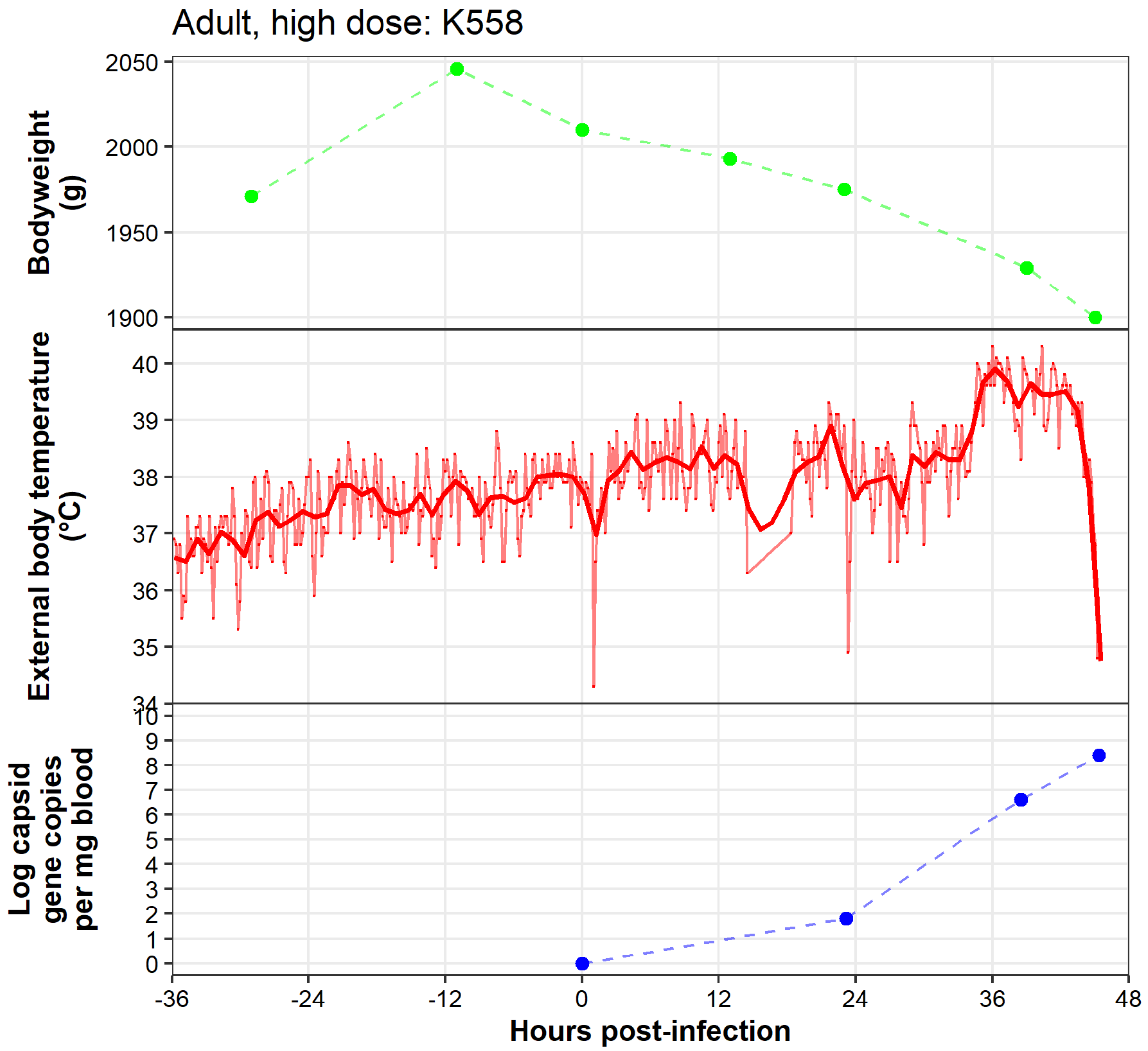

Caveats: Technical difficulties with activity monitor. Collar removed between 13–17 hpi.

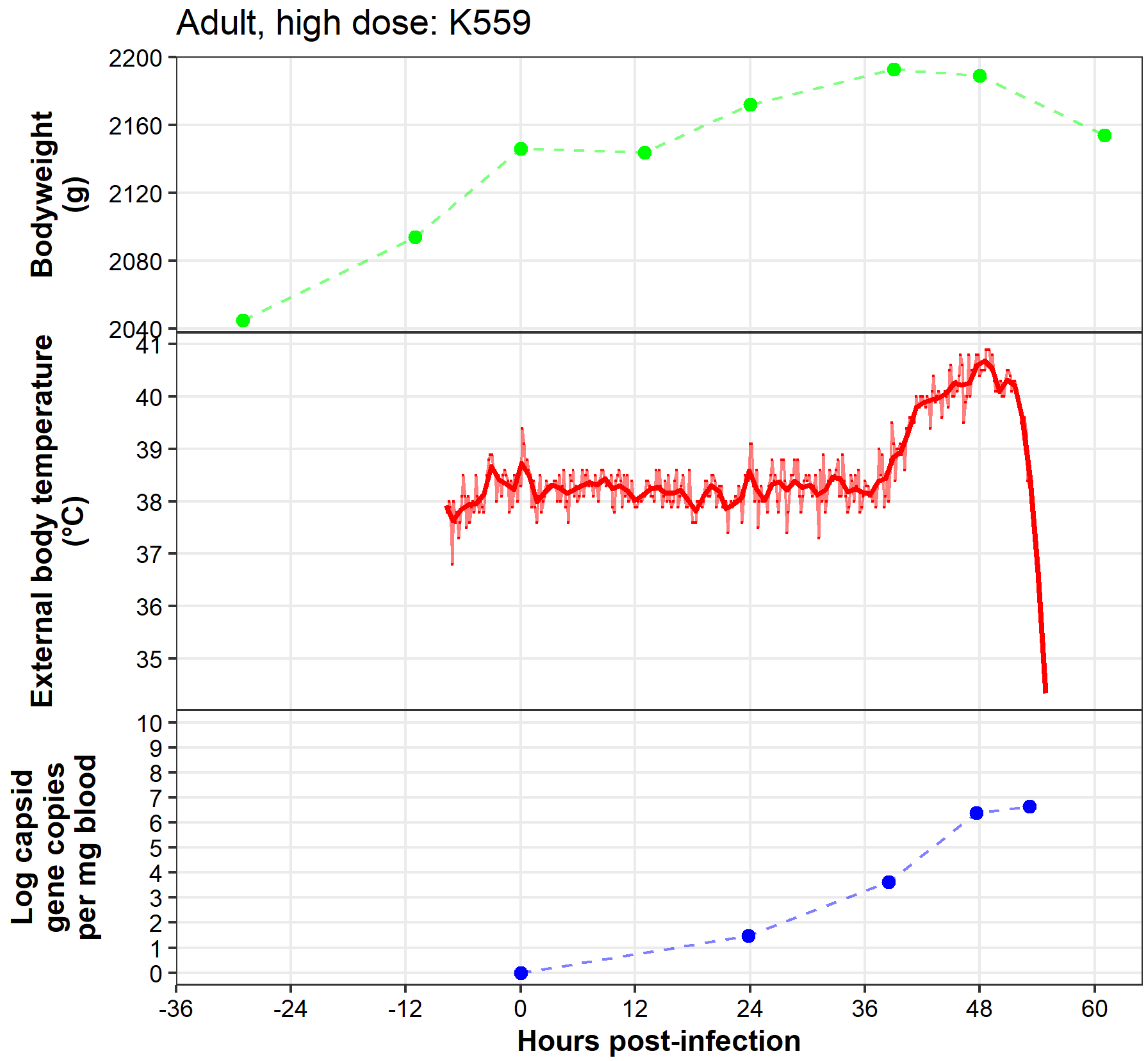

Caveats: Technical difficulties with activity monitor. Collar loose prior to -8 hpi.

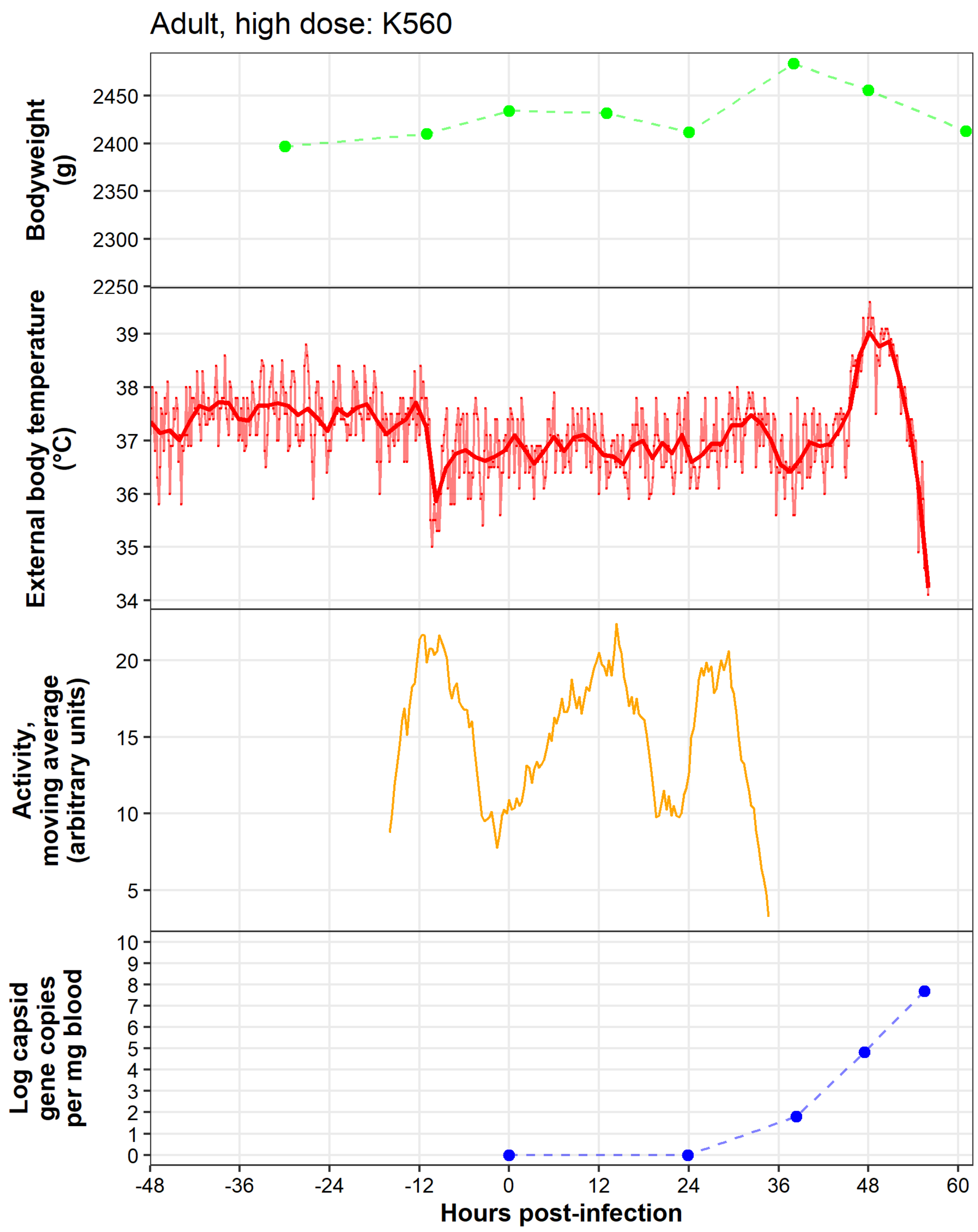

Caveats: Technical difficulties with activity monitor prior to -16 hpi and after 35 hpi.

Adult, low dose

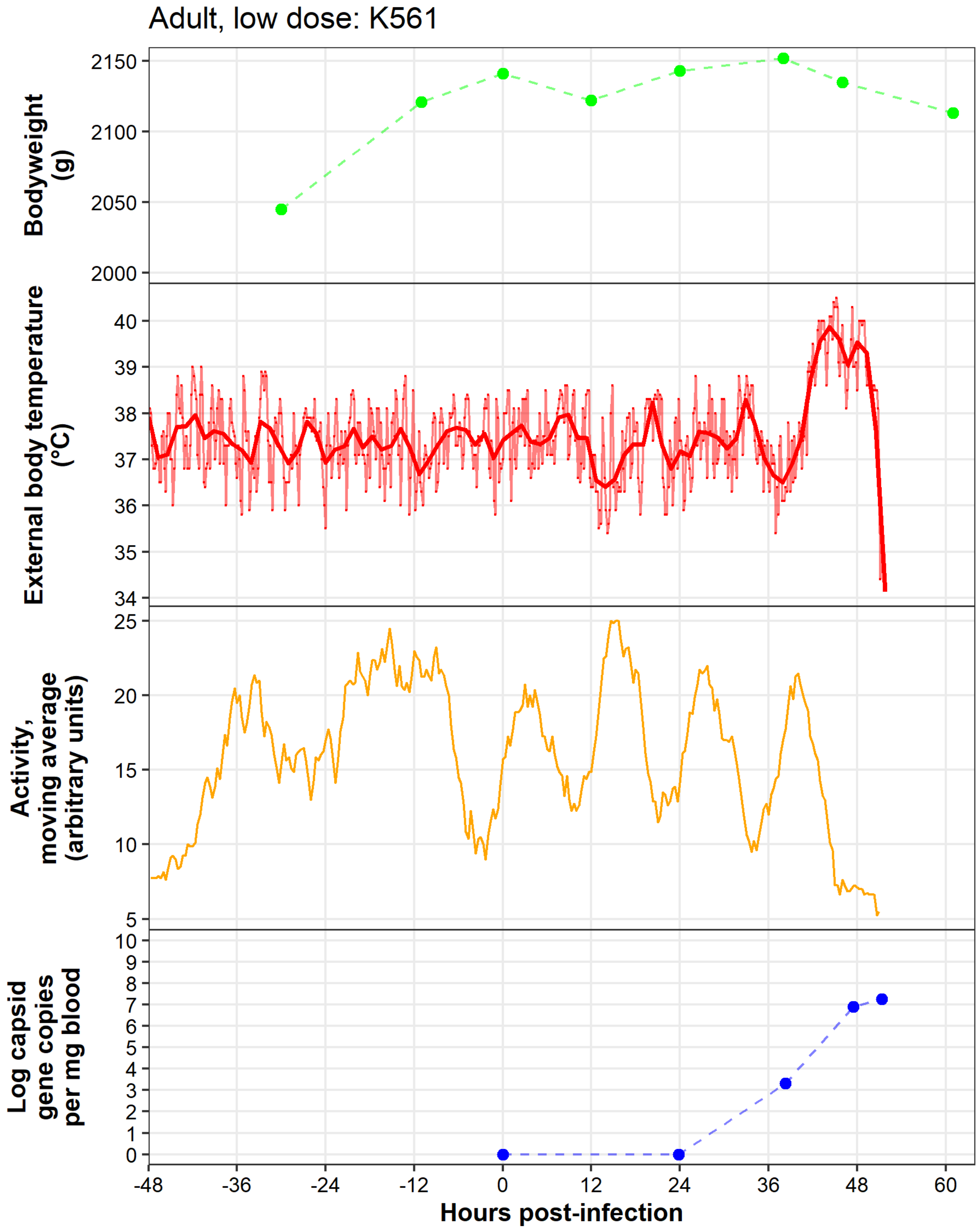

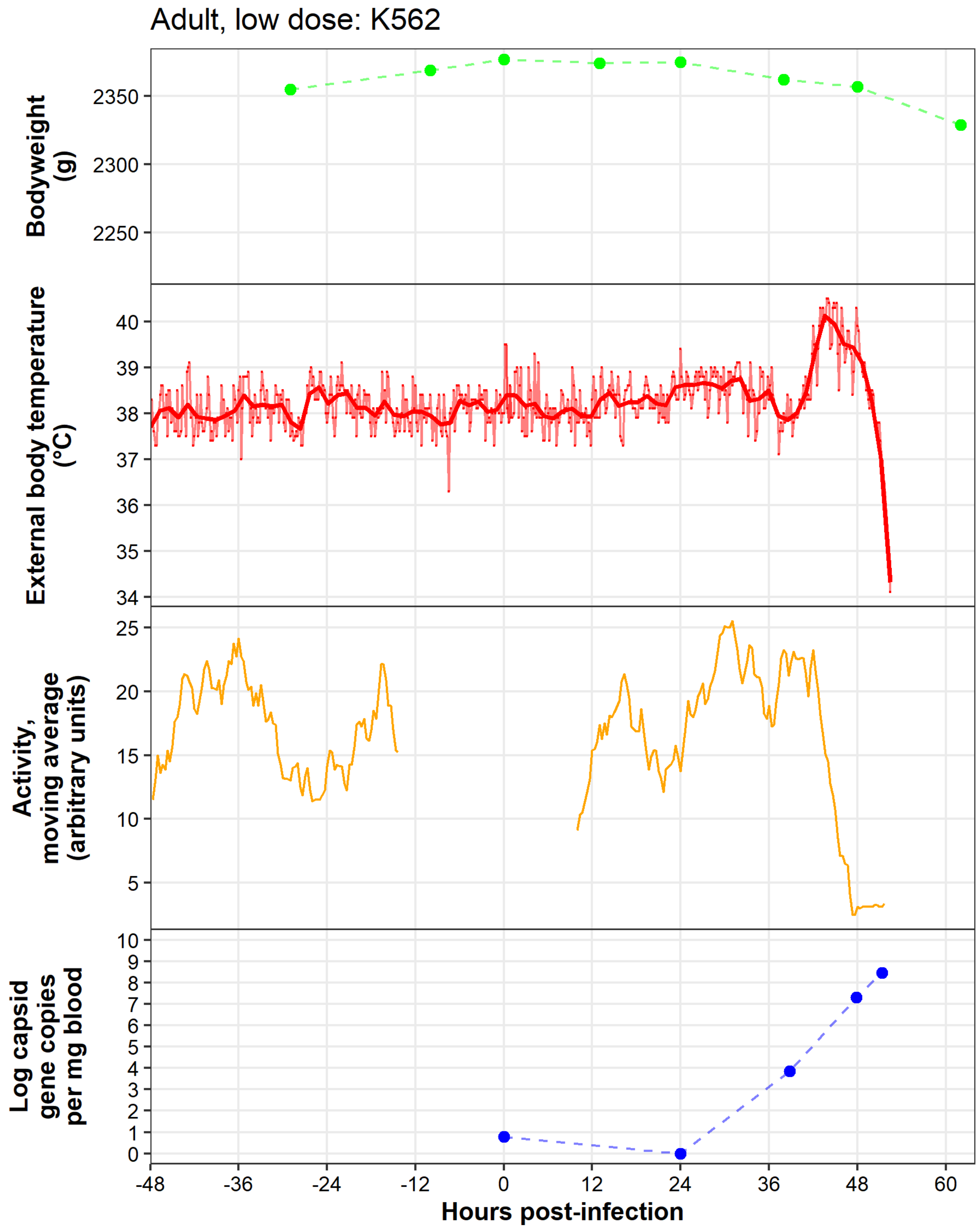

Caveats: Technical difficulties with activity monitor from -14–10 hpi.

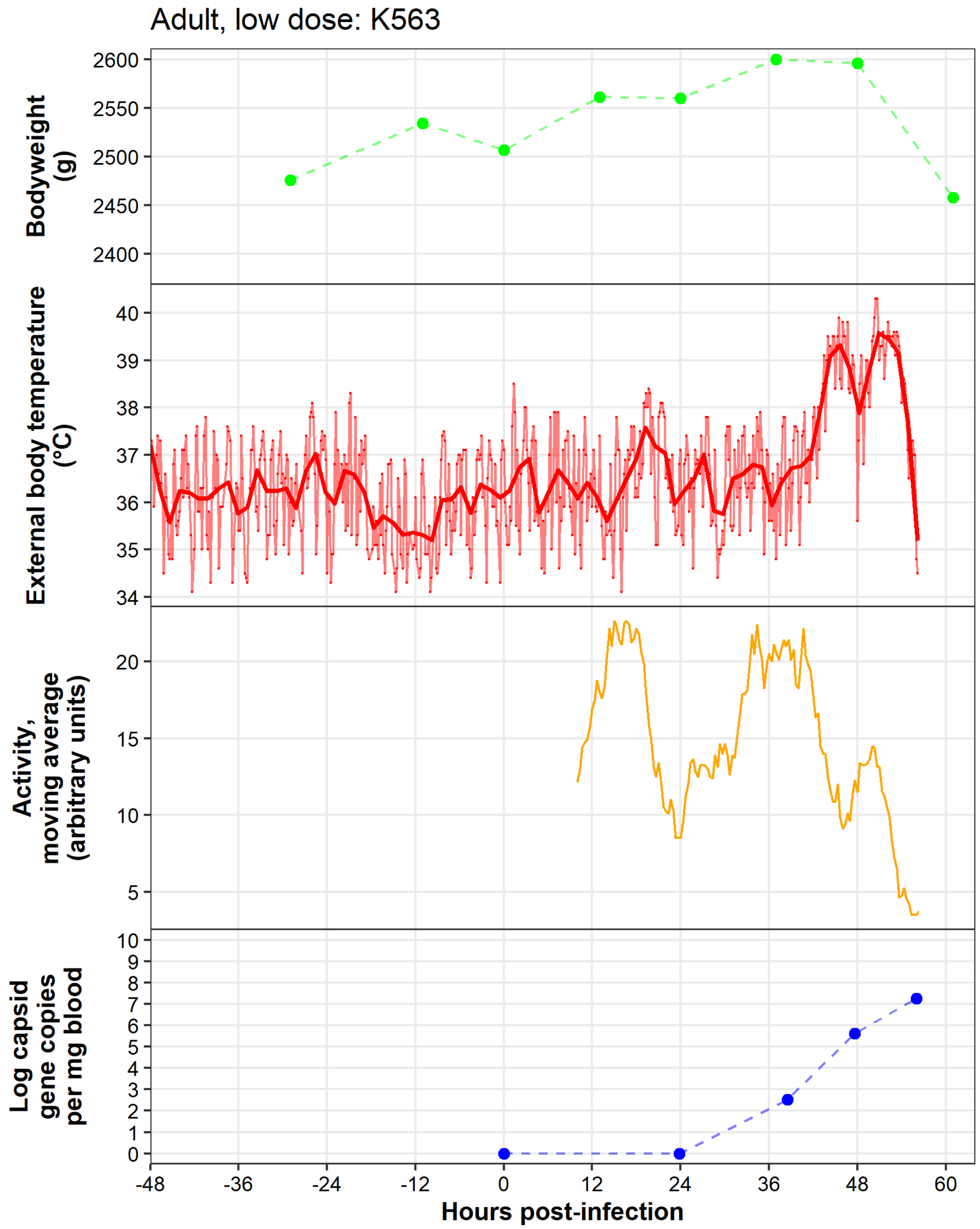

Caveats: Technical difficulties with activity monitor prior to -10 hpi.

Adult, low dose: K564

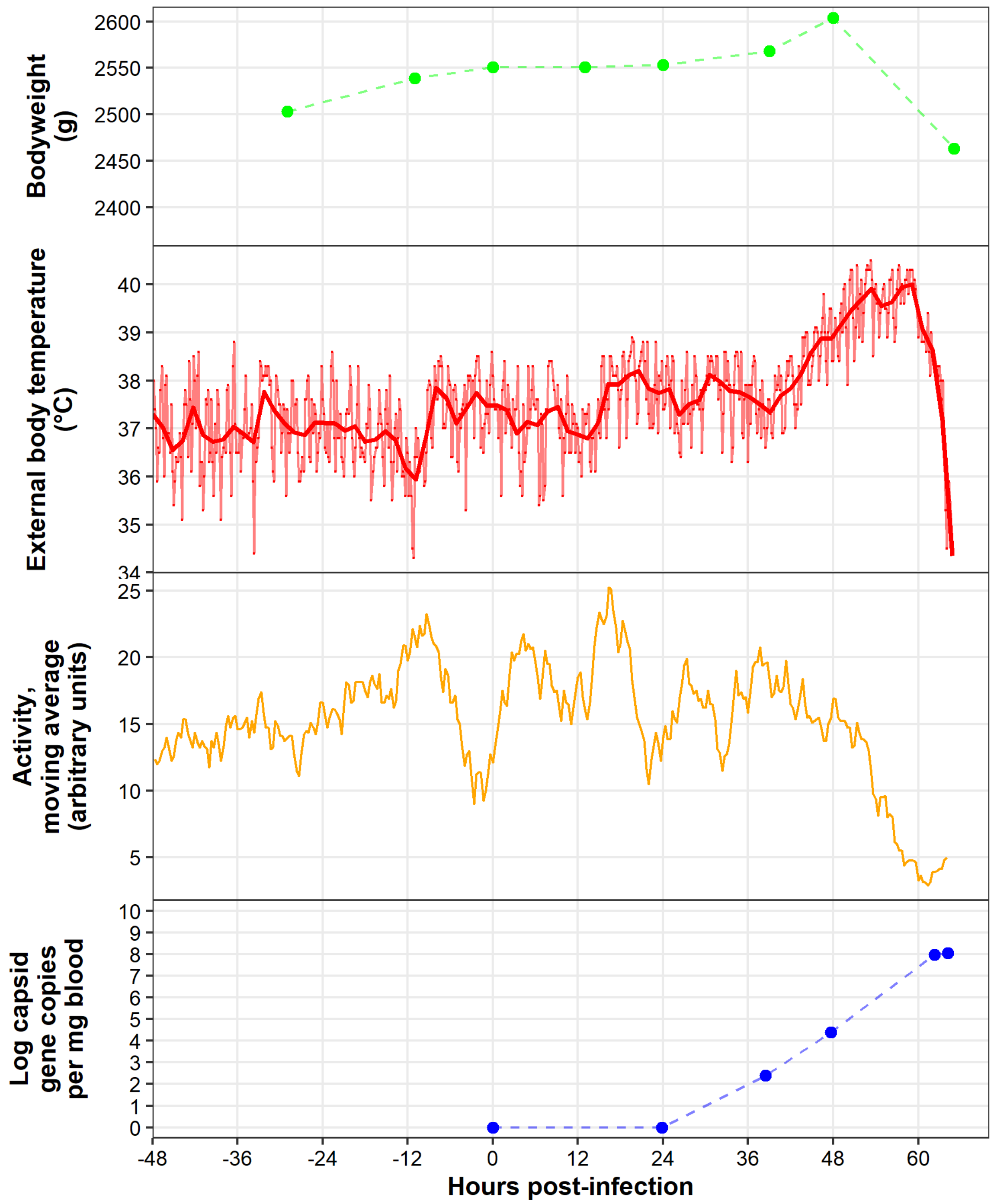

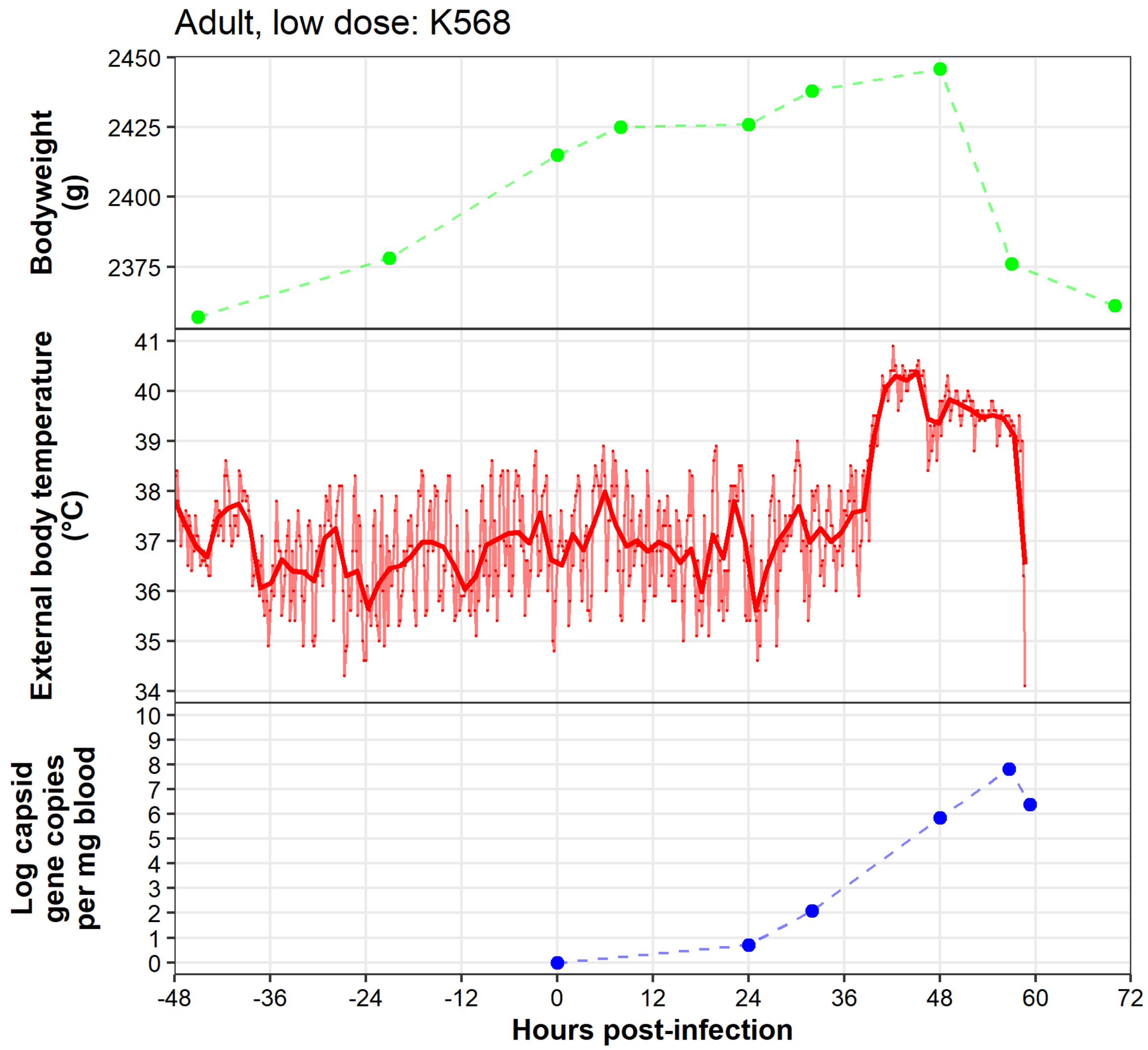

Caveats: Activity monitors were not used in trial 4 due to technical issues with the app update.

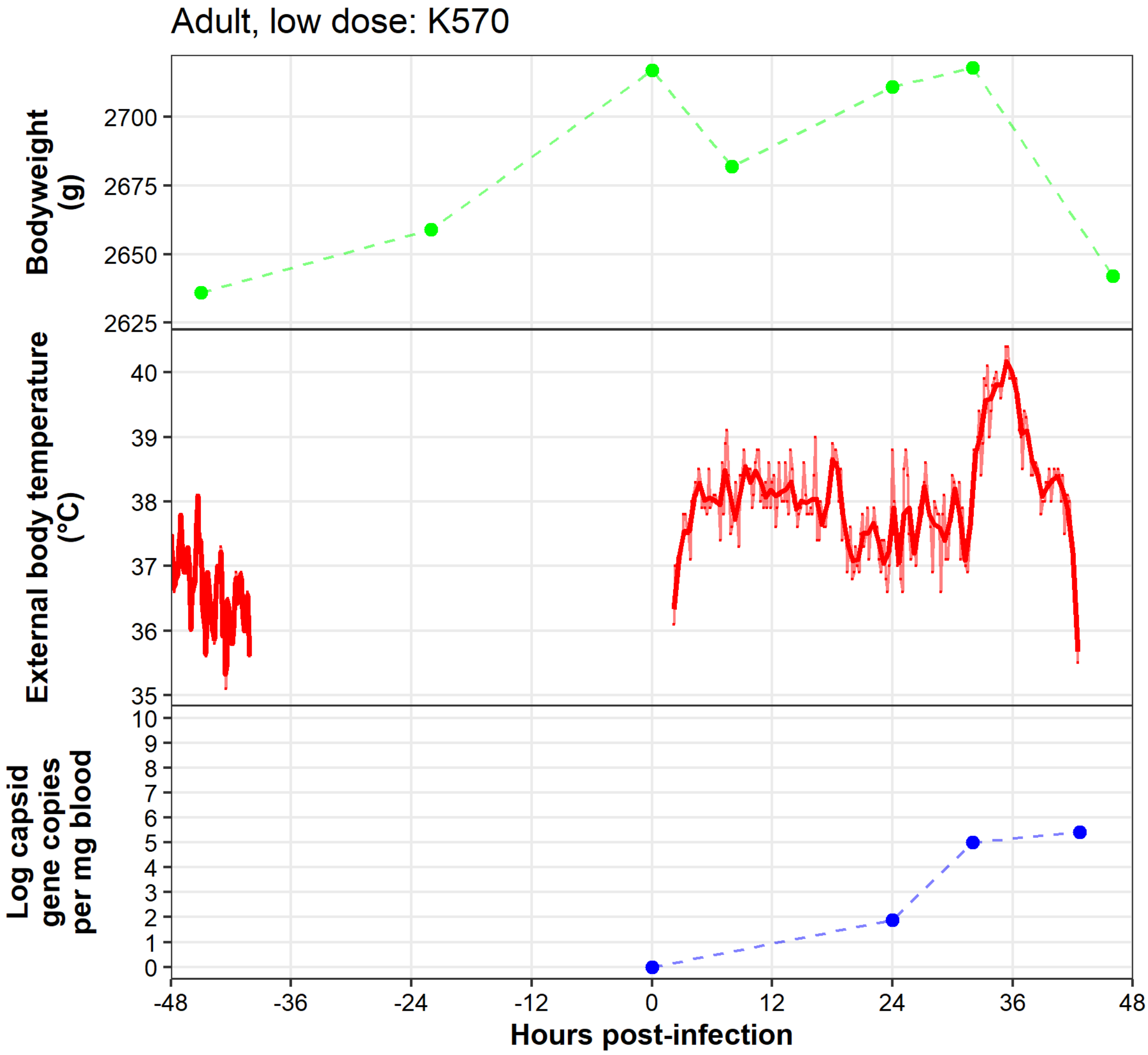

Caveats: Activity monitors were not used in trial 4 due to technical issues with the app update. Temperature monitor fell out of pouch around -40 hpi, replaced at 2 hpi.

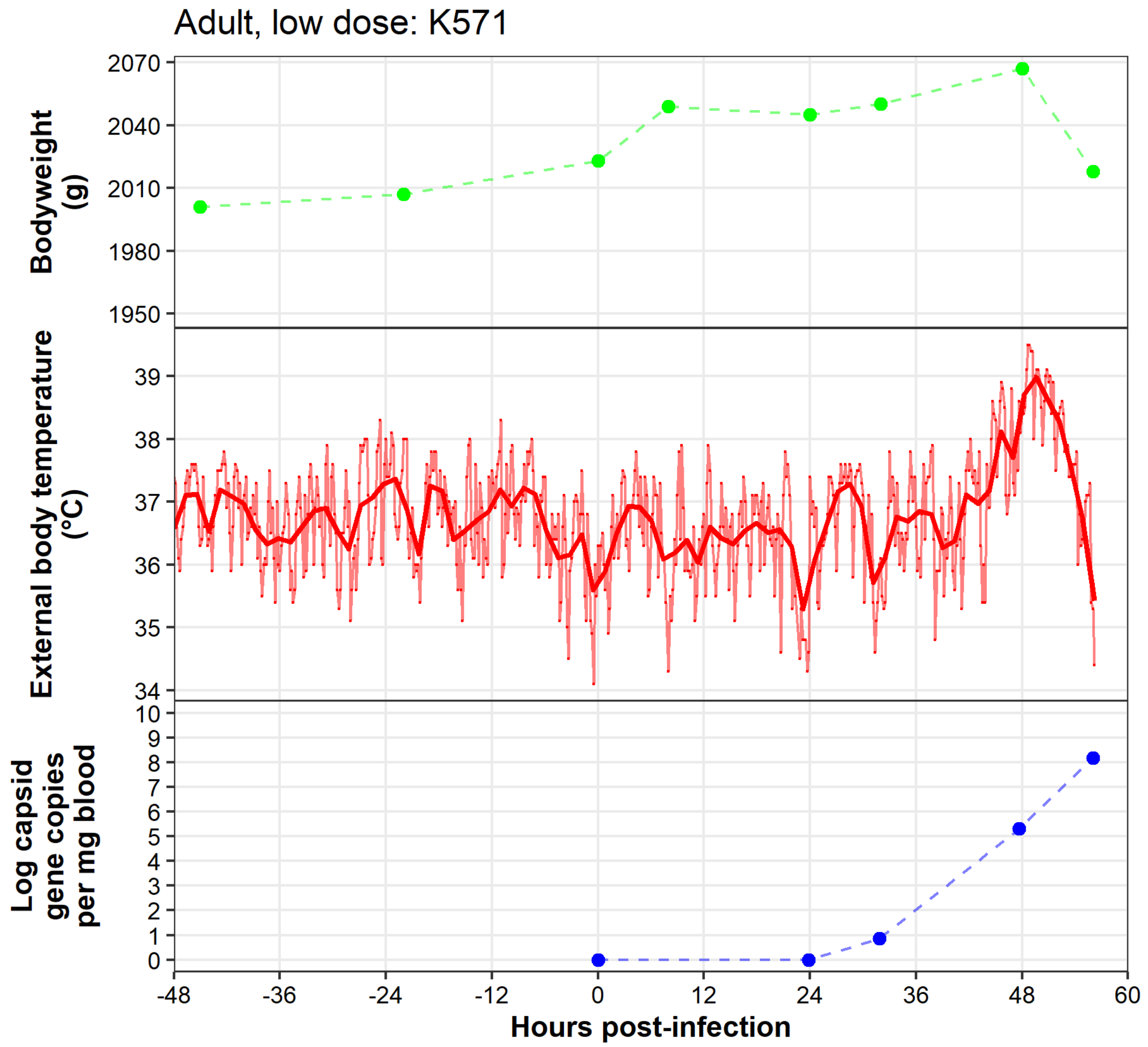

Caveats: Activity monitors were not used in trial 4 due to technical issues with the app update.

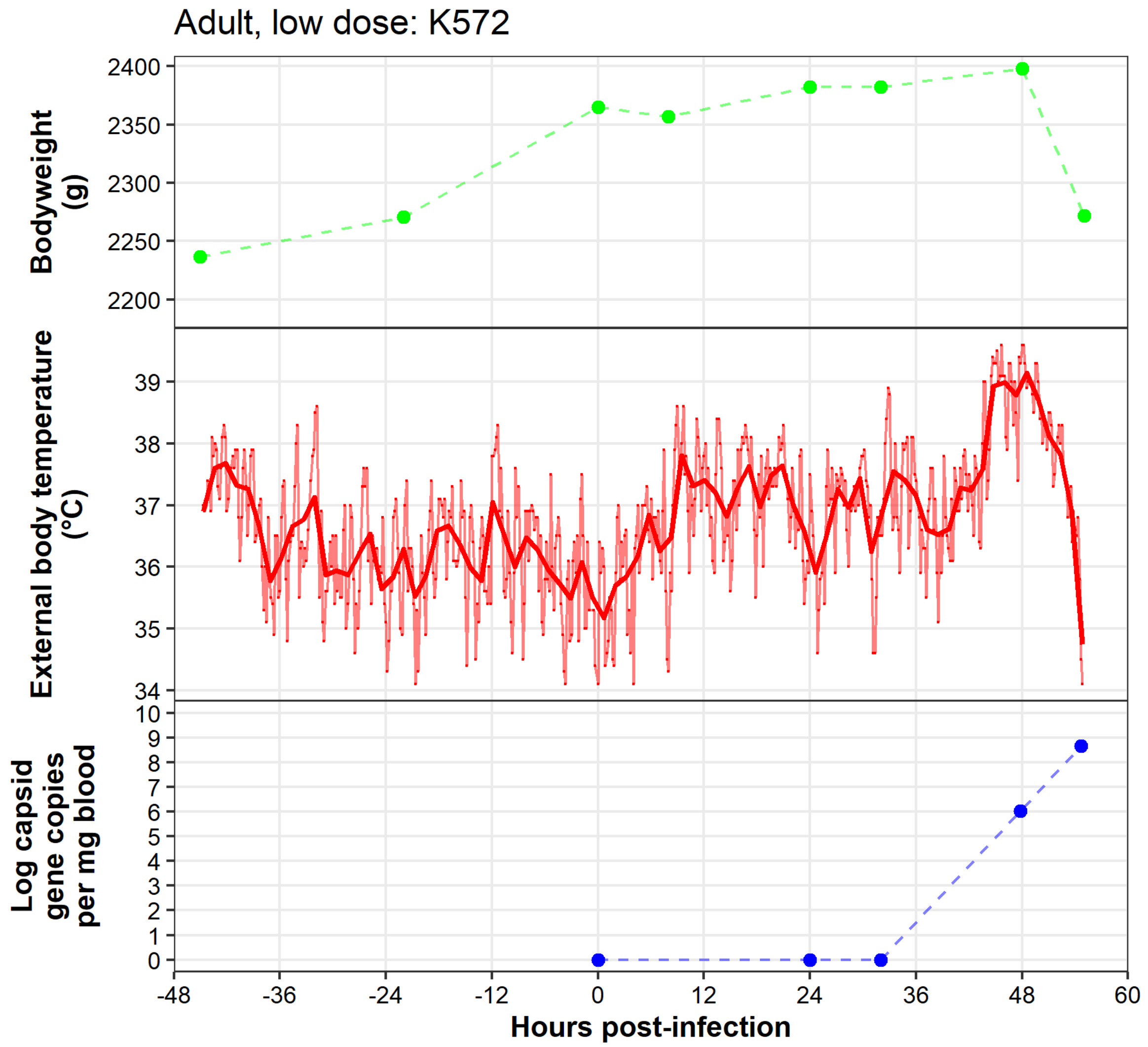

Caveats: Activity monitors were not used in trial 4 due to technical issues with the app update.

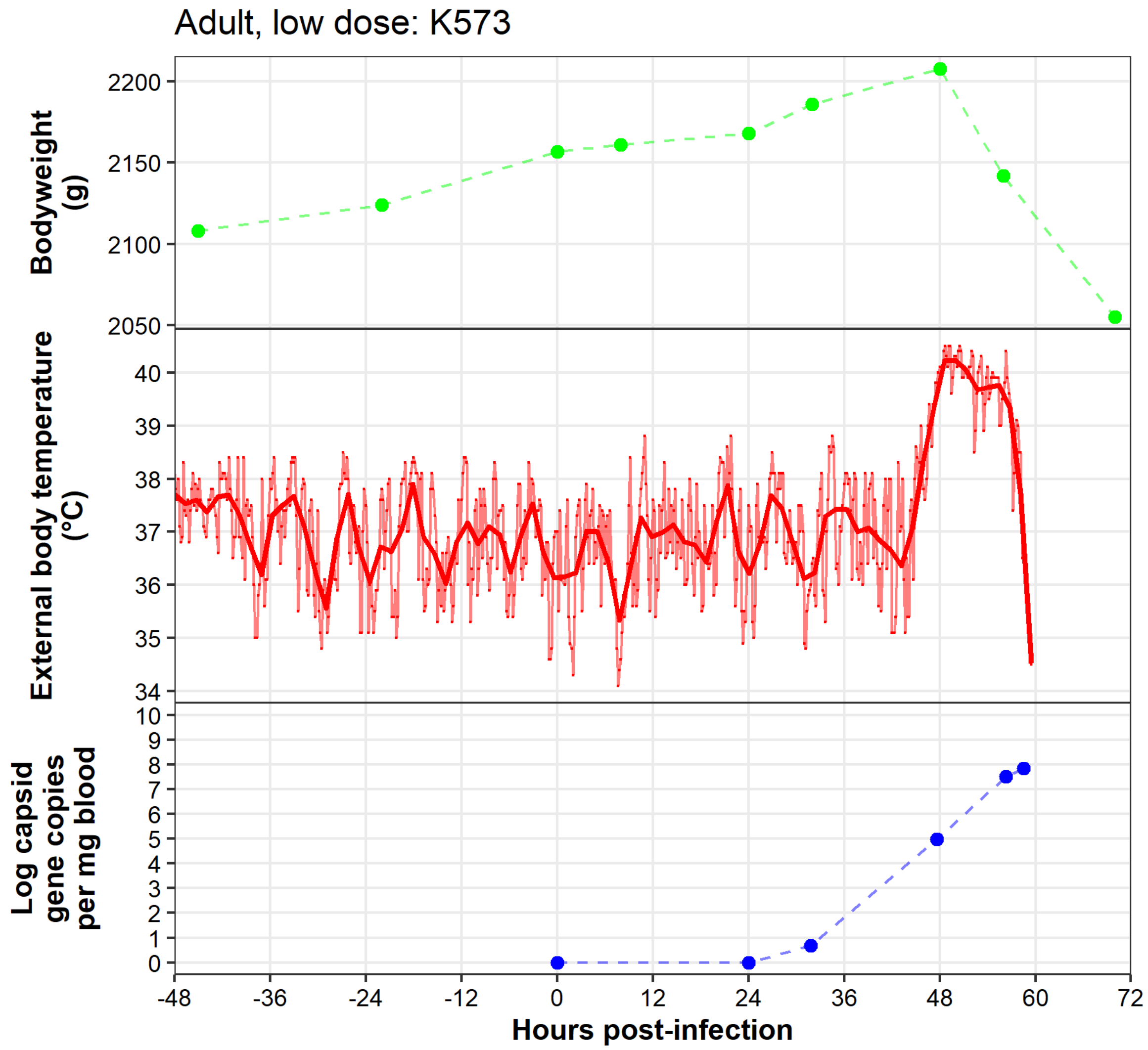

Caveats: Activity monitors were not used in trial 4 due to technical issues with the app update.

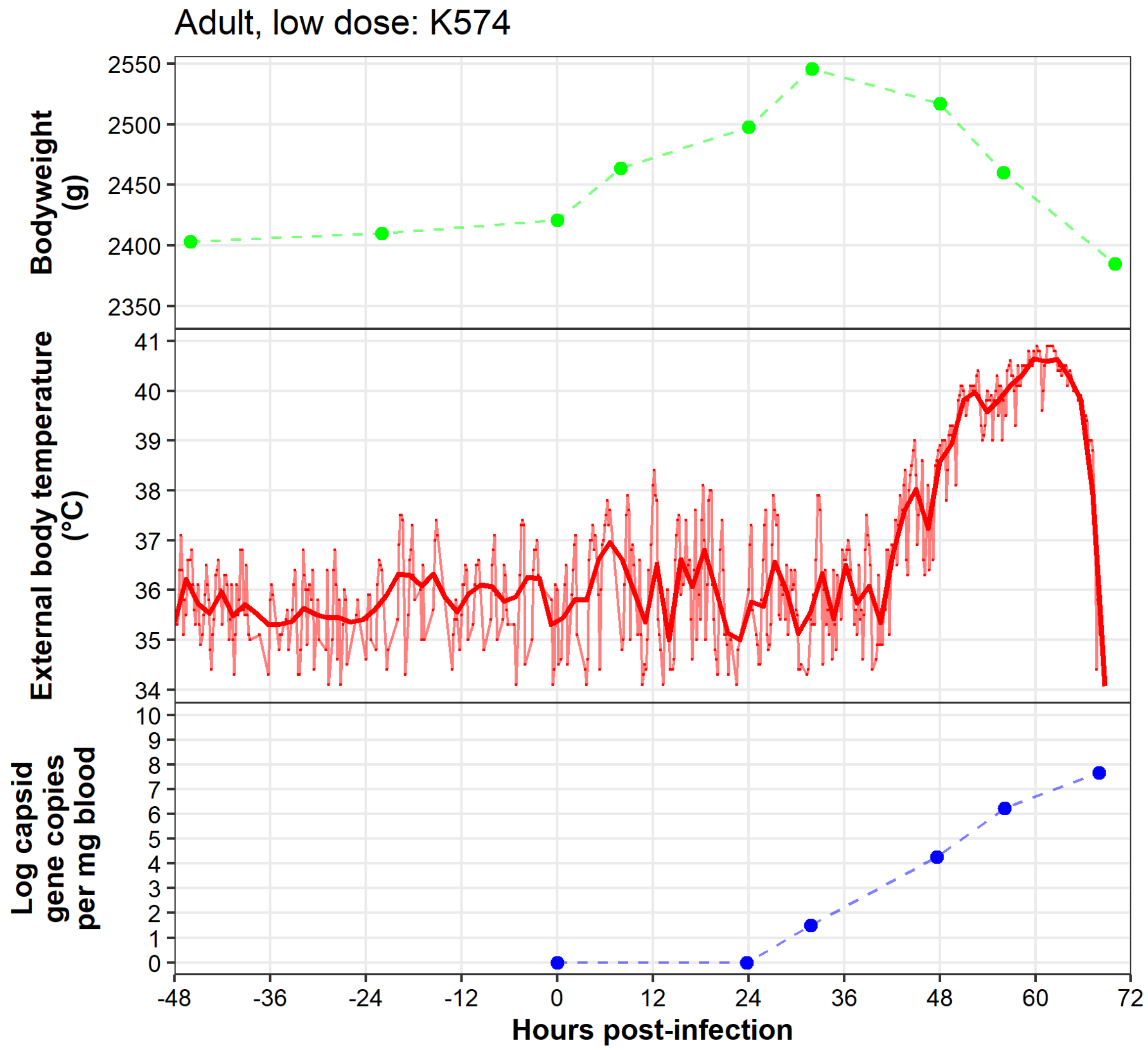

Caveats: Activity monitors were not used in trial 4 due to technical issues with the app update.

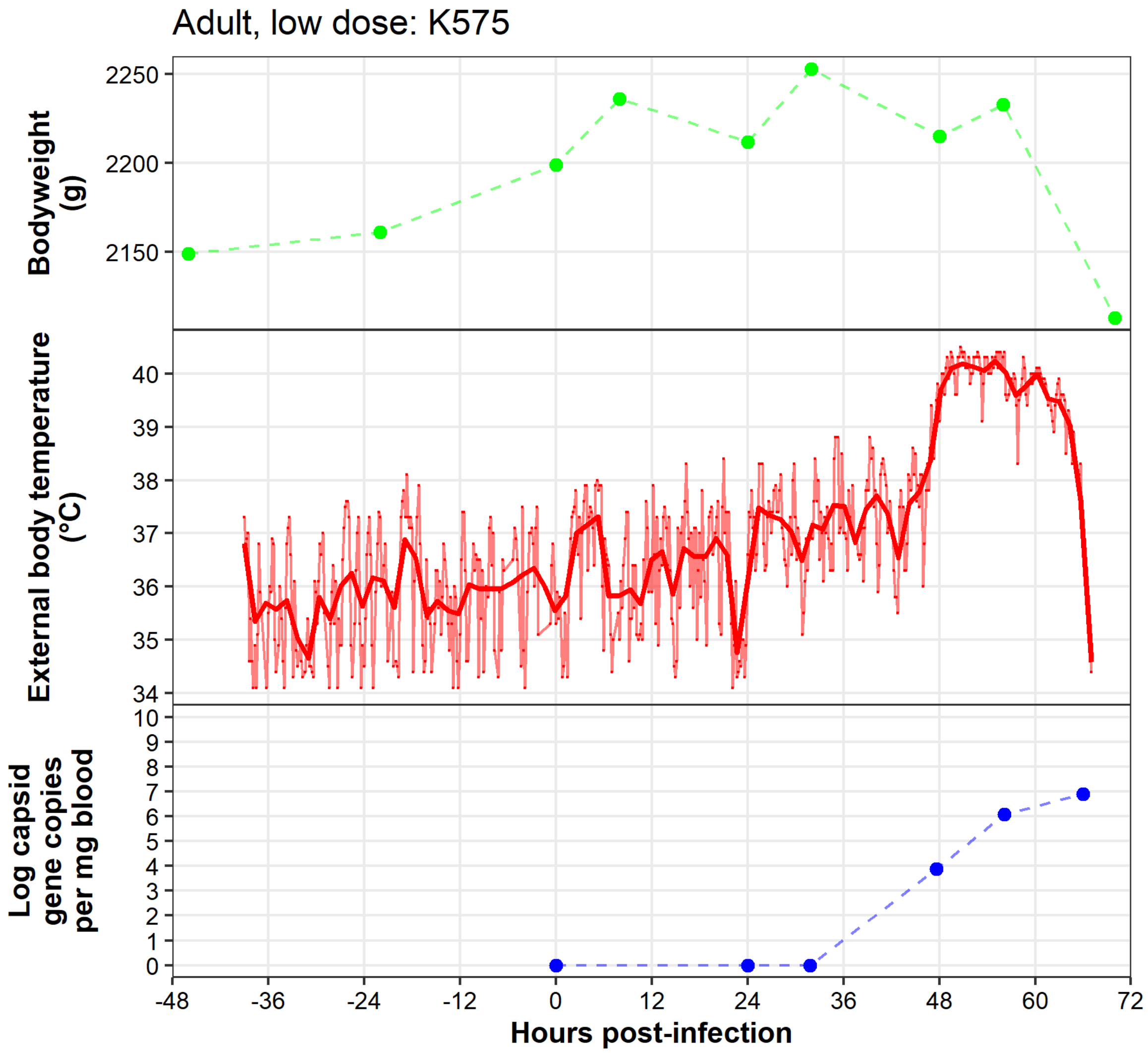

Caveats: Activity monitors were not used in trial 4 due to technical issues with the app update. Technical difficulties with collar prior to -38 hpi.

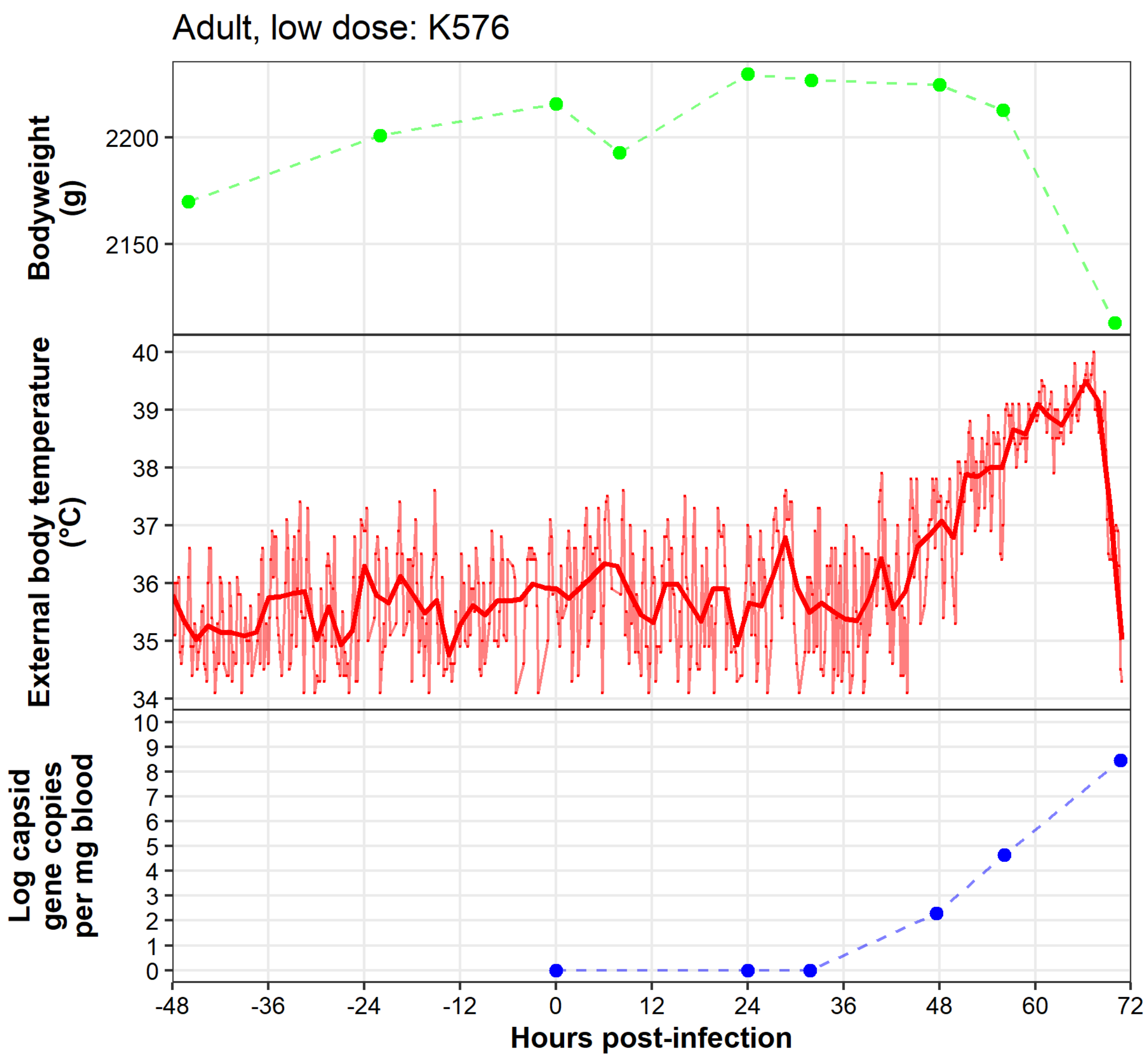

Caveats: Activity monitors were not used in trial 4 due to technical issues with the app update.

Kitten, control

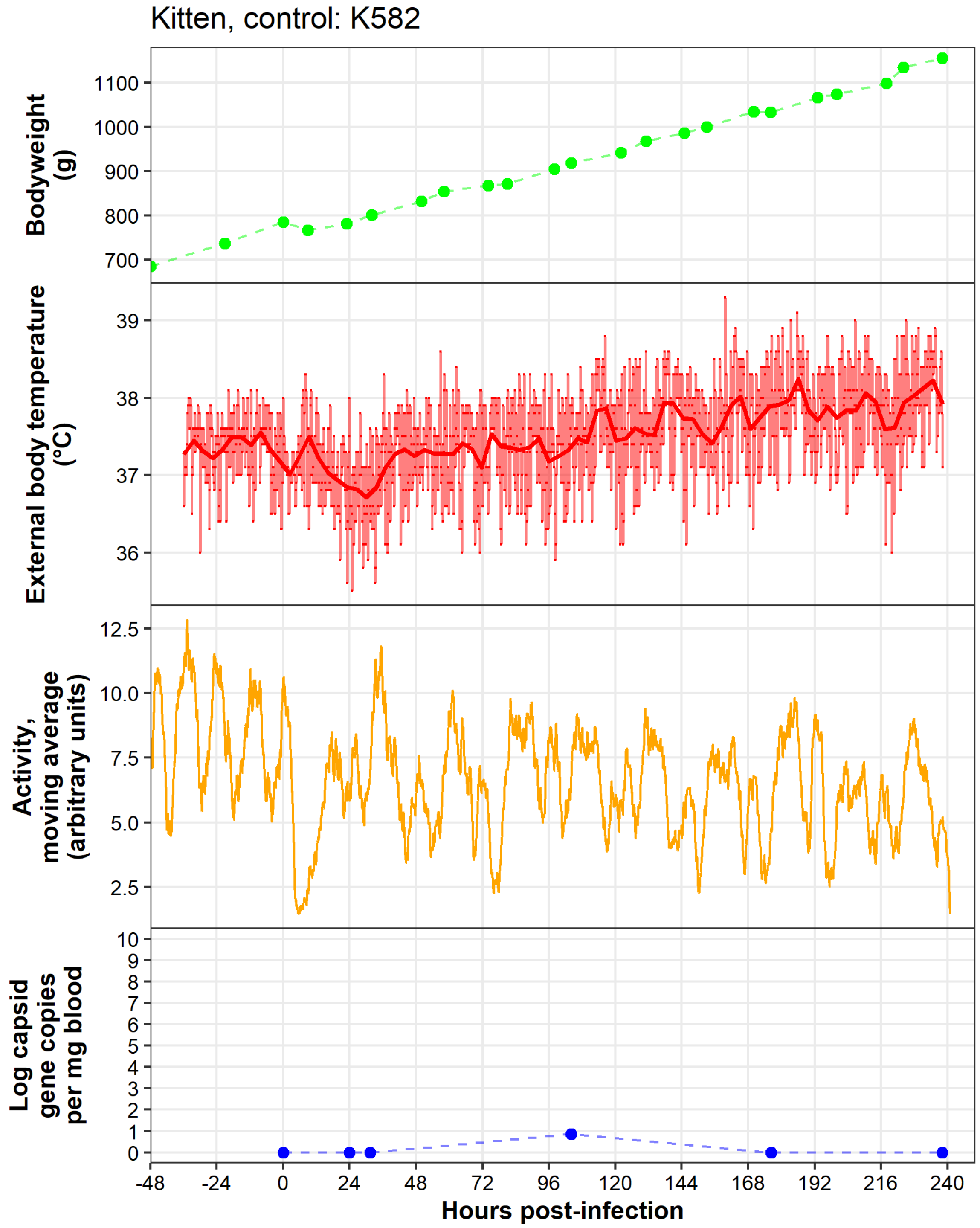

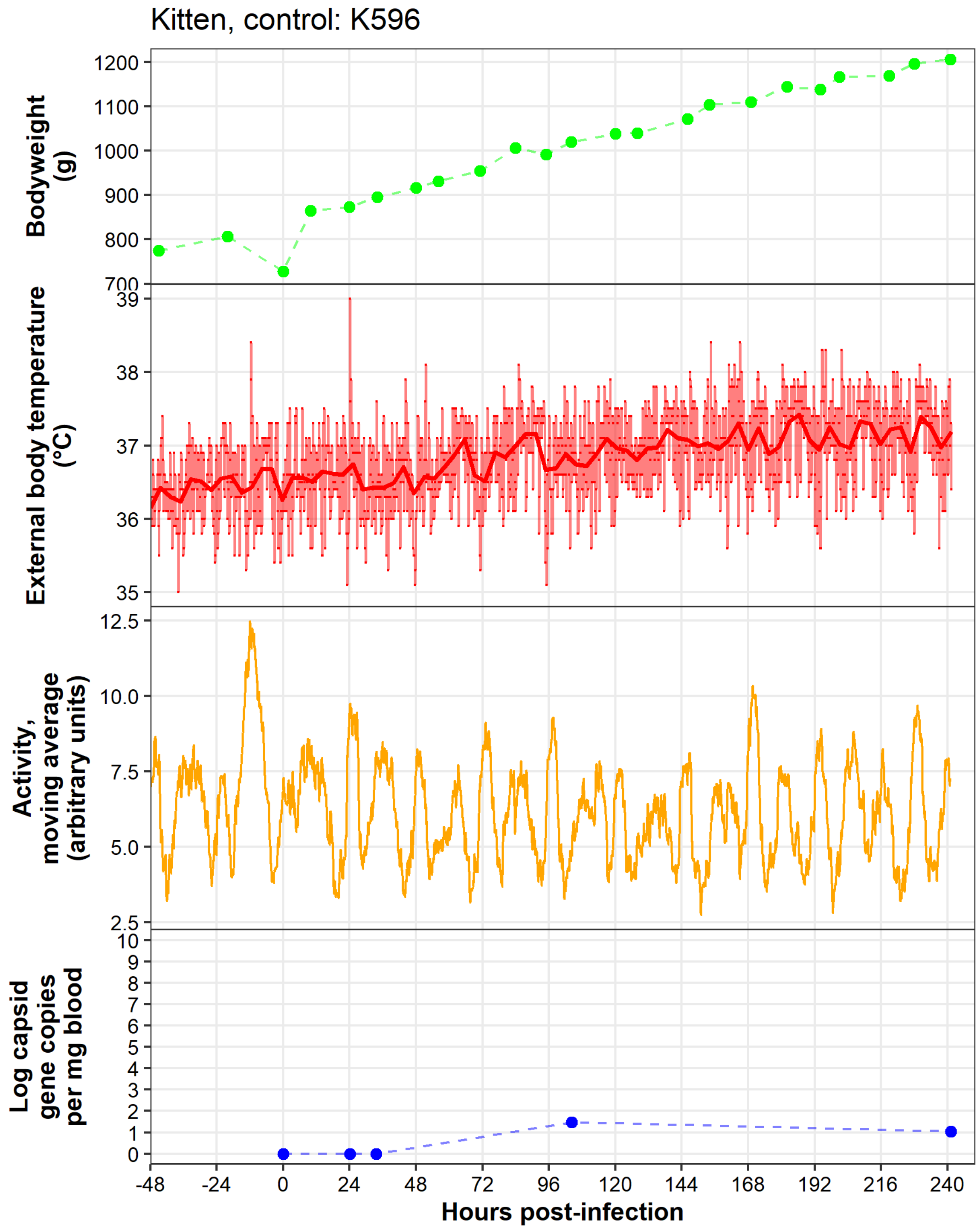

Kitten, control: K596
Bodyweight (g)
1200
1100
1000
900
800
700
External body temperature (°C)
39
38
37
36
35
Activity, moving average (arbitrary units)
12.5
10.0
7.5
5.0
2.5
Log capsid gene copies per mg blood
10
9
8
7
6
5
4
3
2
1
0
-48
-24
0
24
48
72
96
120
144
168
192
216
240
Hours post-infection

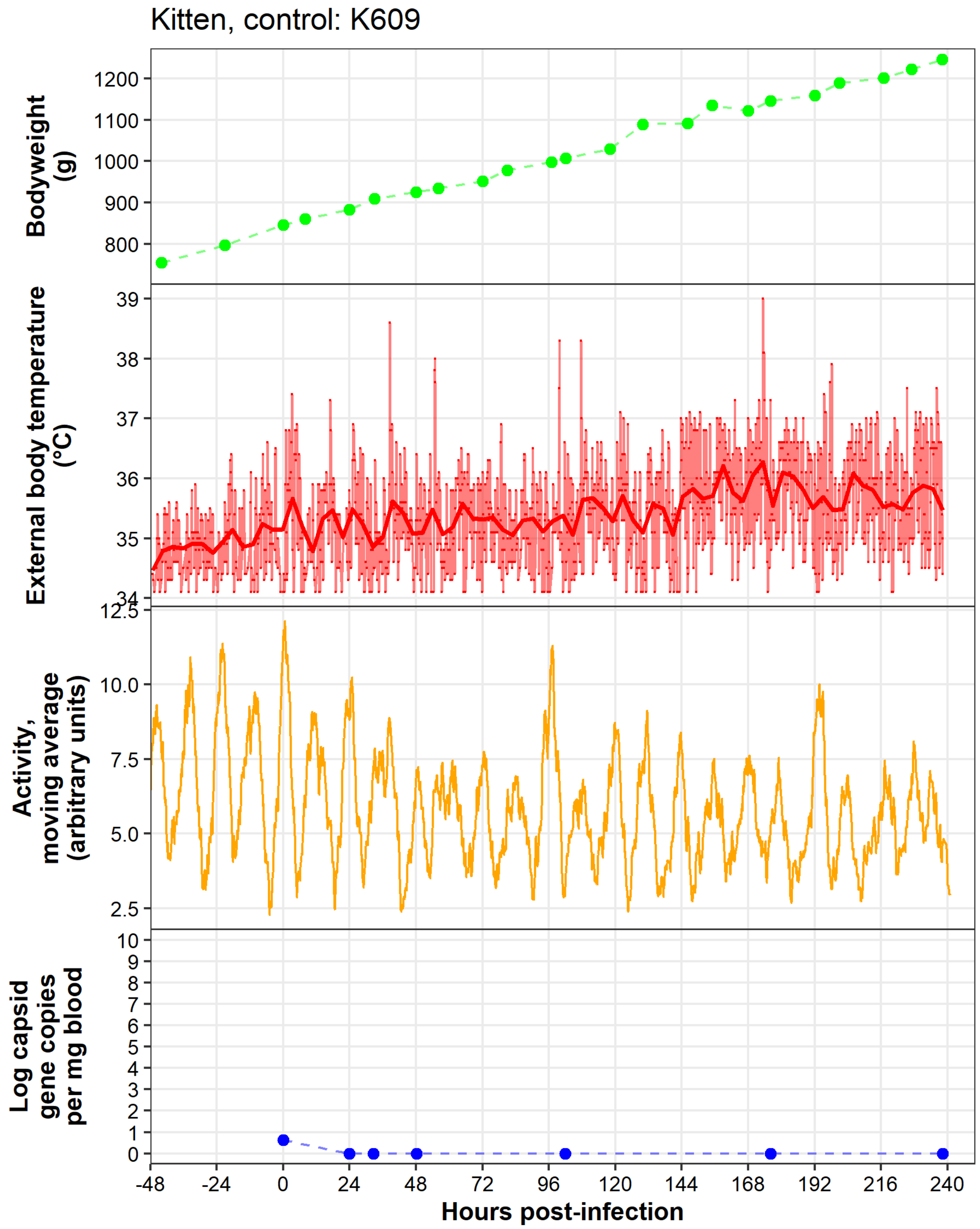
Kitten, control: K609
Bodyweight (g)
1200
1100
1000
900
800
External body temperature (°C)
39
38
37
36
35
34
Activity, moving average (arbitrary units)
12.5
10.0
7.5
5.0
2.5
Log capsid gene copies per mg blood
10
9
8
7
6
5
4
3
2
1
0
-48
-24
0
24
48
72
96
120
144
168
192
216
240
Hours post-infection

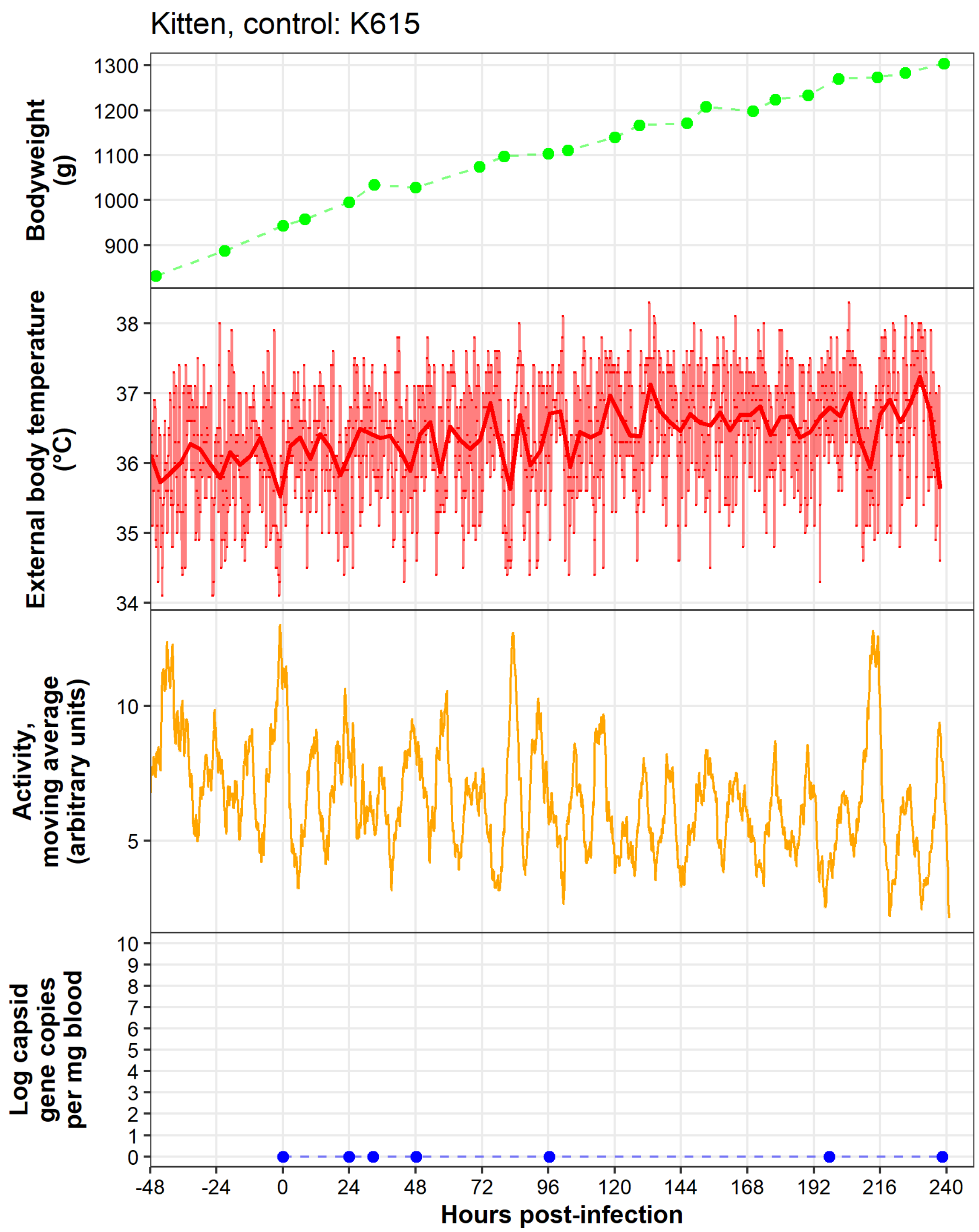
Kitten, control: K615
Bodyweight (g)
1300
1200
1100
1000
900
External body temperature (°C)
38
37
36
35
34
Activity, moving average (arbitrary units)
10
5
Log capsid gene copies per mg blood
10
9
8
7
6
5
4
3
2
1
0
-48
-24
0
24
48
72
96
120
144
168
192
216
240
Hours post-infection

<u>Kitten, high dose</u>

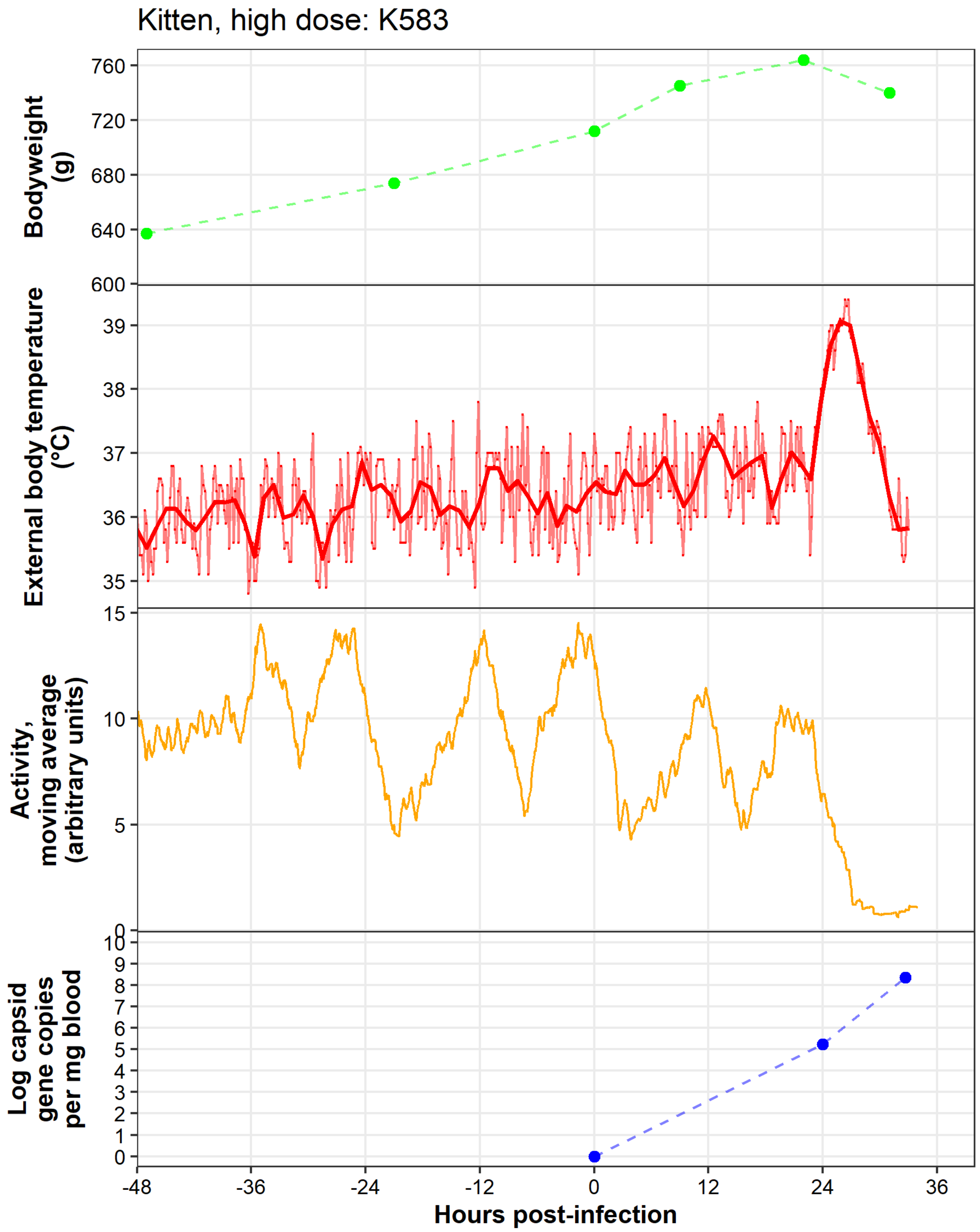

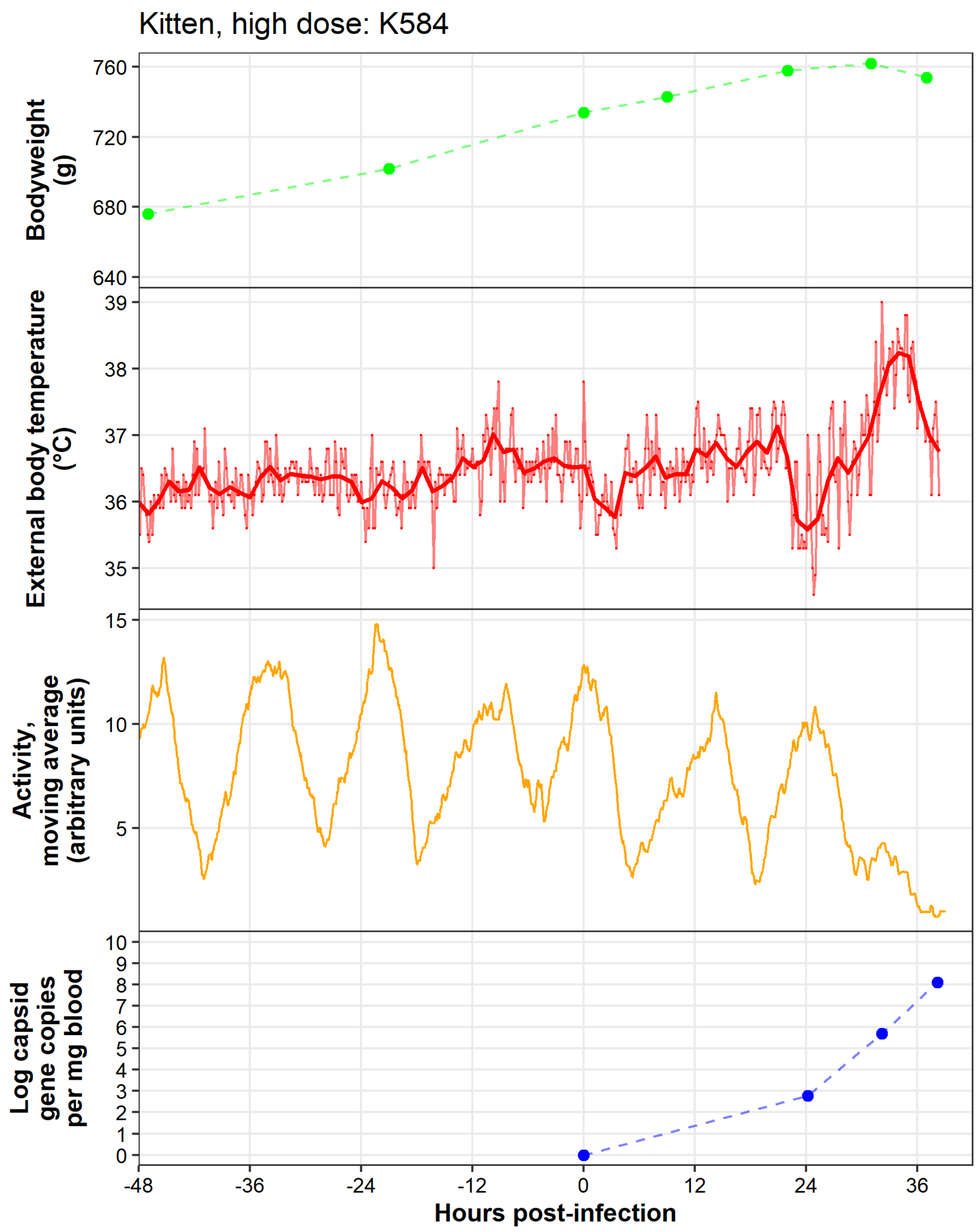

Kitten, high dose: K584
Bodyweight (g)
760
720
680
640
External body temperature (°C)
39
38
37
36
35
Activity, moving average (arbitrary units)
15
10
5
Log capsid gene copies per mg blood
10
9
8
7
6
5
4
3
2
1
0
-48
-36
-24
-12
0
12
24
36
Hours post-infection

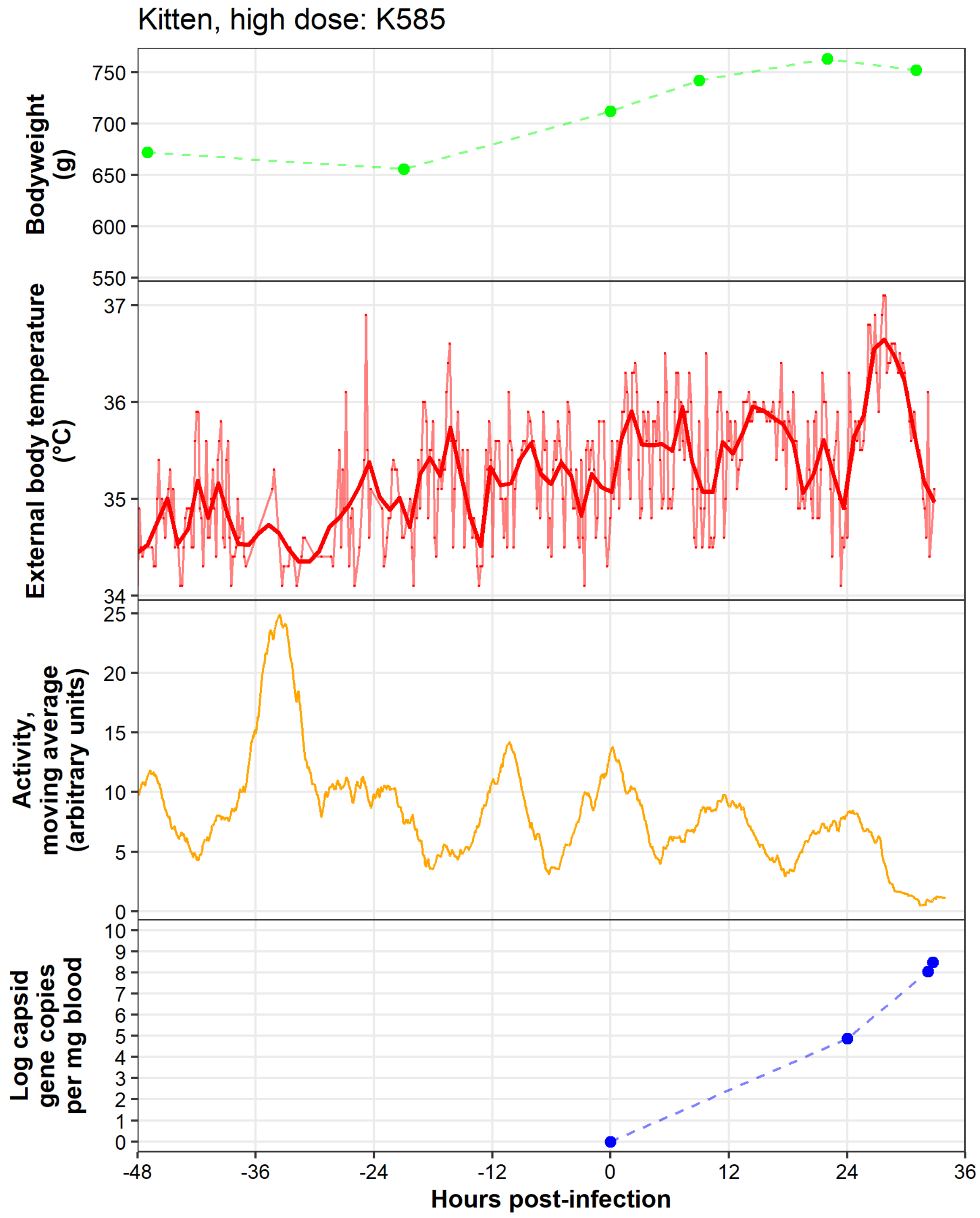

Caveats: Glitch in collar between -36 and -20 hpi.

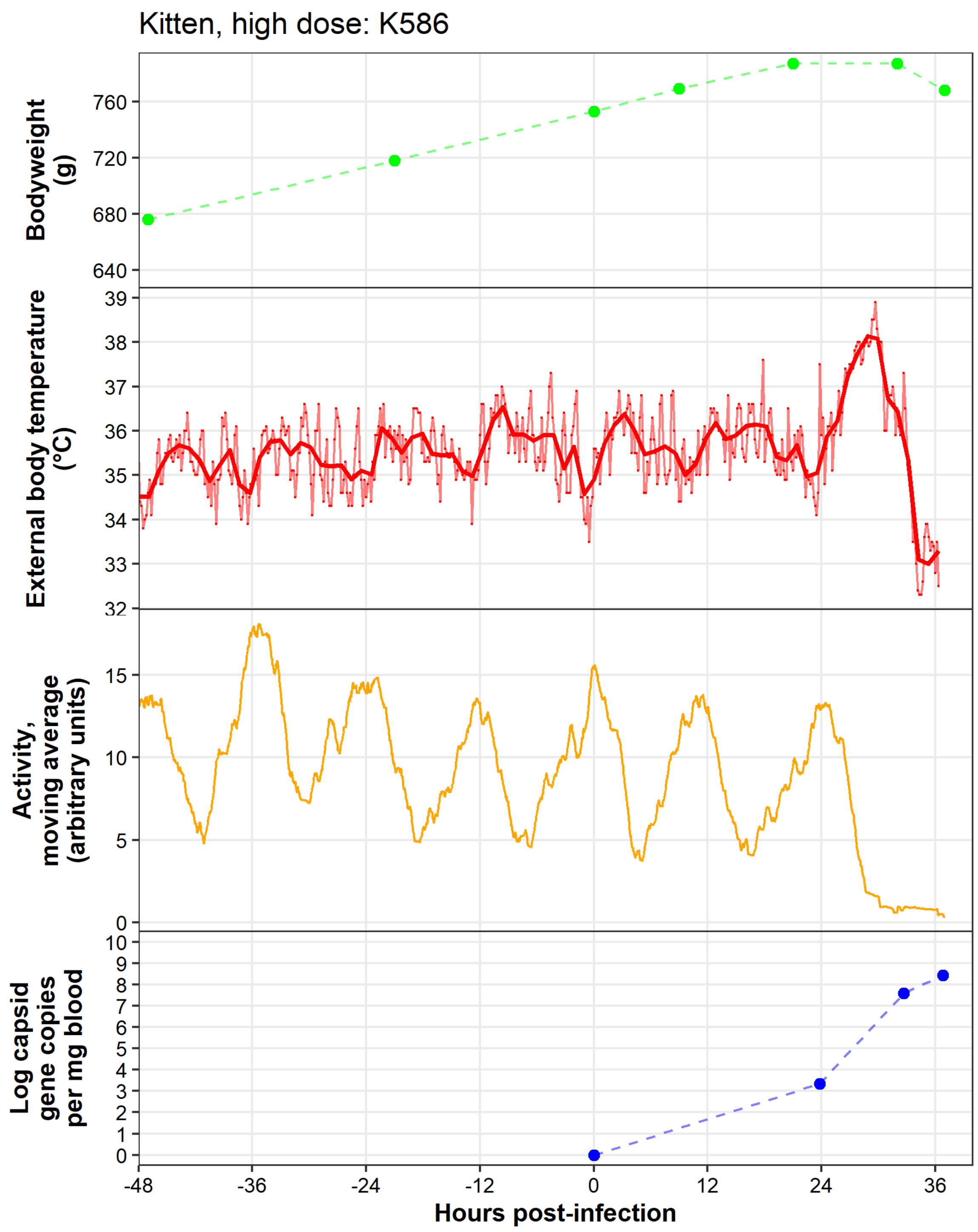

Kitten, high dose: K586
Bodyweight (g)
640
680
720
760
External body temperature (°C)
32
33
34
35
36
37
38
39
Activity, moving average (arbitrary units)
0
5
10
15
Log capsid gene copies per mg blood
0
1
2
3
4
5
6
7
8
9
10
-48
-36
-24
-12
0
12
24
36
Hours post-infection

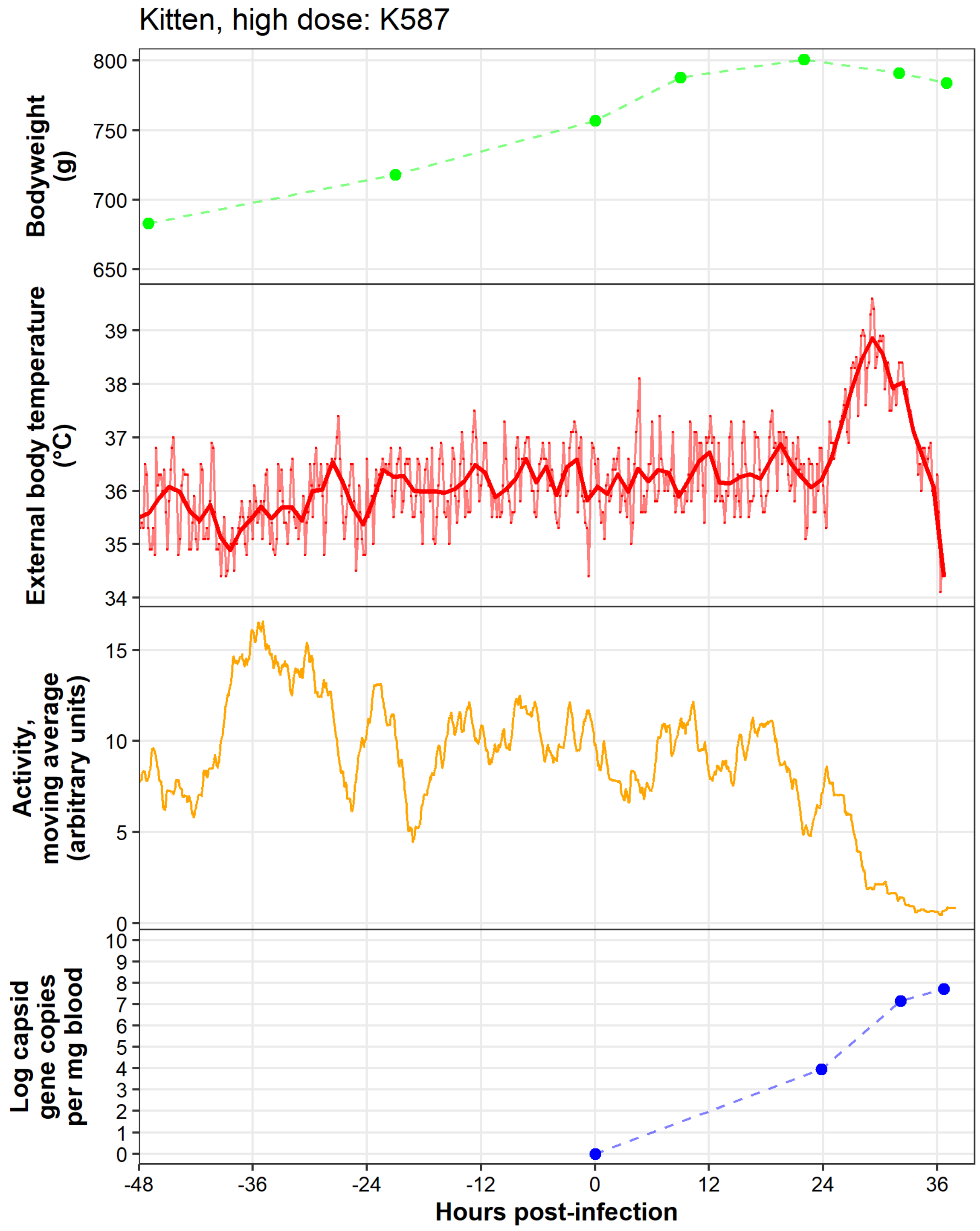

Kitten, high dose: K587
Bodyweight (g)
800
750
700
650
External body temperature (°C)
39
38
37
36
35
34
Activity, moving average (arbitrary units)
15
10
5
0
Log capsid gene copies per mg blood
10
9
8
7
6
5
4
3
2
1
0
-48
-36
-24
-12
0
12
24
36
Hours post-infection

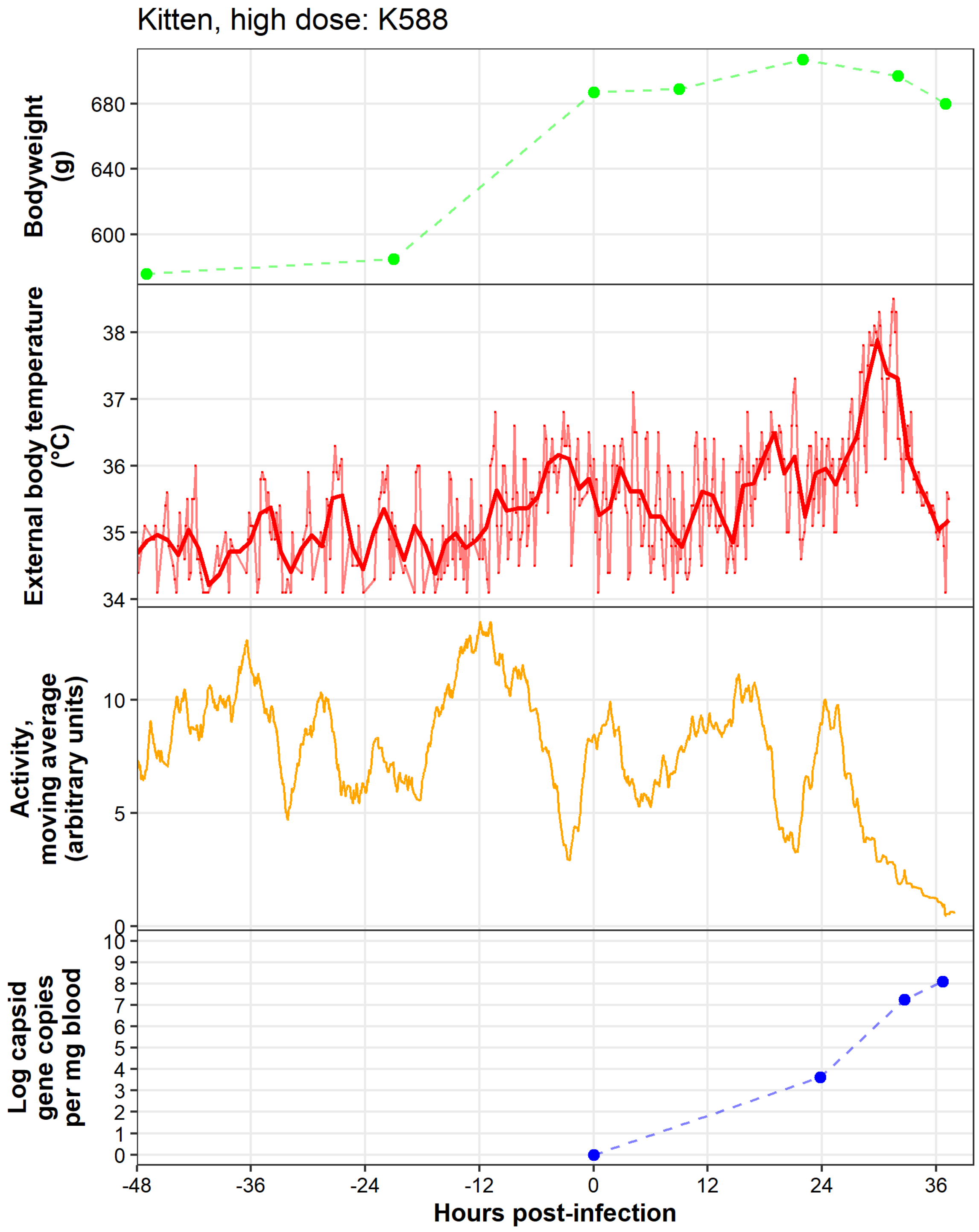
Kitten, high dose: K588
Bodyweight (g)
600
640
680
External body temperature (°C)
34
35
36
37
38
Activity, moving average (arbitrary units)
0
5
10
Log capsid gene copies per mg blood
0
1
2
3
4
5
6
7
8
9
10
-48
-36
-24
-12
0
12
24
36
Hours post-infection

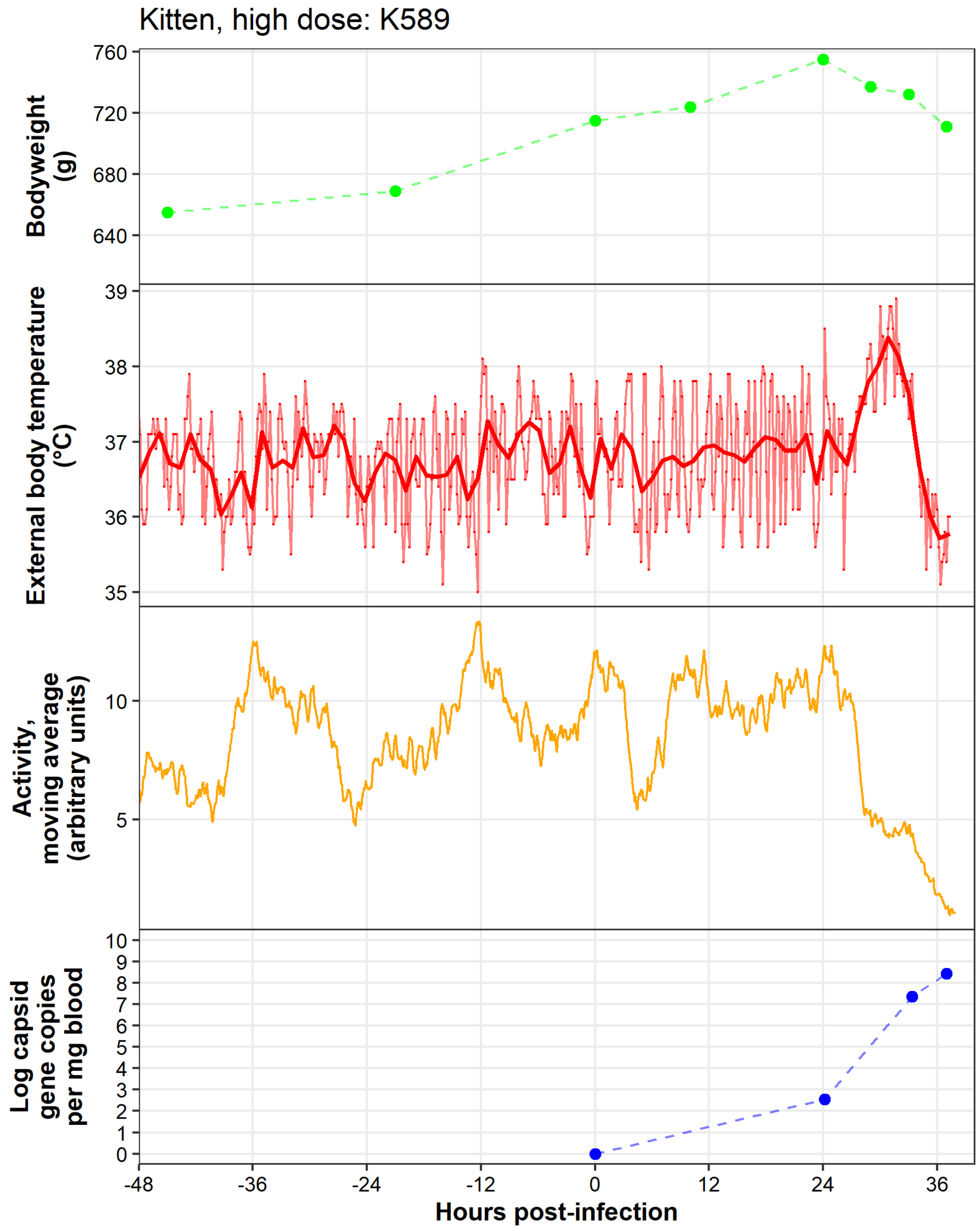
Kitten, high dose: K589
Bodyweight (g)
760
720
680
640
External body temperature (°C)
39
38
37
36
35
Activity, moving average (arbitrary units)
10
5
Log capsid gene copies per mg blood
10
9
8
7
6
5
4
3
2
1
0
-48
-36
-24
-12
0
12
24
36
Hours post-infection

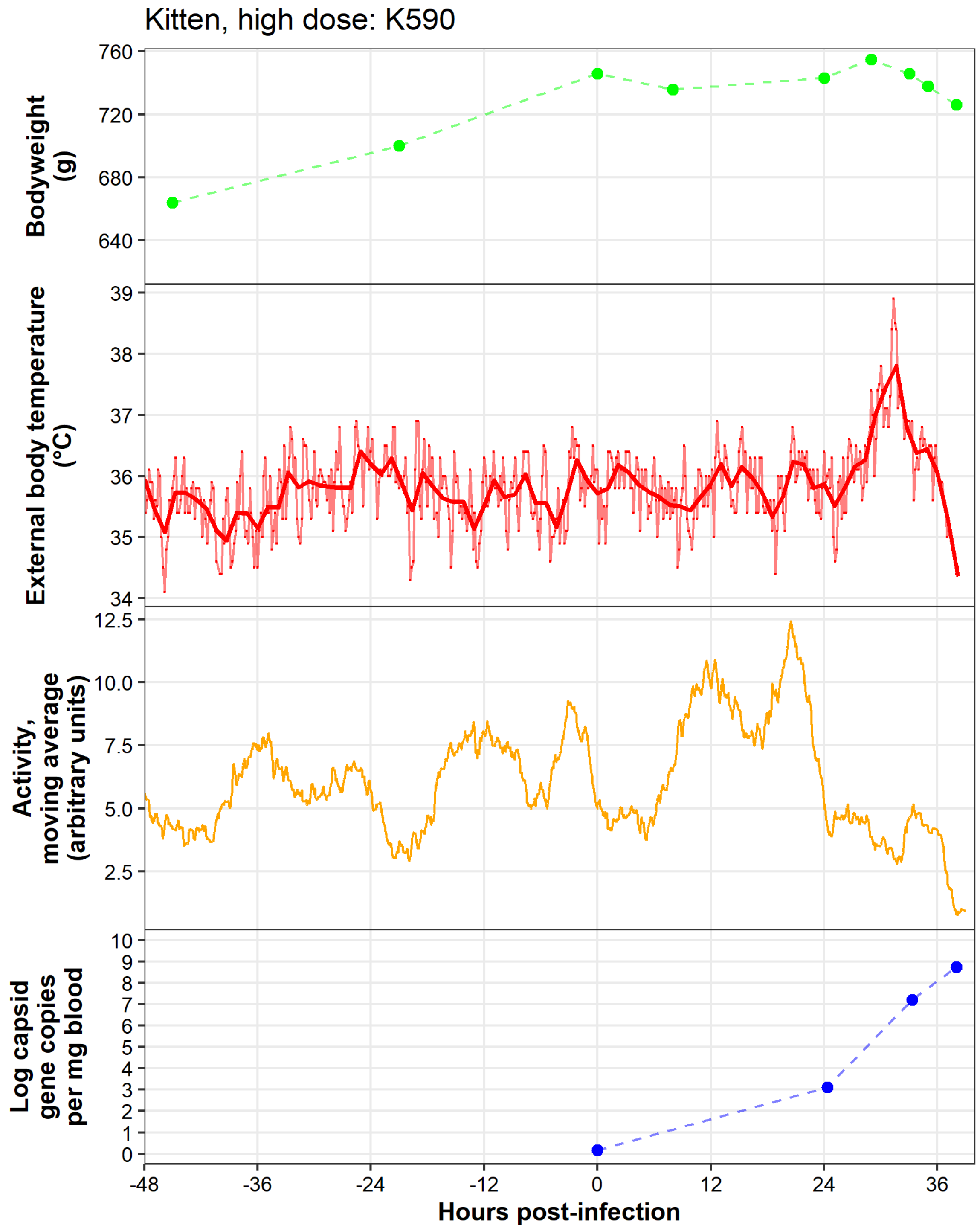

Kitten, high dose: K590
Bodyweight (g)
760
720
680
640
External body temperature (°C)
39
38
37
36
35
34
Activity, moving average (arbitrary units)
12.5
10.0
7.5
5.0
2.5
Log capsid gene copies per mg blood
10
9
8
7
6
5
4
3
2
1
0
-48
-36
-24
-12
0
12
24
36
Hours post-infection

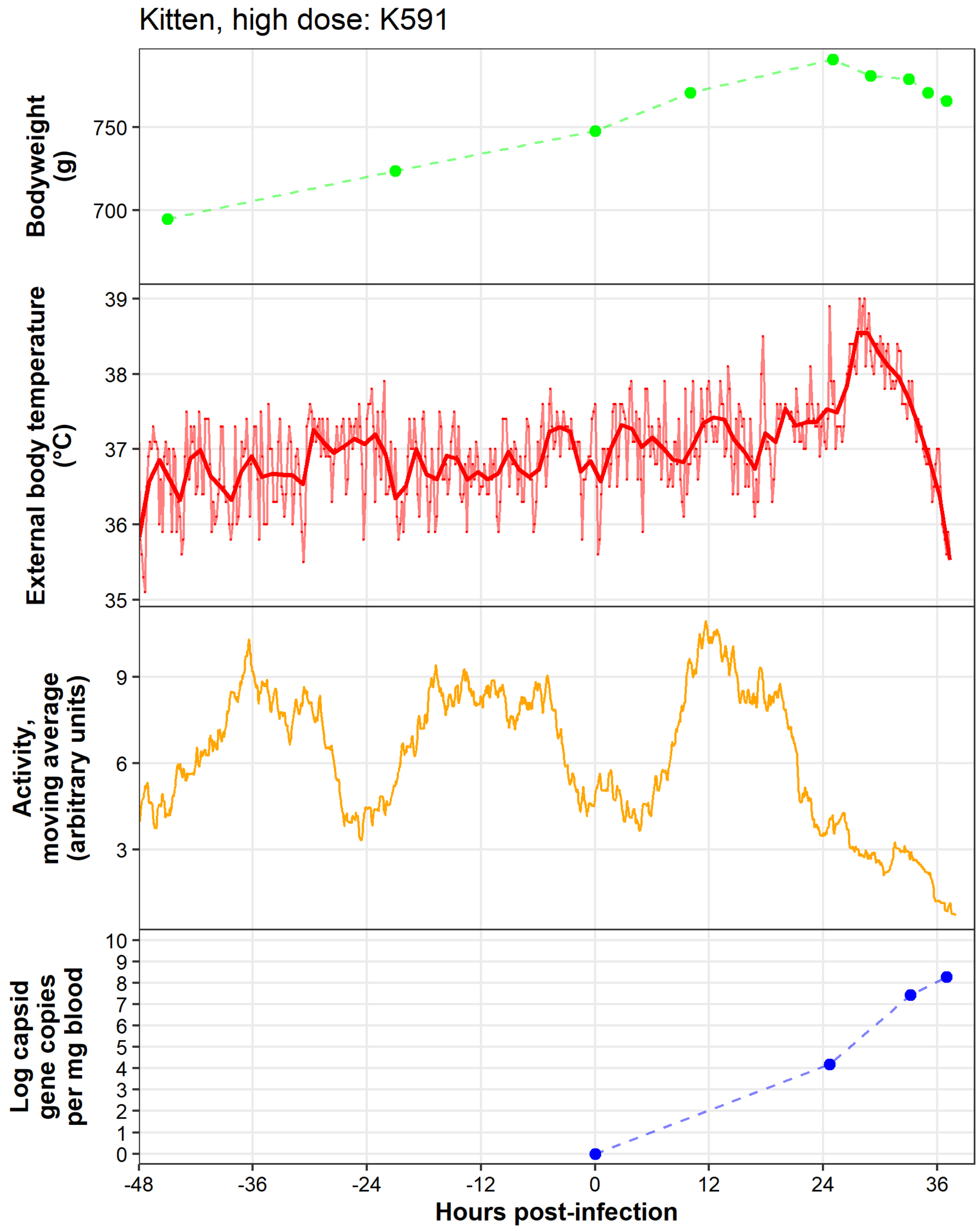

Kitten, high dose: K591
Bodyweight (g)
750
700
External body temperature (°C)
39
38
37
36
35
Activity, moving average (arbitrary units)
9
6
3
Log capsid gene copies per mg blood
10
9
8
7
6
5
4
3
2
1
0
-48
-36
-24
-12
0
12
24
36
Hours post-infection

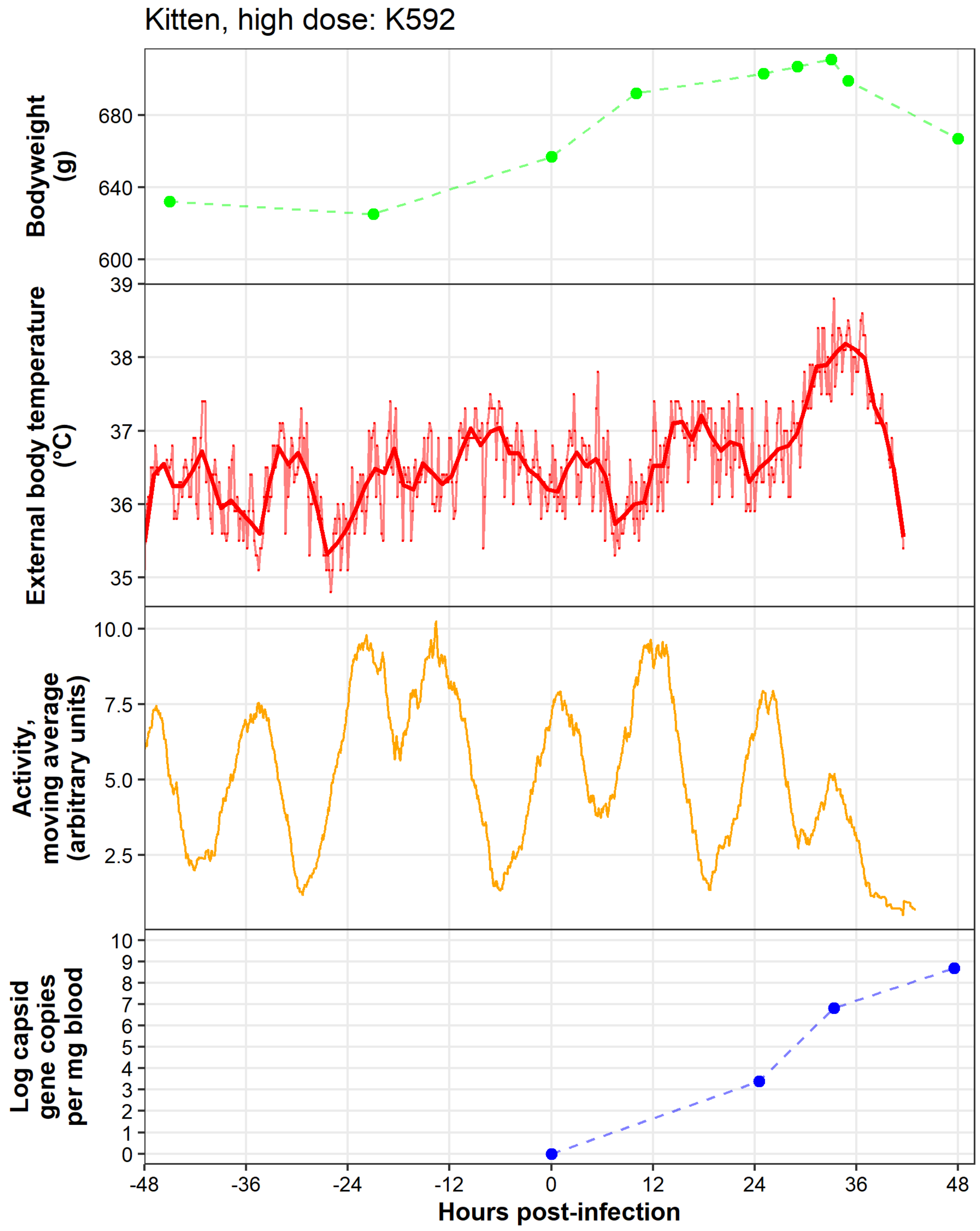

Kitten, high dose: K592
Bodyweight (g)
600
640
680
External body temperature (°C)
35
36
37
38
39
Activity, moving average (arbitrary units)
2.5
5.0
7.5
10.0
Log capsid gene copies per mg blood
0
1
2
3
4
5
6
7
8
9
10
-48
-36
-24
-12
0
12
24
36
48
Hours post-infection

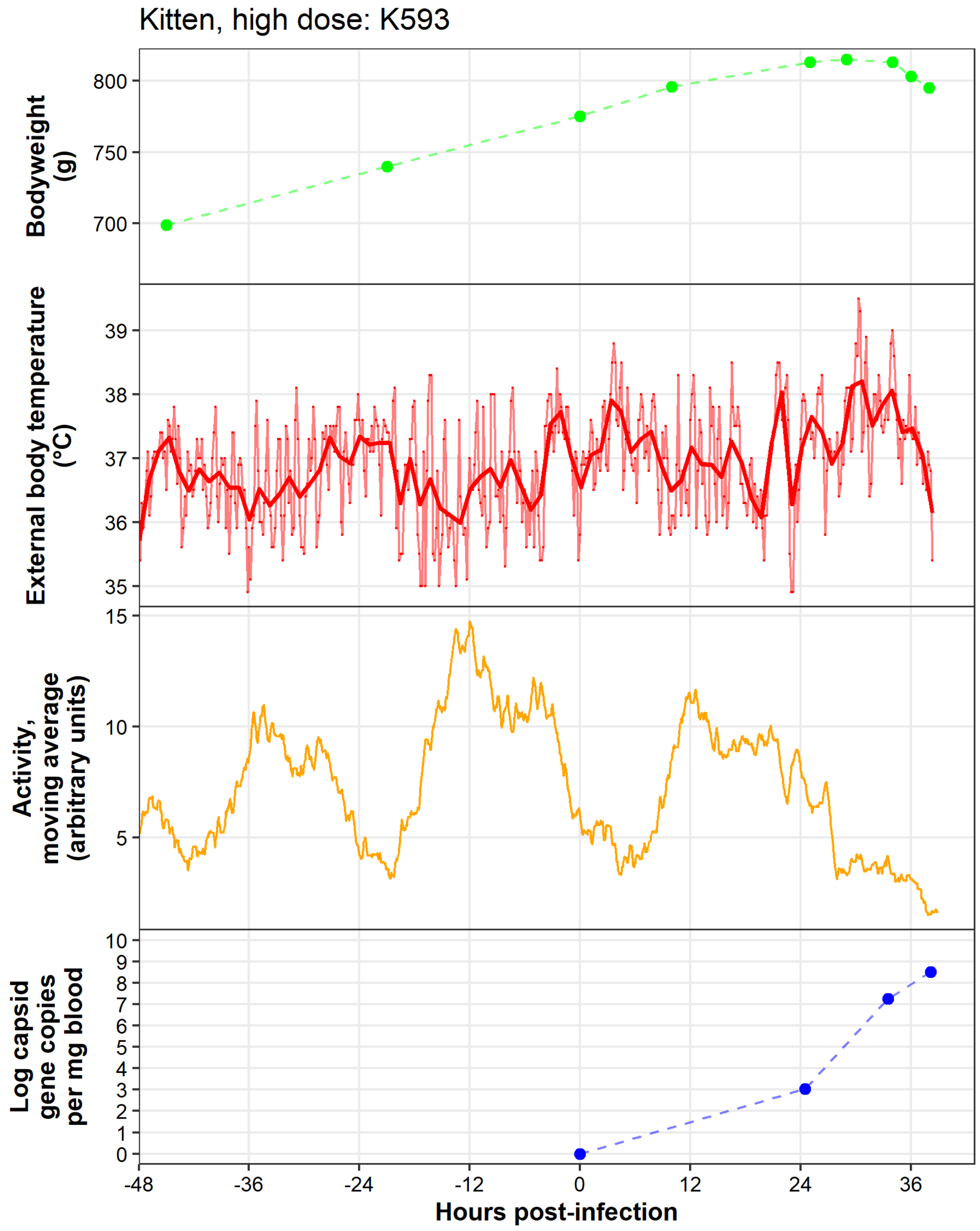

Kitten, high dose: K593
Bodyweight (g)
800
750
700
External body temperature (°C)
39
38
37
36
35
Activity, moving average (arbitrary units)
15
10
5
Log capsid gene copies per mg blood
10
9
8
7
6
5
4
3
2
1
0
-48
-36
-24
-12
0
12
24
36
Hours post-infection

Kitten, high dose: K595

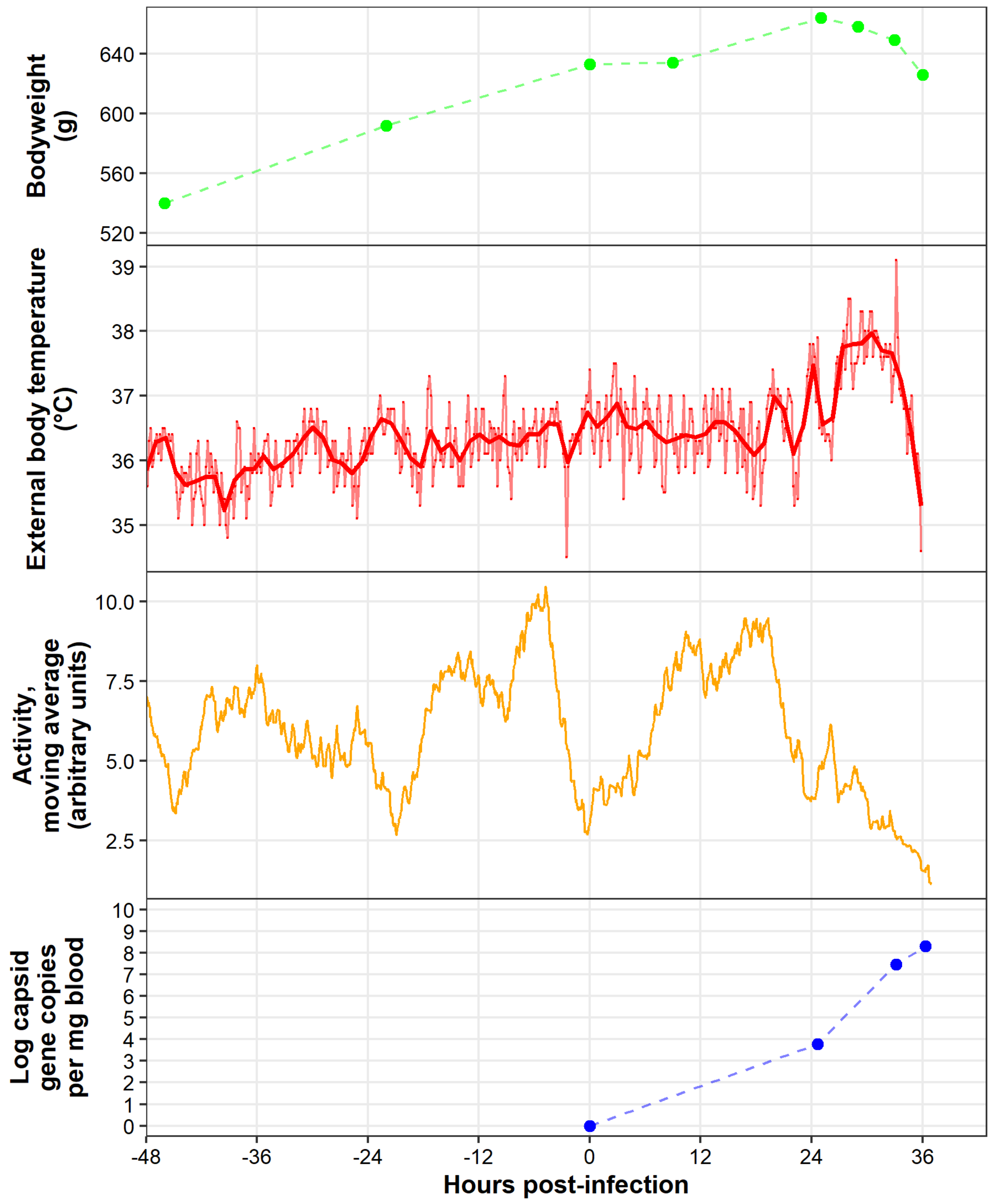

Kitten, low dose

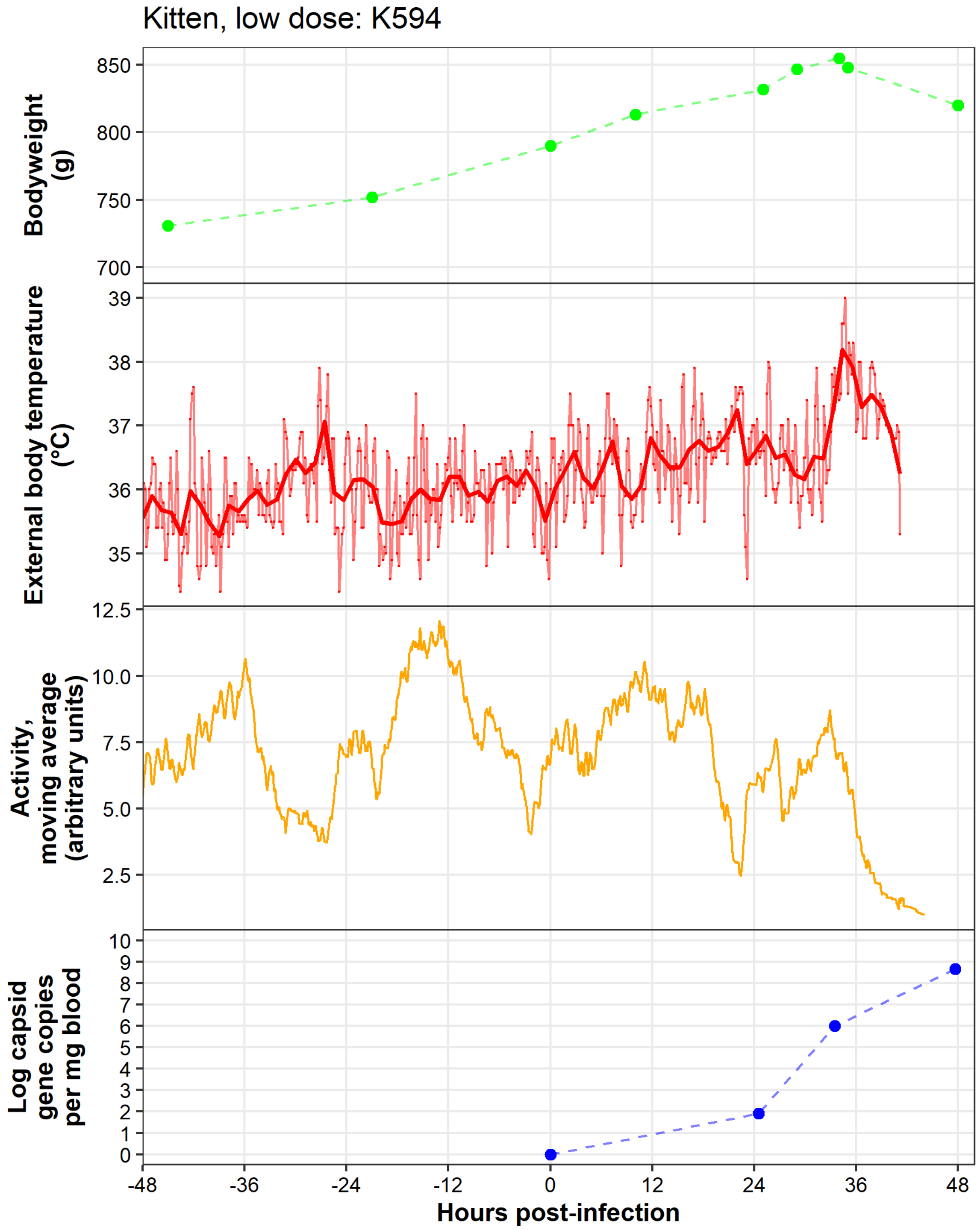

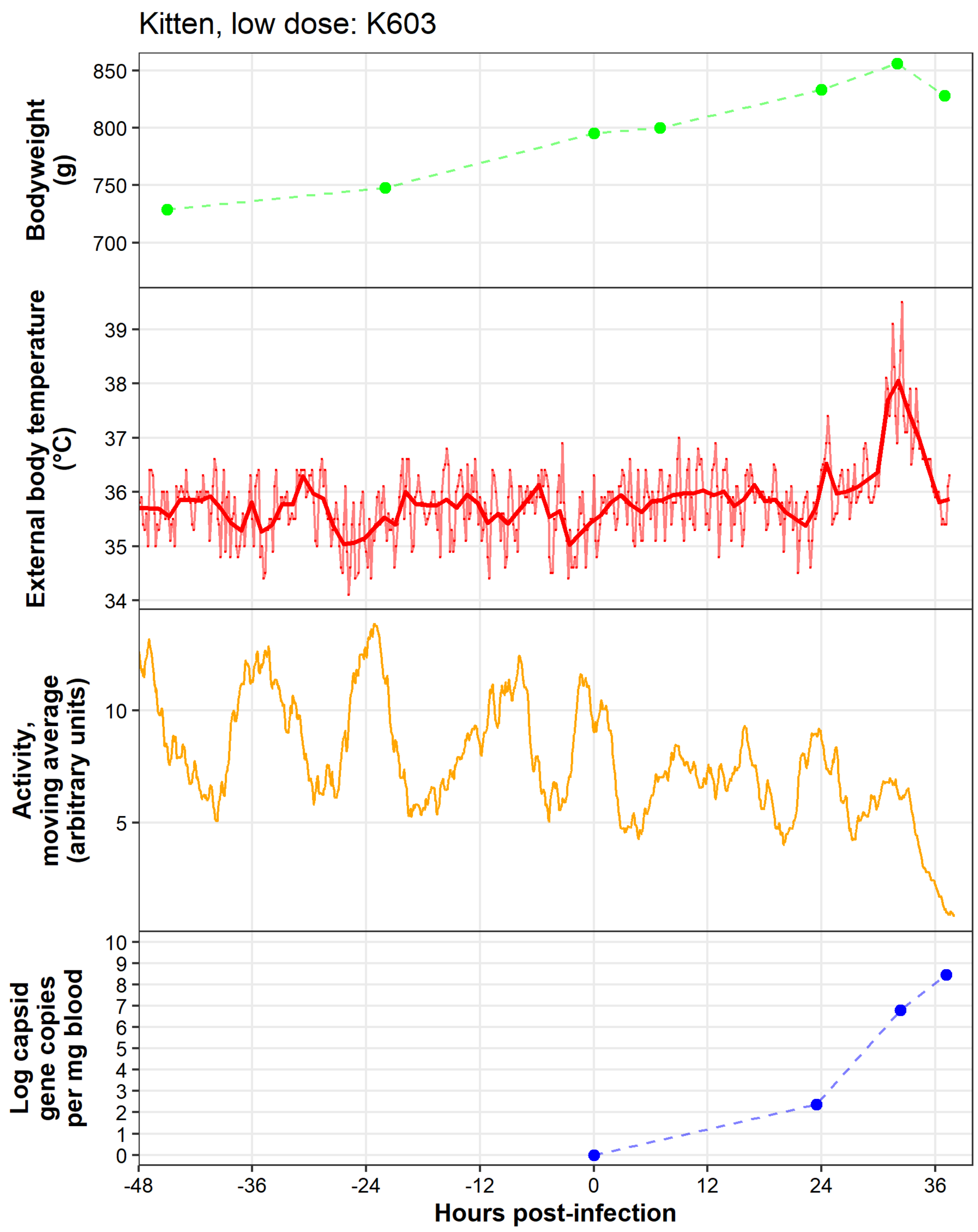

Kitten, low dose: K603
Bodyweight (g)
850
800
750
700
External body temperature (°C)
39
38
37
36
35
34
Activity, moving average (arbitrary units)
10
5
Log capsid gene copies per mg blood
10
9
8
7
6
5
4
3
2
1
0
-48
-36
-24
-12
0
12
24
36
Hours post-infection

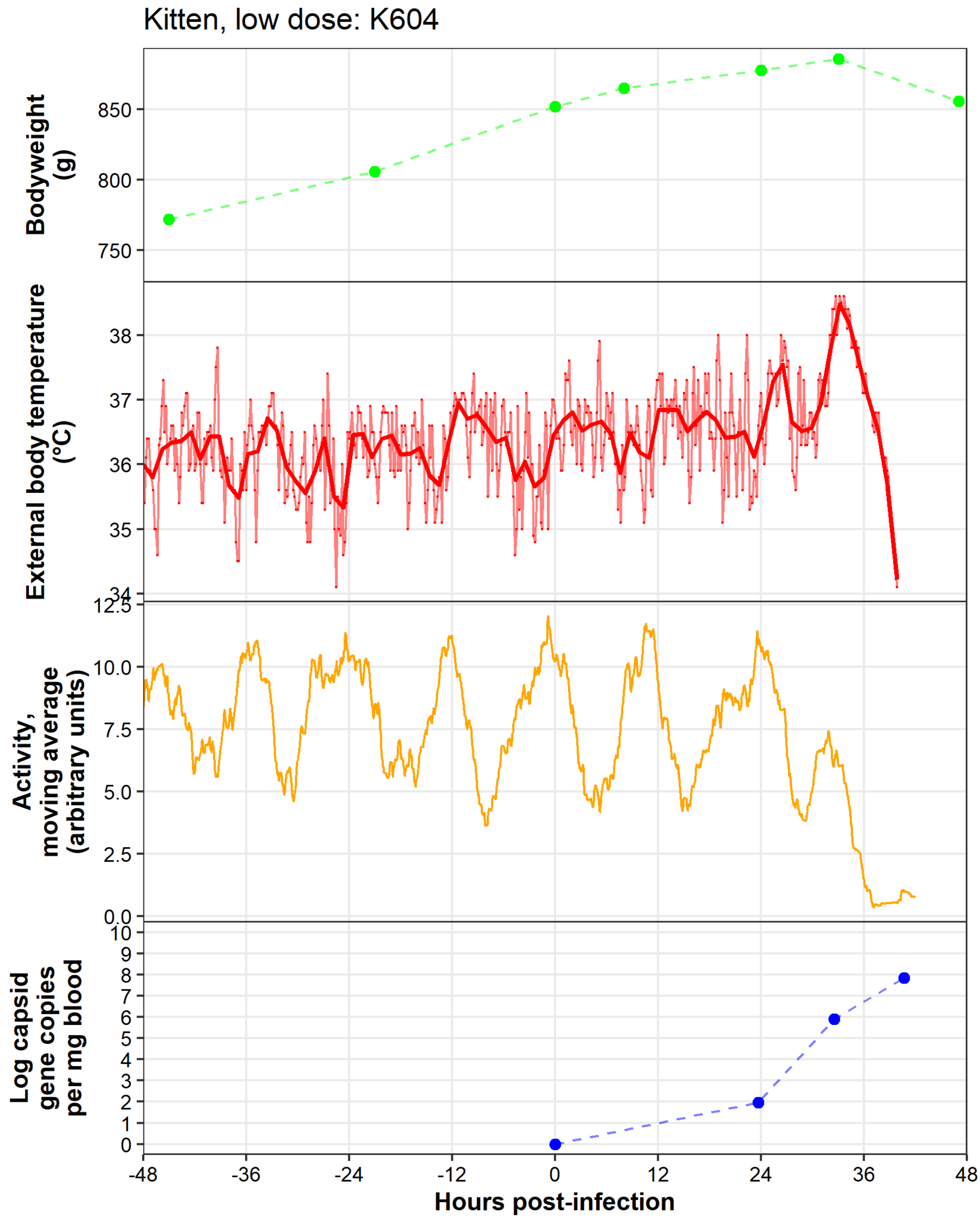

Kitten, low dose: K604
Bodyweight (g)
750
800
850
External body temperature (°C)
34
35
36
37
38
Activity, moving average (arbitrary units)
0.0
2.5
5.0
7.5
10.0
12.5
Log capsid gene copies per mg blood
0
1
2
3
4
5
6
7
8
9
10
-48
-36
-24
-12
0
12
24
36
48
Hours post-infection

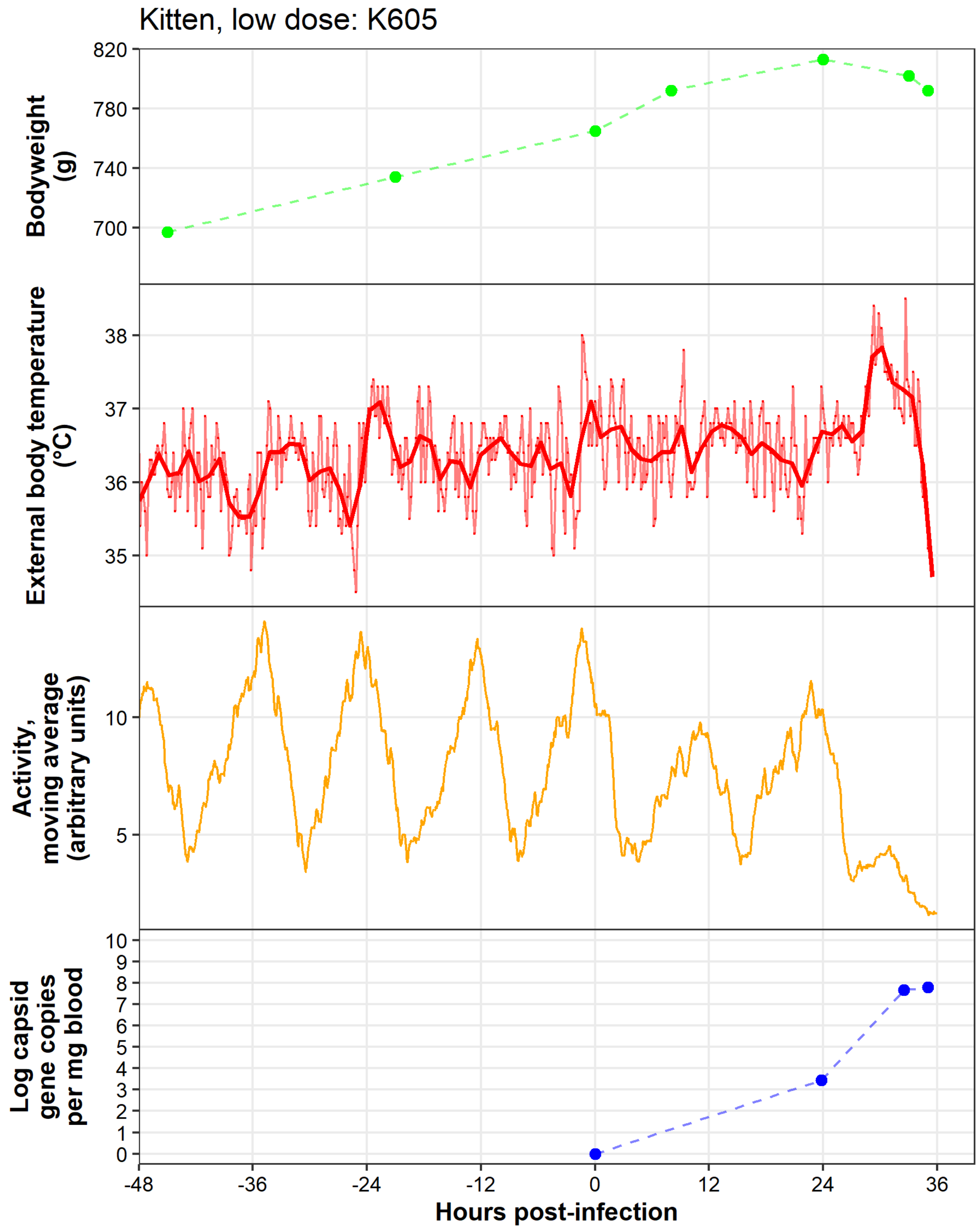

Kitten, low dose: K605
Bodyweight (g)
820
780
740
700
External body temperature (°C)
38
37
36
35
Activity, moving average (arbitrary units)
10
5
Log capsid gene copies per mg blood
10
9
8
7
6
5
4
3
2
1
0
-48
-36
-24
-12
0
12
24
36
Hours post-infection

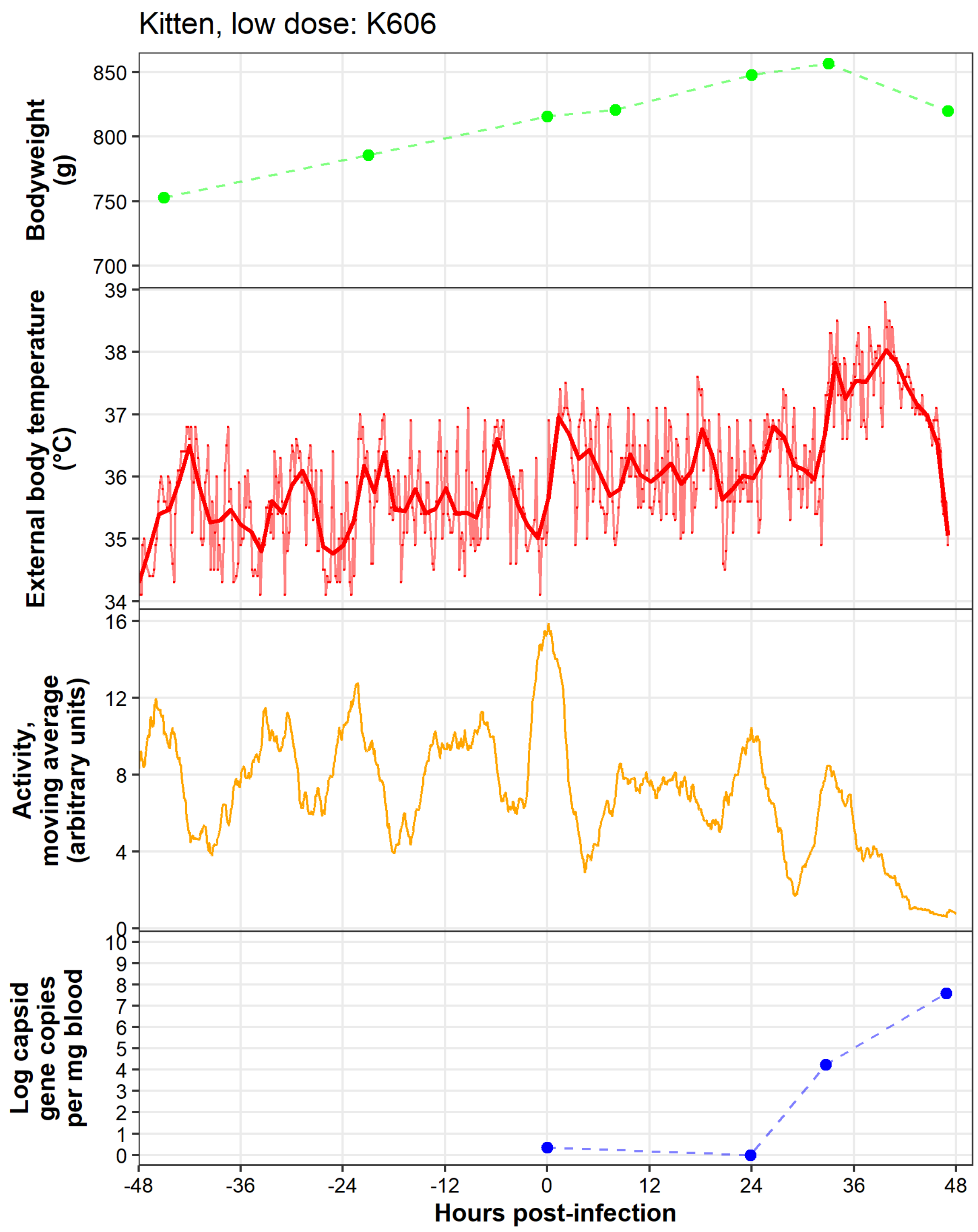

Kitten, low dose: K606
Bodyweight (g)
850
800
750
700
External body temperature (°C)
39
38
37
36
35
34
Activity, moving average (arbitrary units)
16
12
8
4
0
Log capsid gene copies per mg blood
10
9
8
7
6
5
4
3
2
1
0
-48
-36
-24
-12
0
12
24
36
48
Hours post-infection

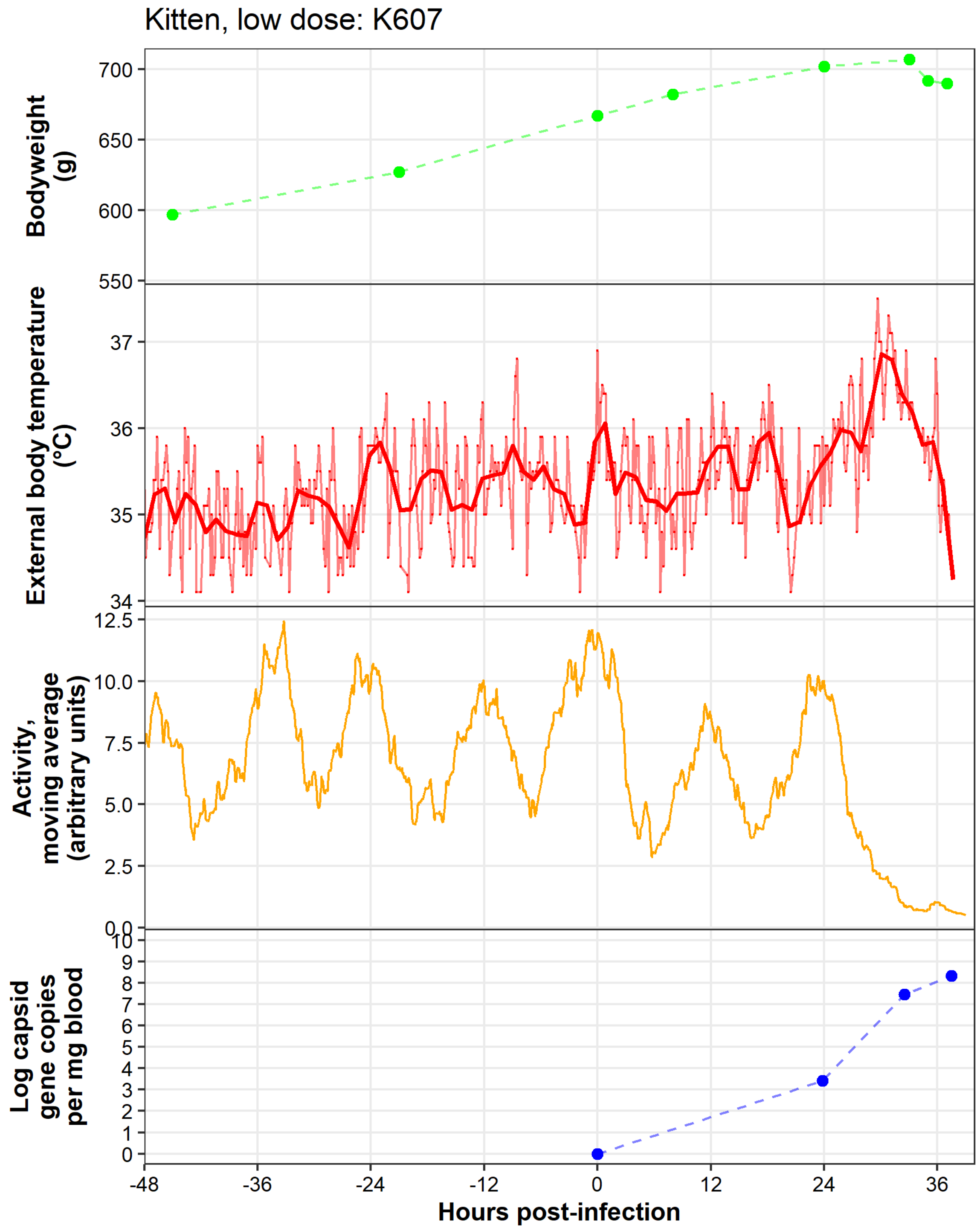

Kitten, low dose: K607
Bodyweight (g)
700
650
600
550
External body temperature (°C)
37
36
35
34
Activity, moving average (arbitrary units)
12.5
10.0
7.5
5.0
2.5
0.0
Log capsid gene copies per mg blood
10
9
8
7
6
5
4
3
2
1
0
-48
-36
-24
-12
0
12
24
36
Hours post-infection

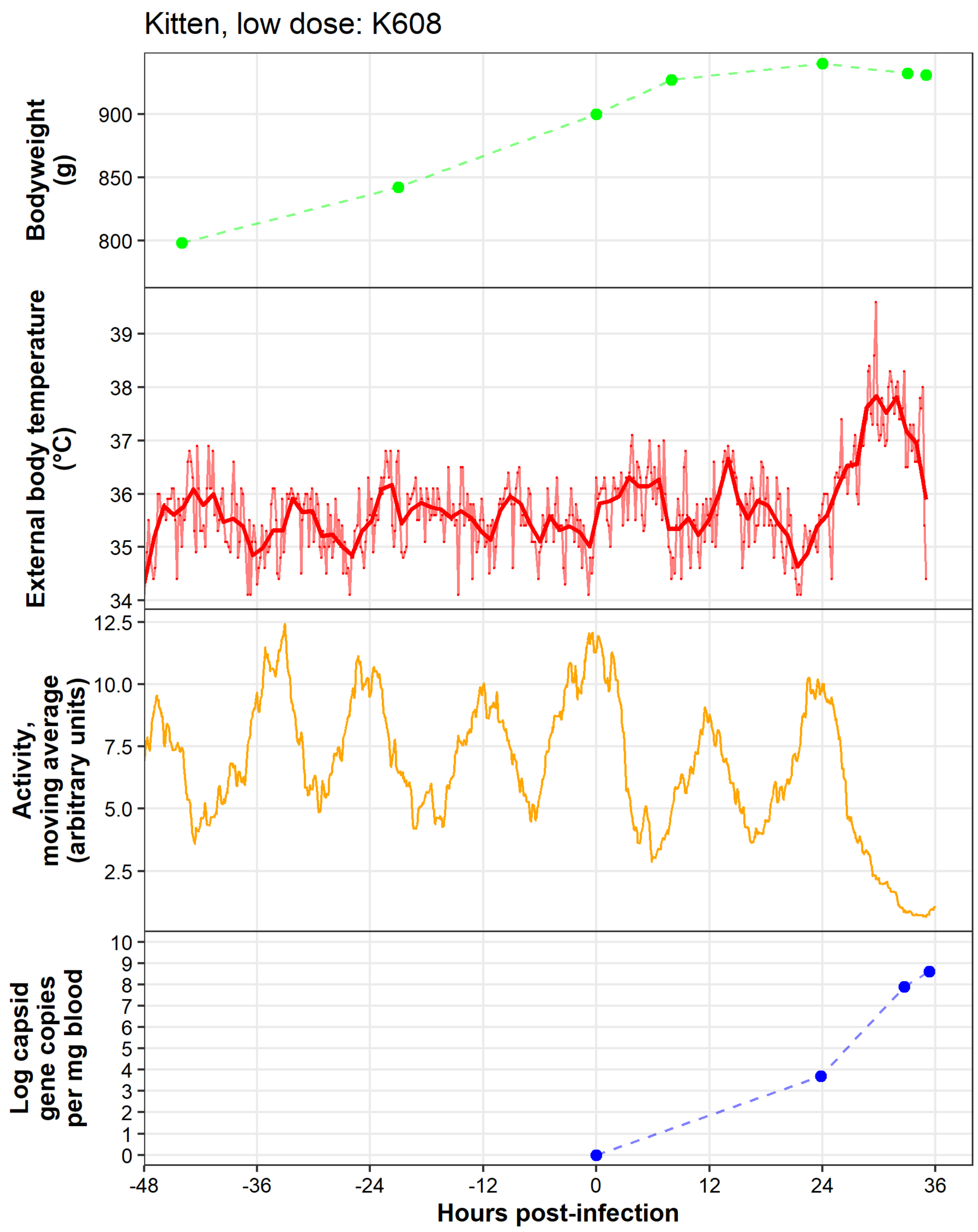
Kitten, low dose: K608
Bodyweight (g)
900
850
800
External body temperature (°C)
39
38
37
36
35
34
Activity, moving average (arbitrary units)
12.5
10.0
7.5
5.0
2.5
Log capsid gene copies per mg blood
10
9
8
7
6
5
4
3
2
1
0
-48
-36
-24
-12
0
12
24
36
Hours post-infection

Kitten, low dose: K610

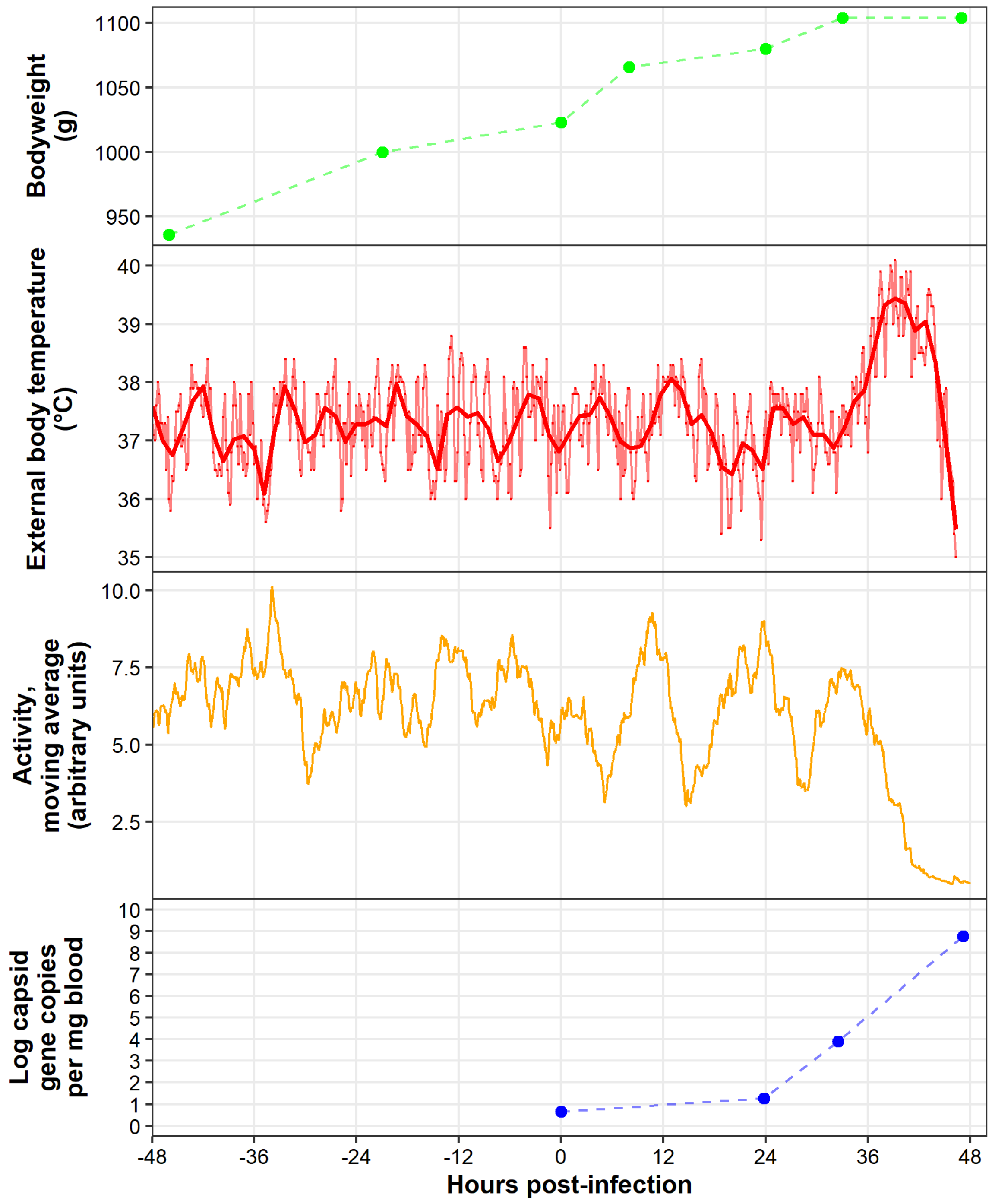

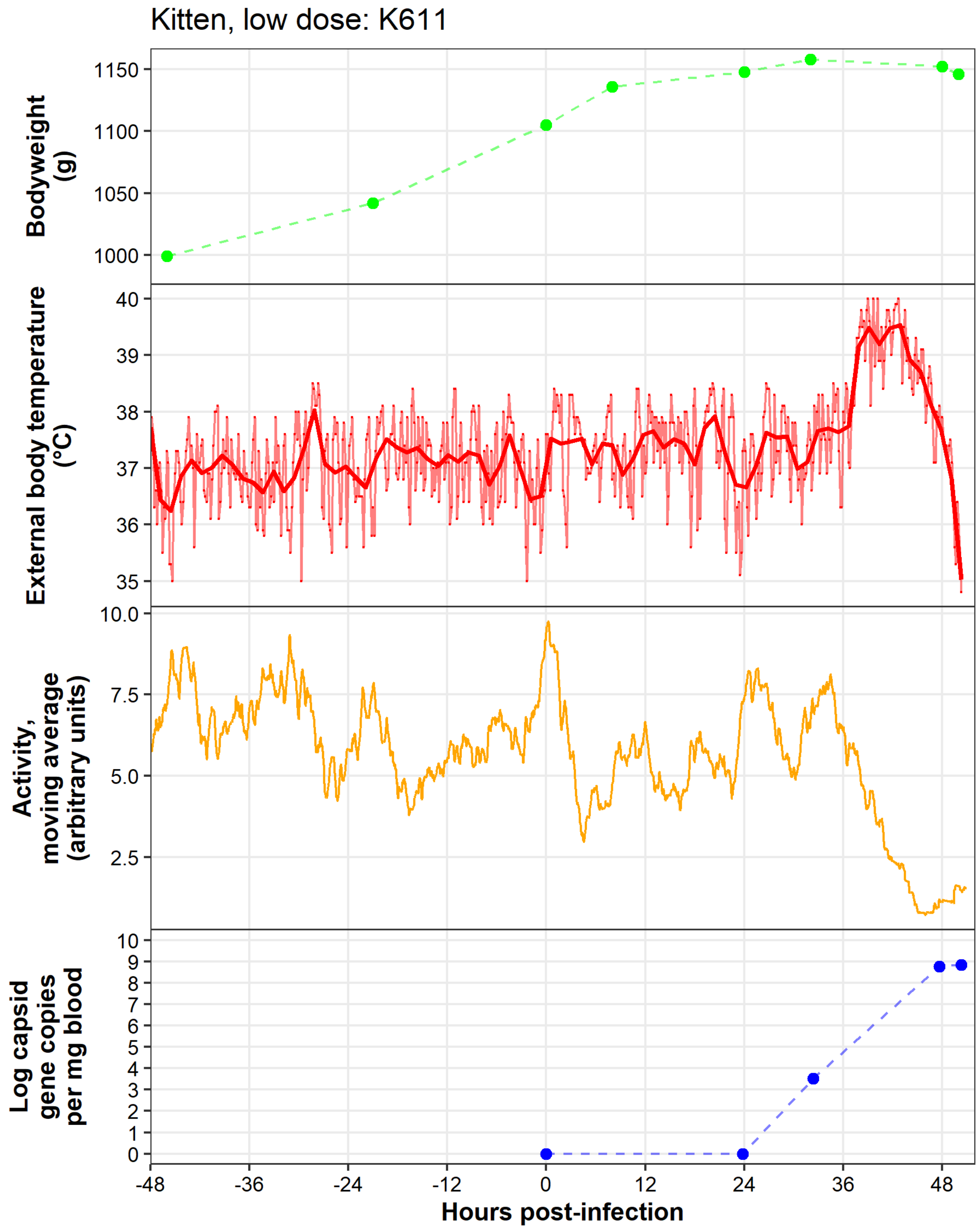
Kitten, low dose: K611
Bodyweight (g)
1150
1100
1050
1000
External body temperature (°C)
40
39
38
37
36
35
Activity, moving average (arbitrary units)
10.0
7.5
5.0
2.5
Log capsid gene copies per mg blood
10
9
8
7
6
5
4
3
2
1
0
-48
-36
-24
-12
0
12
24
36
48
Hours post-infection

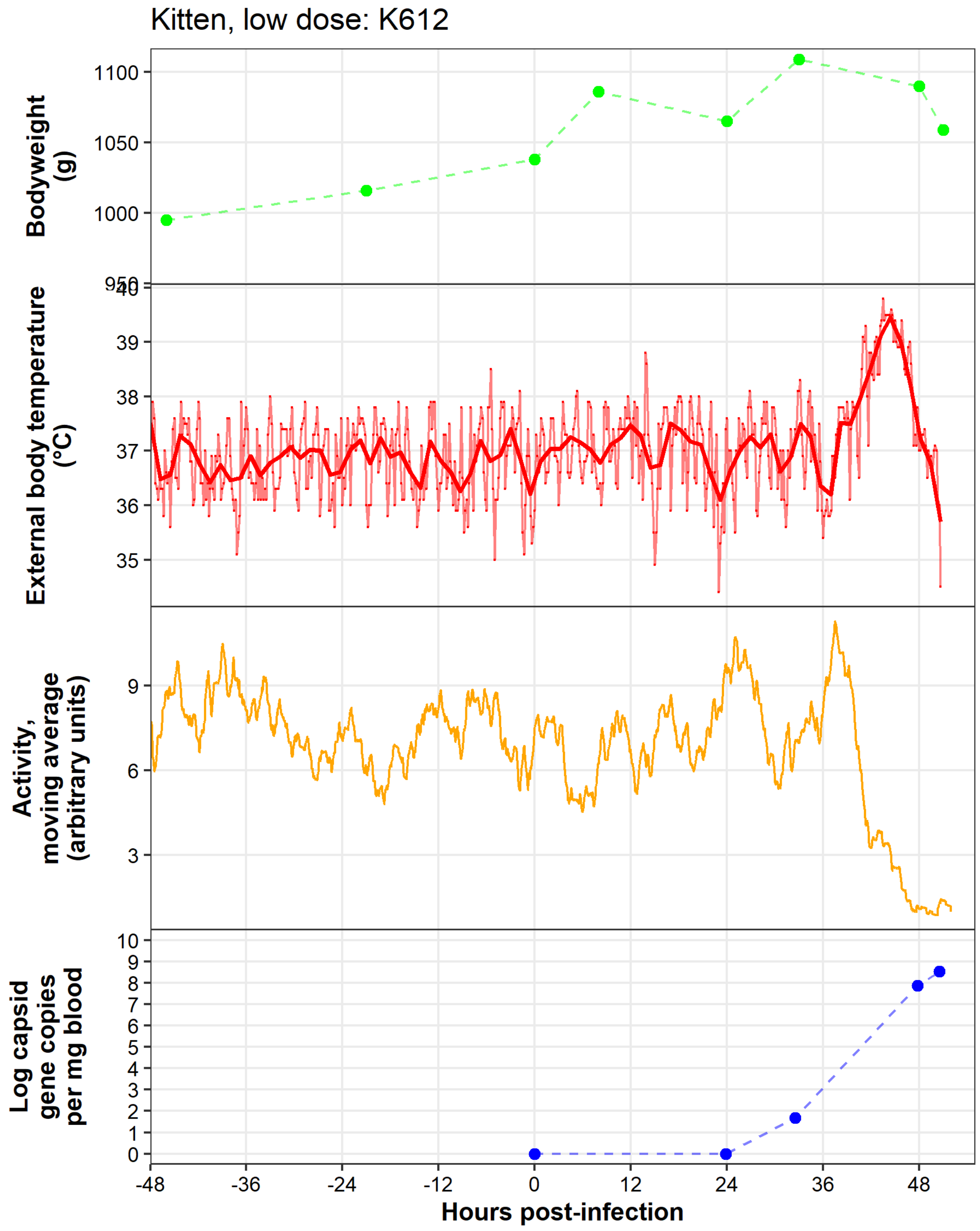
Kitten, low dose: K612
Bodyweight (g)
1100
1050
1000
950
External body temperature (°C)
40
39
38
37
36
35
Activity, moving average (arbitrary units)
9
6
3
Log capsid gene copies per mg blood
10
9
8
7
6
5
4
3
2
1
0
-48
-36
-24
-12
0
12
24
36
48
Hours post-infection

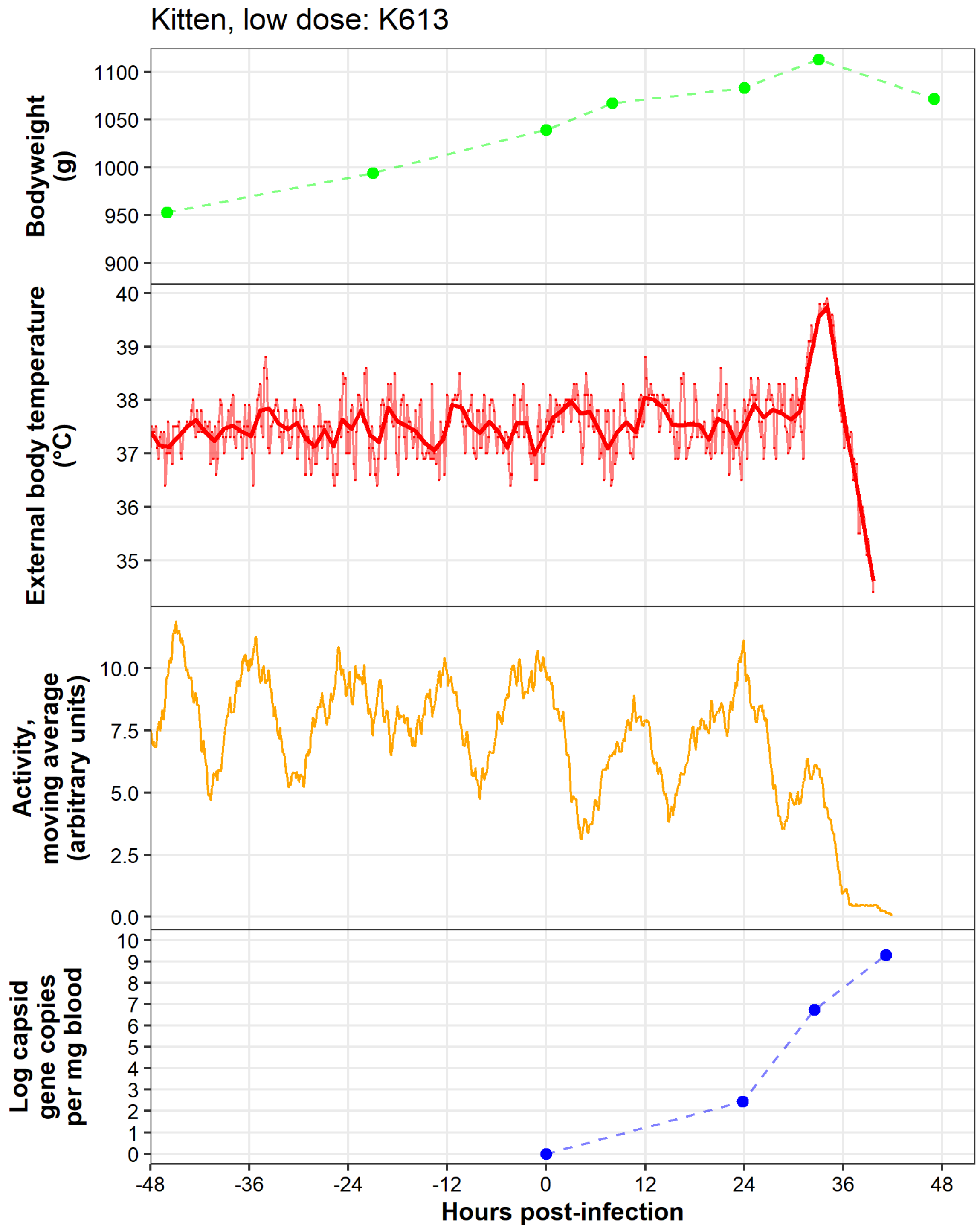

Kitten, low dose: K613
Bodyweight (g)
1100
1050
1000
950
900
External body temperature (°C)
40
39
38
37
36
35
Activity, moving average (arbitrary units)
10.0
7.5
5.0
2.5
0.0
Log capsid gene copies per mg blood
10
9
8
7
6
5
4
3
2
1
0
-48
-36
-24
-12
0
12
24
36
48
Hours post-infection

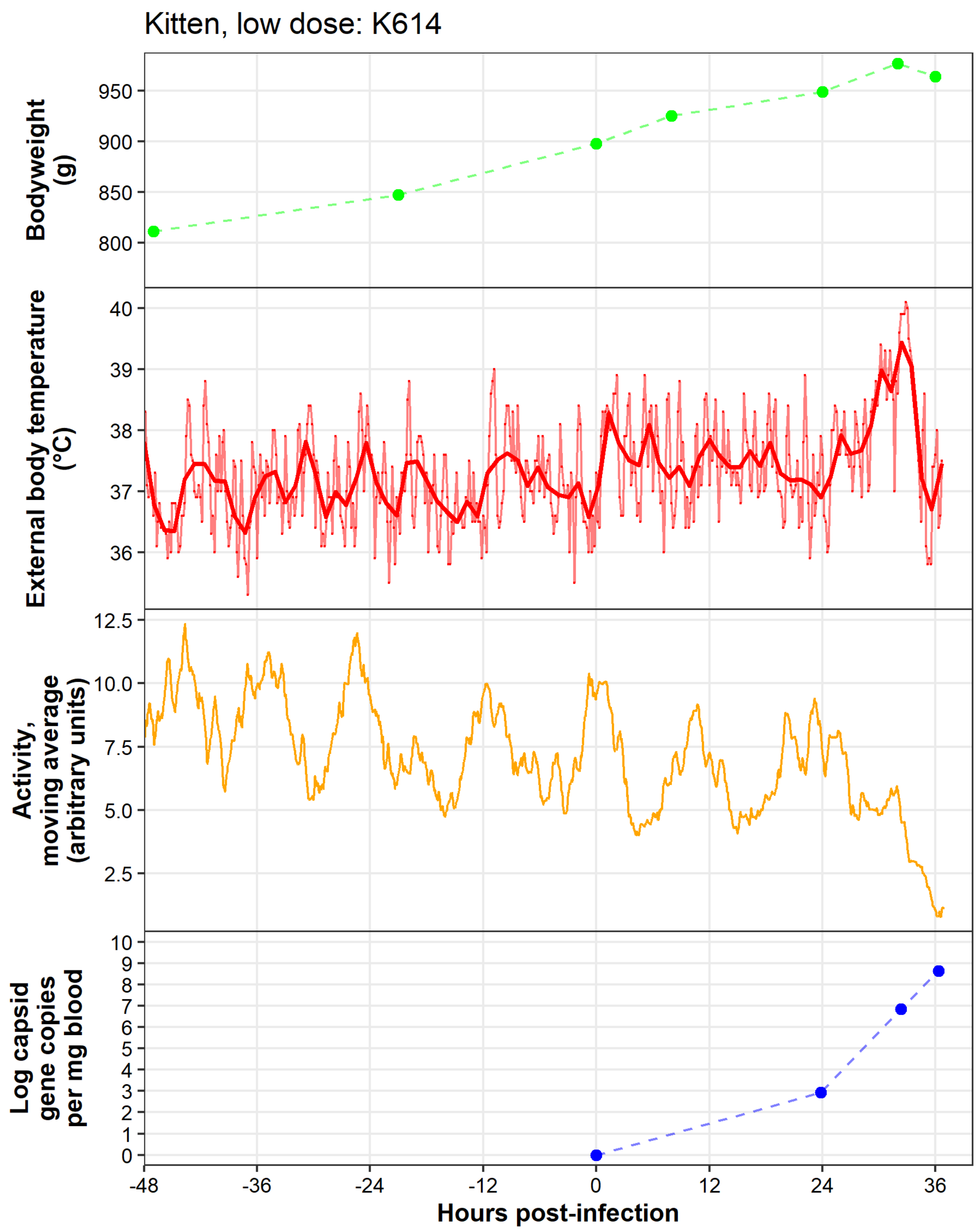

Kitten, low dose: K614
Bodyweight (g)
950
900
850
800
External body temperature (°C)
40
39
38
37
36
Activity, moving average (arbitrary units)
12.5
10.0
7.5
5.0
2.5
Log capsid gene copies per mg blood
10
9
8
7
6
5
4
3
2
1
0
-48
-36
-24
-12
0
12
24
36
Hours post-infection
